# Pharmacological inhibition of UBASH3B induces mitotic arrest and selectively reduces tumor growth

**DOI:** 10.1038/s44321-026-00519-0

**Published:** 2026-09-22

**Authors:** Evanthia Pangou, Sushil Awal, Mathieu Meode, Charlotte Kleiss, Mehdi Cloarec, Erwan Grandgirard, Karl Vivot, Lucile Guerber, Ksenia Krupina, Katerina Jerabkova-Roda, Paulo J da Costa, Fabien Alpy, Arthur Martinet, Massimo Lodi, Alexandra Cousido-Siah, Olivia Wendling, Romain Hany, Stéphanie Riché, François Daubeuf, Patrick Gizzi, Pascal Villa, Yves Nominé, Catherine Tomasetto, Dominique Bonnet, Izabela Sumara

**Affiliations:** 1 Institut de Génétique et de Biologie Moléculaire et Cellulaire (IGBMC), Illkirch, France; 2 Centre National de la Recherche Scientifique UMR 7104, Strasbourg, France; 3 Institut National de la Santé et de la Recherche Médicale U 1258, Strasbourg, France; 4 Université de Strasbourg, Strasbourg, France; 5 Centre de Recherche en Biologie Cellulaire de Montpellier (CRBM), CNRS UMR 5237, Université de Montpellier, Montpellier, France; 6 Department of Biochemistry and Molecular Biology, University of Iowa, Iowa City, IA USA; 7 Tumor Biomechanics, INSERM UMR_S1109, Strasbourg, France; 8 Université de Strasbourg, Institut de Biologie Moléculaire et Cellulaire, CNRS UPR9002, Strasbourg, France; 9 PHEN-ICS, CNRS UAR2062, Inserm US66, Unistra, Illkirch, France; 10 PCBIS Plateforme de Chimie Biologique Intégrative de Strasbourg, UAR3286, CNRS/Université de Strasbourg, Strasbourg, France; 11 Laboratoire d’Innovation Thérapeutique, Faculté de Pharmacie, UMR7200 CNRS/Université de Strasbourg, Illkirch, France

**Keywords:** Cancer, Cell Cycle, Pharmacology & Drug Discovery

## Abstract

Cancer cells tolerate mitotic errors to sustain proliferation, yet the molecular dependencies enabling this tolerance remain poorly understood and largely unexploited therapeutically. UBASH3B ensures mitotic fidelity by regulating Aurora B localization and is overexpressed in tumors. We investigated whether pharmacological disruption of this pathway exposes a mitotic dependency in cancer. We demonstrate that the UBASH3B 2HP domain represents a druggable interface for disrupting UBASH3B-dependent Aurora B regulation. We identify UBASHIN, a small-molecule inhibitor targeting this domain. UBASHIN phenocopies UBASH3B depletion, causing Aurora B mislocalization, mitotic arrest and mitotic cell death. UBASHIN impairs UBASH3B localization to the mitotic spindle and selectively inhibits cancer cell proliferation while sparing non-cancerous cells. UBASH3B protein levels and the cellular ability to sustain mitotic arrest, but not global aneuploidy, predict UBASHIN sensitivity, supporting a cancer-specific on-target mechanism. In colorectal and triple-negative breast cancer mouse models, UBASHIN reduces tumor volume comparably to docetaxel, without detectable toxicity in wild-type animals. These findings identify the UBASH3B pathway as a cancer-specific mitotic vulnerability and highlight its pharmacological inhibition as a selective therapeutic strategy.

## Synopsis

Small molecule UBASHIN disrupts mitotic localization of UBASH3B and Aurora B, inducing arrest and cell death during mitosis. UBASHIN selectively inhibits tumor growth without affecting normal tissues, revealing a cancer-specific mitotic vulnerability.

- UBASHIN inhibits mitotic localization of UBASH3B and Aurora B.
- UBASHIN triggers arrest and cell death during mitosis.
- UBASHIN induces cancer cell death without toxicity to normal cells.
- UBASHIN selectively suppresses breast and colon tumor growth in vivo.

## Abstract (web summary)

Small molecule UBASHIN disrupts mitotic localization of UBASH3B and Aurora B, inducing arrest and cell death during mitosis. UBASHIN selectively inhibits tumor growth without affecting normal tissues, revealing a cancer-specific mitotic vulnerability.

## Graphical abstract

**Figure:**
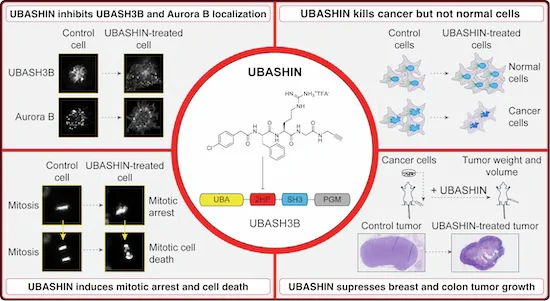

The paper explained **Problem**

Cancer cells frequently accumulate mitotic errors yet continue to proliferate, suggesting they rely on specific regulators to tolerate this instability. UBASH3B is a ubiquitin receptor for the mitotic kinase Aurora B that controls its localization during mitosis and is overexpressed in aggressive cancers including triple-negative breast and colorectal cancer. Crucially, mice lacking UBASH3B develop normally, raising the possibility that UBASH3B represents a cancer-specific mitotic dependency. However, no pharmacological tool existed to exploit this vulnerability, and the molecular basis of UBASH3B mitotic function remained incompletely understood.

**Results**

We identify the 2HP domain of UBASH3B as a druggable interface required for its localization to the mitotic spindle and for proper Aurora B positioning during mitosis. Chemical screening identified UBASHIN, a small-molecule inhibitor that preferentially engages the 2HP domain. UBASHIN disrupts UBASH3B and Aurora B mitotic localization, triggers mitotic arrest, and induces cancer cell death, closely phenocopying genetic depletion of UBASH3B. UBASHIN sensitivity correlates with UBASH3B expression and the ability to sustain mitotic arrest, but not with basal aneuploidy, supporting an on-target, cancer-specific mechanism. In breast and colorectal cancer mouse models, UBASHIN reduces tumor growth comparably to docetaxel, a standard chemotherapy agent, with less tissue necrosis and no detectable toxicity in healthy animals.

**Impact**

This study establishes UBASH3B-dependent Aurora B regulation as a targetable mitotic vulnerability in cancer and provides preclinical proof-of-concept for its pharmacological inhibition. By exploiting a cancer-specific dependency rather than broadly disrupting the mitotic machinery, UBASHIN offers a potentially more selective antimitotic strategy than classical taxanes or Aurora B inhibitors. These findings lay the groundwork for further optimization of UBASHIN derivatives and their clinical evaluation in aggressive malignancies including triple-negative breast and colorectal cancer.

## Introduction

Mitosis ensures the equal segregation of chromosomes into daughter cells (McIntosh, 2016), and its fidelity is monitored by the Spindle Assembly Checkpoint (SAC), which delays anaphase until kinetochores have achieved proper microtubule attachment (McAinsh and Kops, 2023). Failures in these processes lead to chromosomal instability (CIN), a hallmark of many cancers characterized by frequent chromosome missegregation events (Devillers et al, 2024). Paradoxically, despite high levels of CIN, cancer cells often retain sufficient checkpoint function to avoid lethal segregation errors while sustaining proliferation (Ben-David and Amon, 2020; Dhital and Rodriguez-Bravo, 2023). This balance appears critical: more severe disruption of mitotic control can push aneuploidy beyond what cancer cells can tolerate, resulting in cell death (Dominguez-Brauer et al, 2015). Consistent with this, cancer cells are more sensitive than normal cells to prolonged mitotic arrest (Janssen et al, 2009; Vitale et al, 2011), suggesting that factors which sustain tolerable mitotic progression in cancer cells, but are dispensable in normal cells, could represent attractive and selective therapeutic targets.

UBASH3B is the ubiquitin receptor for the mitotic kinase Aurora B and drives its recruitment to microtubules in cancer cells (Krupina et al, 2016). The spatiotemporal regulation of Aurora B localization is central to mitotic progression, kinetochore–microtubule error correction, and SAC function (Krenn and Musacchio, 2015), placing UBASH3B at a critical node of cancer-relevant mitotic control. UBASH3B is overexpressed in aggressive malignancies including triple-negative breast cancer (TNBC) and leukemia, where it promotes tumor growth, invasion, and metastasis (Jin et al, 2018; Goyama et al, 2016; Yao et al, 2021; Mian et al, 2019; Lee et al, 2013). Crucially, mice lacking UBASH3B show no detectable phenotypes (Carpino et al, 2004), raising the possibility that UBASH3B-dependent Aurora B regulation represents a cancer-specific mitotic dependency. Building on these observations and our published work (Krupina et al, 2016, 2018), we hypothesized that pharmacological inhibition of UBASH3B would disrupt Aurora B regulation, interfere with mitotic progression, and selectively reduce cancer cell viability.

To test this hypothesis, we identified UBASHIN, a small-molecule inhibitor that preferentially targets the 2HP domain of UBASH3B. UBASHIN disrupts UBASH3B localization to spindle-associated structures, alters Aurora B localization and activity, and induces prolonged mitotic arrest and cell death, closely phenocopying siRNA-mediated UBASH3B depletion (Krupina et al, 2016, 2018). Cancer cells with high UBASH3B expression show increased sensitivity to UBASHIN, whereas normal cells remain unaffected. This sensitivity is not explained by basal aneuploidy or pre-existing CIN, but is consistent with a context-dependent reliance on UBASH3B-linked mitotic regulation. In vivo, UBASHIN reduces tumor growth in mouse cancer models without causing morphological or behavioral defects in healthy animals. Together, these findings establish UBASH3B as a cancer-specific regulator of mitotic progression and provide proof-of-principle for a therapeutic strategy targeting this vulnerability.

## Results

### The 2HP domain is essential for UBASH3B-mediated centromeric targeting of Aurora B

To target the mitotic function of UBASH3B pharmacologically, we first set out to identify a druggable domain within the protein. As a member of the UBASH3 protein family, UBASH3B features a unique combination of a ubiquitin-associated (UBA) domain, a Src homology-3 (SH3) domain, and a C-terminal histidine phosphatase-like domain homologous to phosphoglycerate mutase (PGM) (Tsygankov, 2020). We previously reported that UBASH3B facilitates centromeric localization of ubiquitylated Aurora B in prometaphase via its N-terminal (NT) part containing the UBA domain (Krupina et al, 2016). While the PGM domain has been widely implicated in cellular signaling and cancer development (Yao et al, 2021; Kowanetz et al, 2004), it is unlikely to play a role in mitotic progression based on available data (Krupina et al, 2016). Interestingly, an uncharacterized region between the UBA and SH3 domains contains a conserved 2-histidine phosphodiesterase (2HP) motif, classifying UBASH3B as a member of the 2H phosphoesterase family (Mazumder et al, 2002). The enzymatic pocket of this motif represents a potential small-molecule binding site.

**Figure 1:**
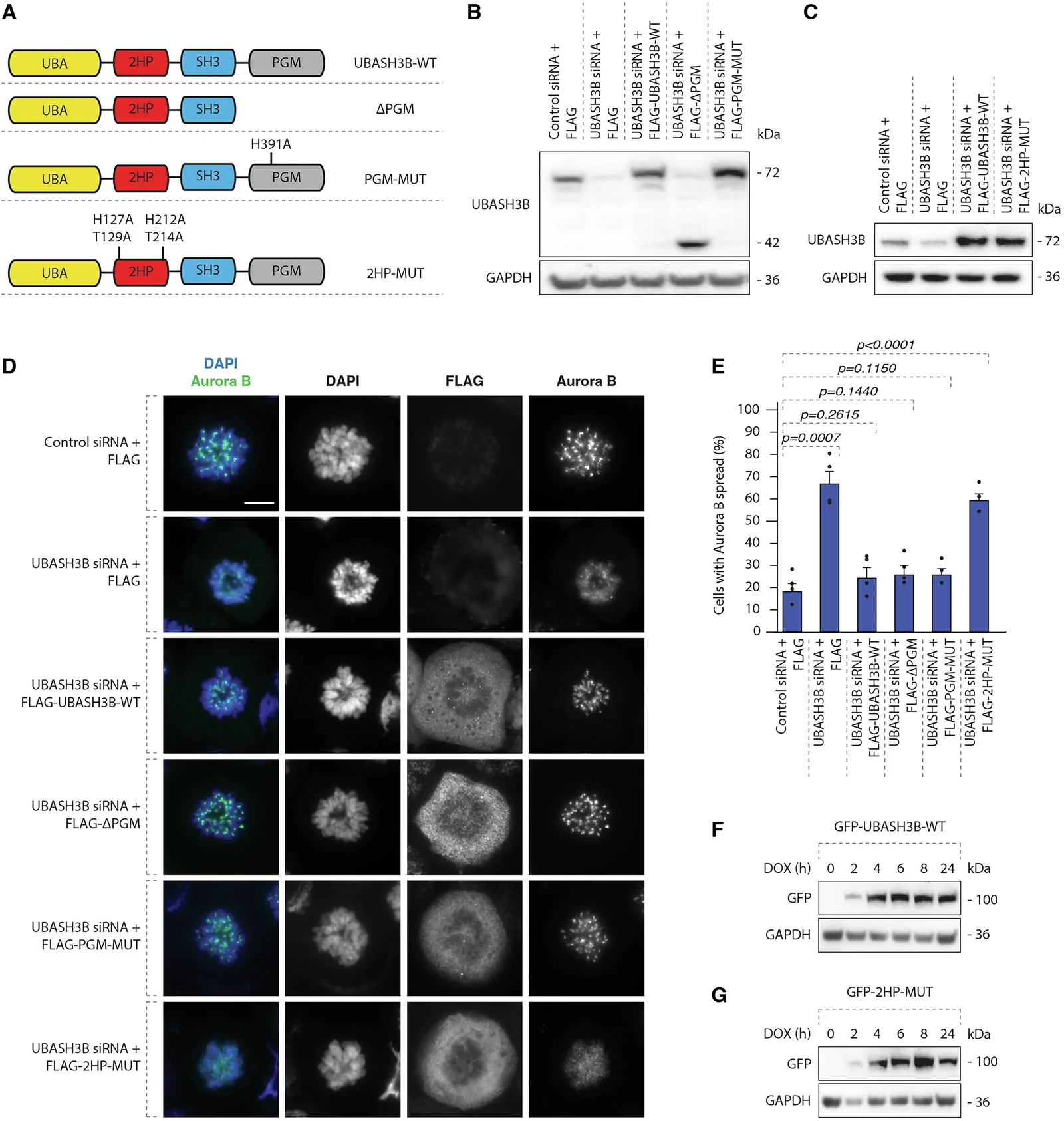
2HP domain is essential for UBASH3B-mediated centromeric targeting of Aurora B. (**A**) Schematic representation of the UBASH3B constructs used in this study. UBASH3B-WT corresponds to the full-length wild-type protein; ΔPGM lacks the C-terminal phosphoglycerate mutase-like domain; PGM-MUT carries the H391A catalytic mutation; and 2HP-MUT carries alanine substitutions in the conserved 2HP catalytic motifs, H127A/T129A and H212A/T214A. UBA ubiquitin-associated domain, 2HP 2-histidine phosphoesterase domain, SH3 Src homology 3 domain, PGM phosphoglycerate mutase-like domain. (**B**, **C**) WB analysis of mitotic HeLa cells showing UBASH3B depletion using siRNA targeting the 3′-UTR and re-expression of the indicated FLAG-tagged UBASH3B constructs. GAPDH was used as a loading control. (**D**) Representative IF images of mitotic HeLa cells stained for Aurora B (green), FLAG and DNA (DAPI, blue). Cells were treated with control siRNA, UBASH3B siRNA, or UBASH3B siRNA followed by re-expression of the indicated FLAG-tagged UBASH3B constructs. Scale bar, 5 µm. (**E**) Quantification of cells displaying Aurora B spreading along chromosome arms. Data are shown as mean ± SEM from four independent experiments; individual data points are shown. *P* values were calculated using two-tailed Welch’s *t* tests. (**F**, **G**) WB of doxycycline-inducible expression of GFP-UBASH3B-WT (**F**) and GFP-2HP-MUT (**G**) expression over a 24 h time course in HeLa cells. Cells were analyzed with antibodies against GFP and GAPDH (loading control). Source data are available online for this figure.

To understand the function of the 2HP domain and to more precisely dissect the contribution of different UBASH3B domains to its mitotic function, we generated three mutant constructs: a truncated wild-type (WT) fragment lacking the C-terminal PGM domain (FLAG-DPGM), a Full-Length (FL) PGM-deficient mutant H391A (FLAG-PGM-MUT), and an FL mutant in the conserved catalytic motifs of the 2HP domain H127A/T129A and H212A/T214A (FLAG-2HP-MUT) designed to disrupt the predicted catalytic histidines and adjacent conserved threonines that contribute to 2HP active-site organization (Fig. 1A). All constructs were used in UBASH3B-depleted cells using RNAi targeting the 3′-UTR, and efficient knockdown and re-expression were confirmed by Western Blot (WB) (Fig. 1B,C).

In agreement with previous findings (Krupina et al, 2016), we confirmed that depletion of UBASH3B disrupted Aurora B localization, causing it to spread along chromosome arms instead of concentrating at centromeres (Fig. 1D,E). Re-expression of either FLAG-UBASH3B-WT, FLAG-ΔPGM, or FLAG-PGM-MUT efficiently rescued centromeric Aurora B localization, confirming that the NT region containing the UBA and SH3 domains is sufficient for this function, and that the PGM domain and its catalytic activity are dispensable. In contrast, expression of FLAG-2HP-MUT failed to restore Aurora B centromeric localization, demonstrating that the 2HP domain contributes to Aurora B targeting during mitosis and prompting us to further investigate its role in mitotic progression.

### The 2HP domain is required for UBASH3B localization and mitotic fidelity

**Figure 2:**
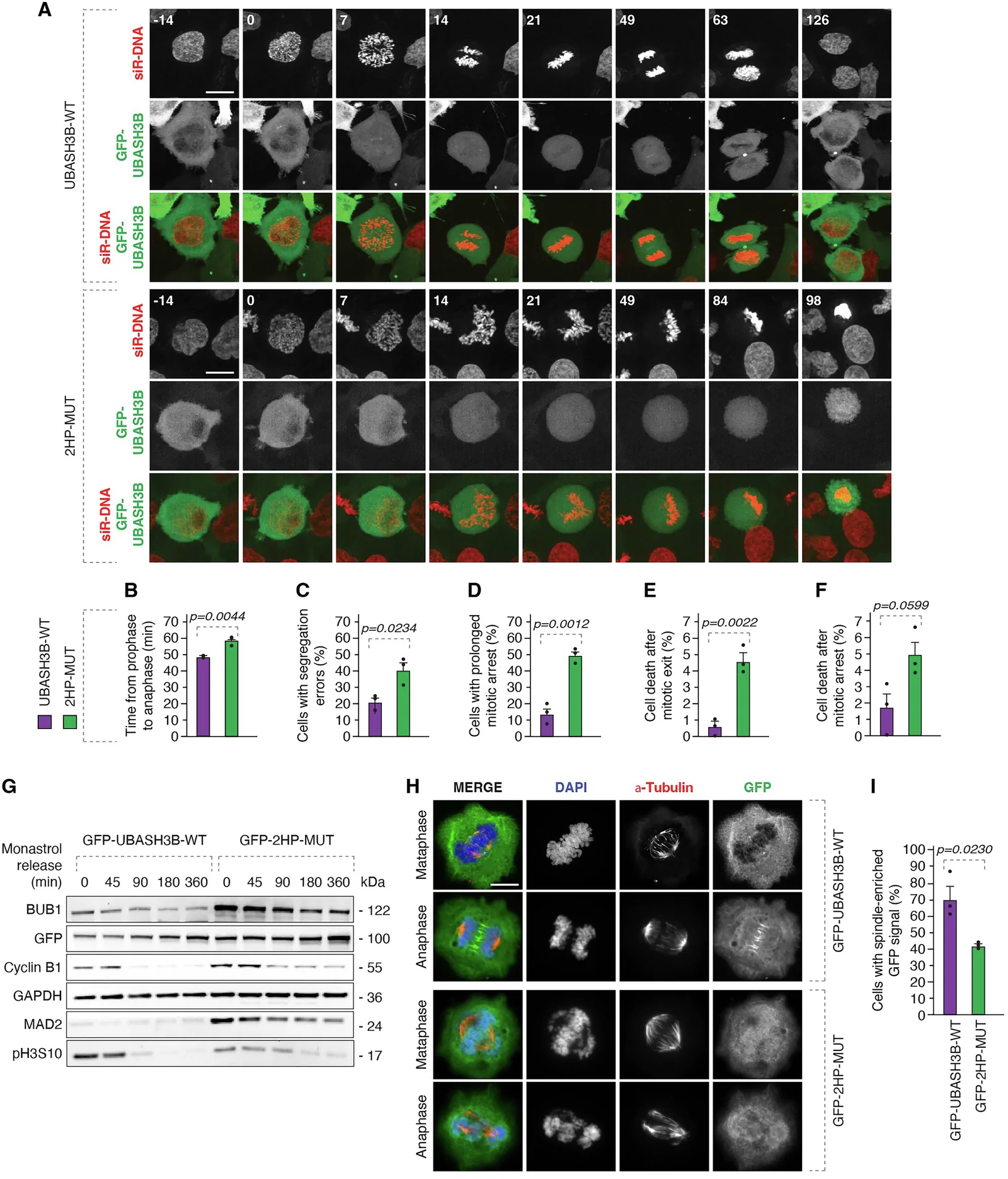
The 2HP domain is required for UBASH3B localization and mitotic progression. (**A**) Live-cell imaging of HeLa cells expressing GFP-UBASH3B-WT or GFP-2HP-MUT. GFP-UBASH3B is shown in green and chromosomes were visualized with SiR-DNA (red). Time-lapse frames show mitotic progression, with timestamps (in minutes) relative to nuclear envelope breakdown (time = 0). Scale bars, 10 µm. (**B**–**F**) Quantification of mitotic phenotypes in GFP-UBASH3B-WT and GFP-2HP-MUT cells: time from prophase to anaphase (**B**), % of cells with chromosome segregation errors (**C**), % of cells undergoing prolonged mitotic arrest (**D**), % of cells dying after mitotic exit (**E**), and % of cells dying during mitosis (**F**). Data are shown as mean ± SEM from three independent experiments; individual data points are shown. *P* values were calculated using two-tailed paired Student’s *t* tests. (**G**) WB of HeLa cells expressing GFP-UBASH3B-WT or GFP-2HP-MUT following release from monastrol-induced prometaphase arrest. Cells were analyzed with antibodies against GFP, Cyclin B1, pH3S10, Bub1, MAD2 and GAPDH (loading control). (**H**) Representative IF images of HeLa cells expressing GFP-UBASH3B-WT or GFP-2HP-MUT showing UBASH3B localization during mitosis in HeLa cells. Cells were stained for α-tubulin (red) and DNA (DAPI, blue). GFP-UBASH3B is shown in green. Scale bar, 5 µm. (**I**) Quantification of cells with spindle-enriched UBASH3B localization. Data are shown as mean ± SEM from three independent experiments; individual data points are shown. *P* values were calculated using two-tailed paired Student’s *t* test. Source data are available online for this figure.

To investigate the contribution of the 2HP domain to mitotic progression, we generated stable, doxycycline-inducible HeLa cell lines expressing GFP-UBASH3B-WT or GFP-2HP-MUT (Fig. 1F,G) and analyzed them by live-cell imaging (Fig. 2A; Movies EV1 and 2). In contrast to GFP-UBASH3B-WT cells, where mitosis proceeded efficiently, with proper chromosome alignment and segregation, GFP-2HP-MUT cells exhibited delayed mitotic progression with increased time spent from prophase to anaphase, frequent chromosome segregation errors, and prolonged mitotic arrest (Fig. 2A–D). These defects were associated with higher rates of mitotic cell death, both during and after mitotic exit (Fig. 2E,F). These phenotypes are consistent with the established importance of mitotic duration, which is normally tightly constrained, as even minute-scale extensions can trigger threshold-dependent surveillance responses (Meitinger et al, 2024; Sparr and Meitinger, 2025; Fulcher et al, 2025). Complementary to these findings, monastrol arrest and release time-course experiments further highlighted the impact of 2HP mutation on mitotic dynamics. WB analysis revealed that the GFP-2HP-MUT cells retained elevated levels of mitotic markers, such as Cyclin B1 and pH3S10, as well as checkpoint-associated proteins, such as Bub1 and MAD2, relative to WT cells (Fig. 2G), consistent with delayed mitotic exit and prolonged mitotic arrest, as observed by live-cell imaging.

To understand how the 2HP domain contributes to UBASH3B mitotic function, we examined the localization of this protein during mitosis. GFP-UBASH3B-WT was enriched at spindle-associated structures including centrosomes and the midzone during metaphase and anaphase, respectively, as shown by colocalization with α-tubulin (Fig. 2H,I), consistent with our earlier observations (Krupina et al, 2016). In contrast, GFP-2HP-MUT exhibited a diffuse cytoplasmic distribution with significantly reduced spindle enrichment, and its expression was associated with the formation of tripolar spindles. This mislocalization caused by the 2HP mutation likely underlies the observed mitotic abnormalities and explains how this domain contributes to proper Aurora B localization. Together, these data suggest that while the UBA domain is responsible for the interaction with the monoubiquitylated Aurora B (Krupina et al, 2016), the 2HP motif contributes to the targeting or retention of the UBASH3B-Aurora B complex at mitotic microtubules, thereby supporting mitotic fidelity.

### Chemical screening identifies UBASHIN as a small-molecule inhibitor of UBASH3B

**Figure EV1:**
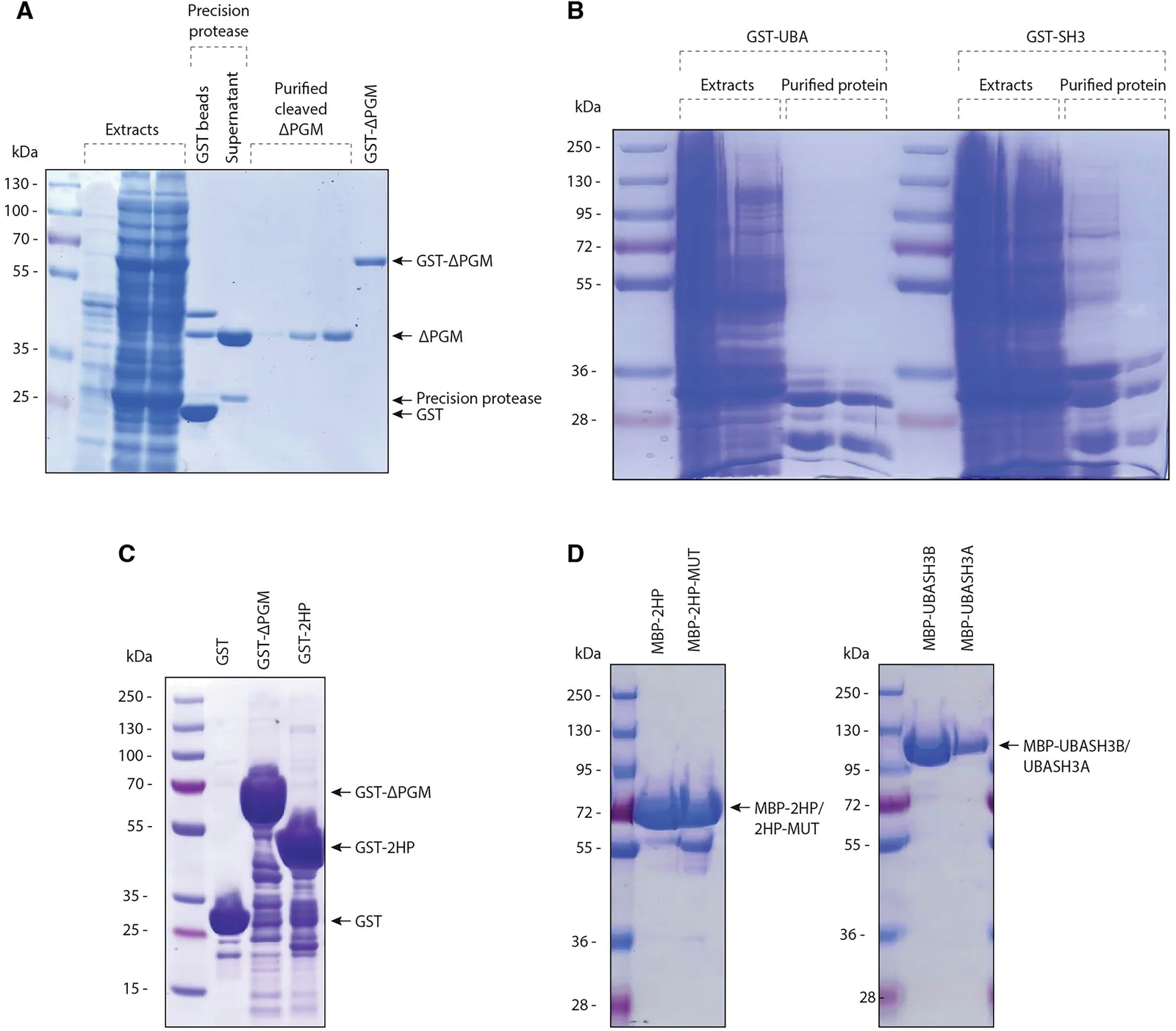
Purification of UBASH3B and UBASH3A protein fragments used for biochemical assays. (**A**) Coomassie-stained SDS-PAGE gel showing purification of GST-UBASH3B-ΔPGM from bacterial extracts and cleavage to release the UBASH3B-ΔPGM fragment used for FP assays. Bands corresponding to GST-UBASH3B-ΔPGM, cleaved UBASH3B-ΔPGM, GST and precision protease are indicated. (**B**) Coomassie-stained SDS-PAGE gel showing bacterial extracts and purified GST-UBA and GST-SH3 fragments used for FP assays. (**C**) Coomassie-stained SDS-PAGE gel showing purified GST-UBASH3B-ΔPGM and GST-2HP fragments used for FP assays. (**D**) Coomassie-stained SDS-PAGE gel showing purified MBP-tagged UBASH3B 2HP, UBASH3B 2HP-MUT, UBASH3B-WT and UBASH3A-WT proteins used for SPR analysis.

**Figure 3:**
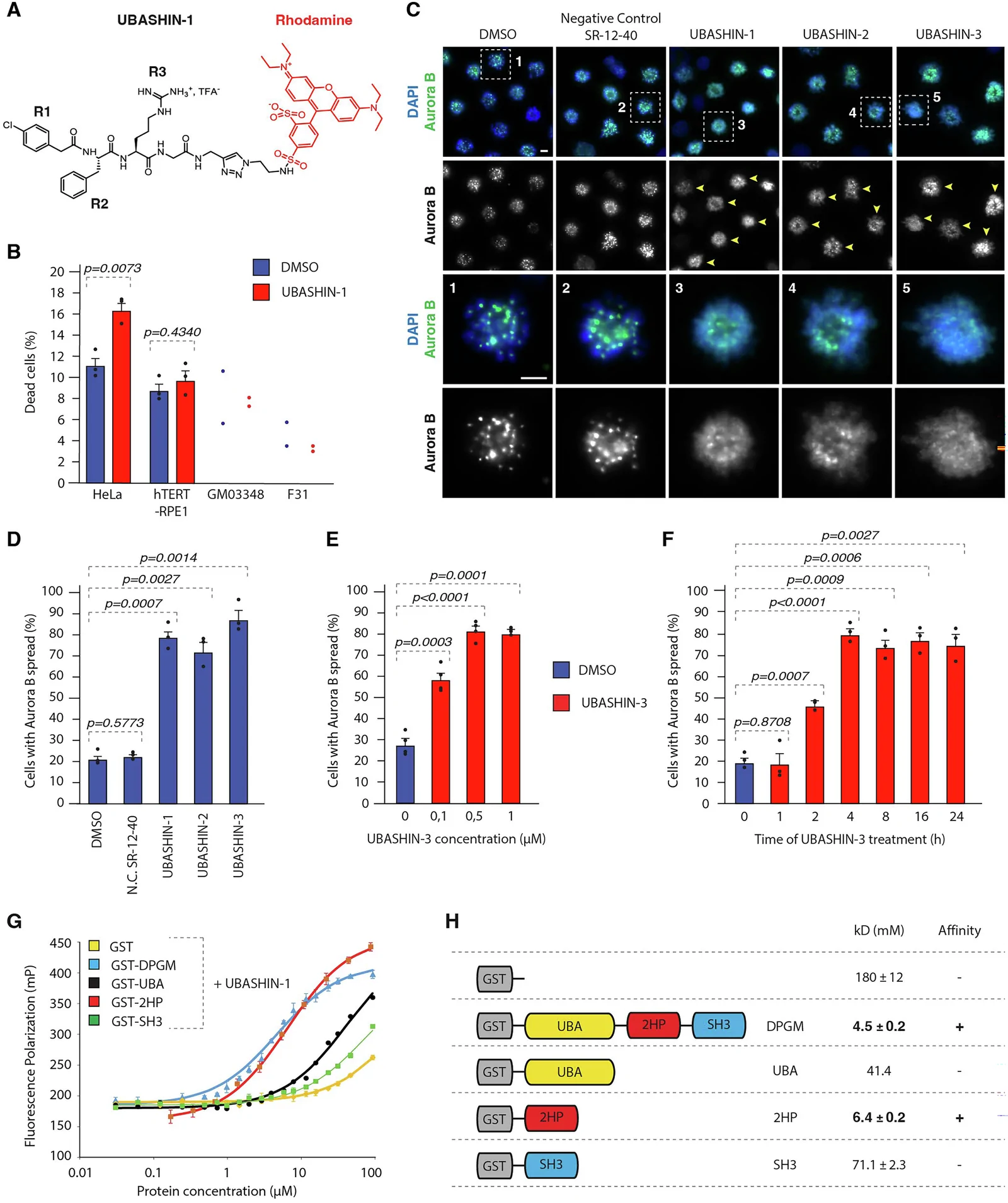
Chemical screening identifies UBASHIN as a small-molecule inhibitor of UBASH3B. (**A**) Chemical structure of UBASHIN-1. (**B**) Flow cytometry-based analysis of cell death in HeLa, hTERT RPE-1, GM03458 and F31 cells treated with DMSO or UBASHIN-1. Data are shown as mean ± SEM from three independent experiments for HeLa and hTERT RPE-1 cells; individual data points are shown. *P* values were calculated using two-tailed Welch’s *t* tests. Individual values from two independent experiments are shown for GM03458 and F31 cells. Data are presented descriptively, without error bars or inferential statistical testing. (**C**) Representative IF images of mitotic HeLa cells treated with DMSO, negative control compound SR-12-40, UBASHIN-1, UBASHIN-2 or UBASHIN-3. Cells were stained for Aurora B (green) and DNA (DAPI, blue). Arrowheads indicate Aurora B spreading along chromosome arms. Scale bars, 15 µm and 10 µm for large-field images and 5 µm for cropped regions. (**D**) Quantification of cells displaying Aurora B spreading along chromosome arms under the indicated treatment conditions. Data are shown as mean ± SEM from three independent experiments; individual data points are shown. *P* values were calculated using two-tailed Welch’s *t* tests. (**E**) Dose-dependent effect of UBASHIN-3 on Aurora B localization in mitotic HeLa cells. Data are shown as mean ± SEM from four independent experiments; individual data points are shown. *P* values were calculated using two-tailed Welch’s *t* tests. (**F**) Time-course analysis of UBASHIN-3-induced Aurora B spreading along chromosome arms in mitotic HeLa cells. Data are shown as mean ± SEM from three independent experiments; individual data points are shown. *P* values were calculated using two-tailed Welch’s *t* tests. (**G**) Fluorescence Polarization (FP) binding assay showing UBASHIN-1 binding to purified GST, GST-UBASH3B-ΔPGM, GST-UBA, GST-2HP and GST-SH3 fragments. Data are shown as mean ± SD from three independent experiments. (**H**) Summary of apparent dissociation constants (kD) derived from the FP assay in (**F**). Values are reported as apparent kD estimates and were used to compare relative binding between UBASH3B fragments. Relative binding is indicated as “+” when binding was observed and “–” when no convincing binding was detected under the tested conditions. Source data are available online for this figure.

The discovery of the 2HP domain’s important role in UBASH3B-mediated mitotic regulation (Krupina et al, 2016, 2018), combined with the overexpression of UBASH3B in aggressive cancers (Goyama et al, 2016; Lee et al, 2013), prompted us to conduct chemical screening aiming to identify small-molecule inhibitors targeting the N-terminal part of UBASH3B protein. Bacterially derived and purified UBASH3B-DPGM fragments containing UBA, 2HP and SH3 domains (EV1A) were used for screening a 3000-compound library containing small rhodamine-labeled molecules with unique chemical structures by fluorescence polarization (FP). The FP assays identified a peptidic compound featuring key residues (R1: chlorobenzene, R2: benzene, R3: guanidine) and a lissamine rhodamine dye for FP detection (Fig. 3A; Appendix Fig. S1A) that bound preferentially to the UBASH3B-DPGM protein fragment (Fig. EV1A). The original compound identified from these assays (compound 1, FD-E09), along with its derivatives (compounds 2–5) designed and used throughout this study, are collectively referred to as UBASHIN (for UBASH3B-INhibitor) and numbered UBASHIN-1 through UBASHIN-5 accordingly (Appendix Fig. S1A).

Subsequent functional analysis revealed that treatment with UBASHIN-1 induced cell death in HeLa cancer cells relative to DMSO-treated control cells, whereas survival of non-cancerous HTERT RPE-1 cells and primary human fibroblasts (GM03348 and F31) was not detectably affected (Fig. 3B). This differential response suggests that UBASHIN-1 may exploit cancer-associated vulnerabilities such as dysregulated mitotic pathways.

To evaluate whether treatment with UBASHIN-1 phenocopies reported Aurora B localization defects observed upon UBASH3B depletion (Krupina et al, 2016) and if these phenotypes are due to the unique chemical structure of the inhibitor and not the rhodamine moiety, we synthesized the non-fluorescent derivatives UBASHIN-2 and UBASHIN-3, along with the negative control (N.C.) compound SR-12-40, which showed no binding on the chemical screen (Appendix Fig. S1A). UBASHIN-3 substituted the rhodamine dye with a hydrophobic adamantyl to enhance cell permeability, while UBASHIN-2 lacked any substituent residue. In mitotically arrested HeLa cells, all three UBASHIN compounds induced significant Aurora B spreading to the chromosome arms compared to DMSO-treated control cells. In contrast, N.C. SR-12-40 had no effect, confirming the specificity of the UBASHIN chemical structure to the UBASH3B-Aurora B pathway (Fig. 3C,D). Among the three compounds, UBASHIN-3 appeared as the most effective at disrupting Aurora B localization (Fig. 3D) and was further characterized for dose- and time-dependent effects. At 1 μM concentration, UBASHIN-3 robustly induced Aurora B mislocalization to the chromosome arms, with similar effects at 0.5 μM and less pronounced but significant effects at 0.1 μM, relative to DMSO (Fig. 3E). Time-course analysis revealed Aurora B mislocalization after 4 h of UBASHIN-3 treatment, with some defects observed within 2 h (Fig. 3F). These findings demonstrate that treatment with UBASHIN-3 mimics the Aurora B localization phenotype observed upon downregulation of UBASH3B by siRNA (Krupina et al, 2016), offering a valuable and rapid alternative for studying Aurora B dynamics during mitosis.

### UBASHIN binds to the 2HP domain of UBASH3B

To further investigate the domain within the UBASH3B-DPGM protein to which UBASHIN-1 binds, we purified GST-tagged fragments corresponding to isolated UBA, 2HP and SH3 domains, respectively (Fig. EV1B,C) and analyzed them in FP experiments. Interestingly, UBASHIN-1 exhibits higher binding affinity for the isolated 2HP domain compared to other domains and the GST control (Fig. 3G,H). UBASHIN-1 displays equally strong binding to the isolated 2HP domain compared to the entire UBASH3B-DPGM fragment (Fig. 3G,H), suggesting that the inhibitor may fit well the 2HP pocket of UBASH3B.

**Figure 4:**
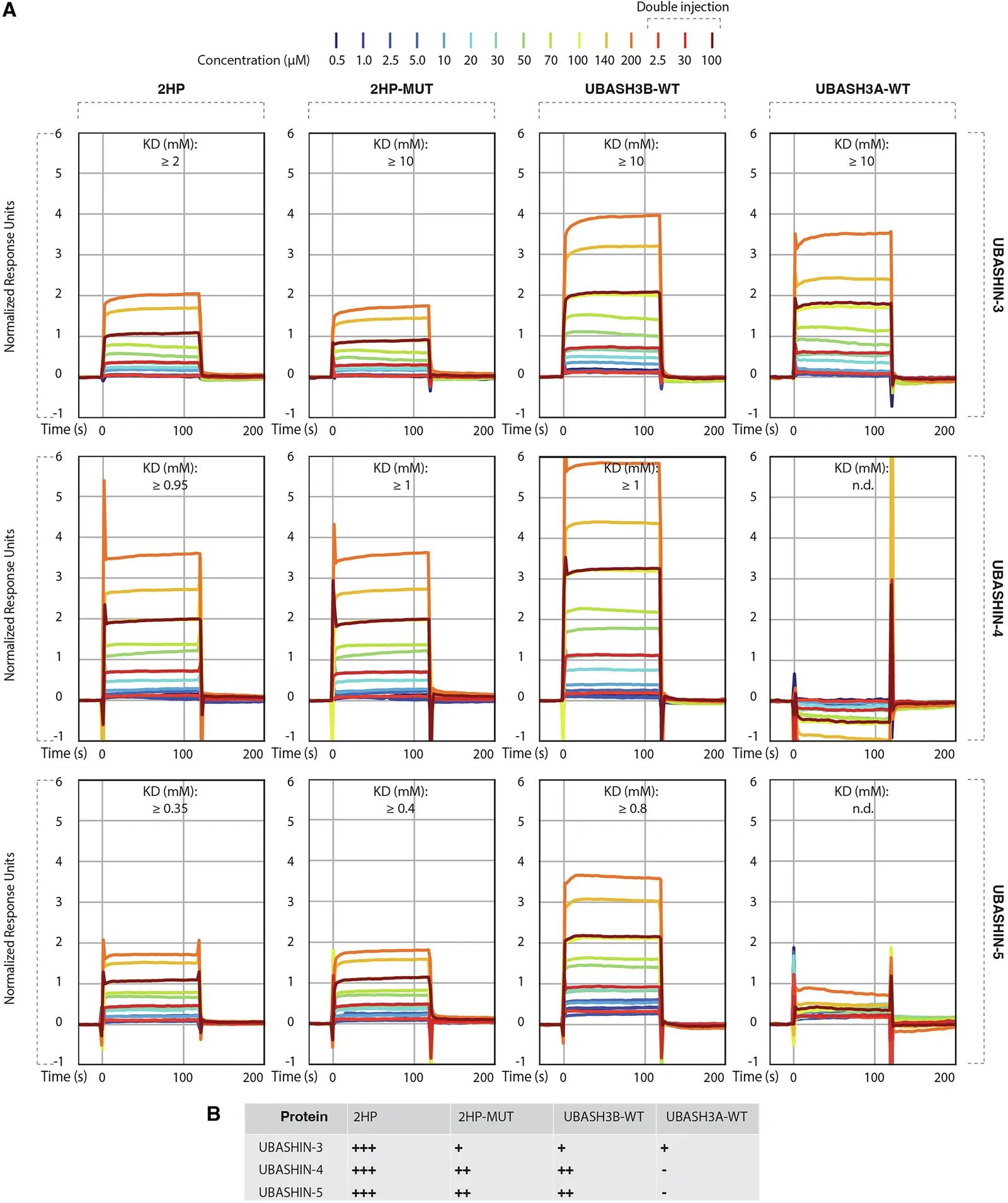
UBASHIN derivatives preferentially engage the UBASH3B 2HP domain. (**A**) Surface plasmon resonance (SPR) sensorgrams showing binding of UBASHIN-3, UBASHIN-4 and UBASHIN-5 to purified MBP-tagged UBASH3B 2HP, UBASH3B 2HP-MUT, UBASH3B-WT and UBASH3A-WT proteins. Increasing compound concentrations were injected as indicated. The 2.5, 30 and 100 µM concentrations were injected in duplicated in order to assess the data reproducibility. Reference-subtracted and normalized responses are shown. Apparent K_D_ values are indicated for each protein-compound pair and should be interpreted as relative affinity estimates, since binding responses did not always reach a clear saturation plateau. Reference-subtracted and normalized responses are shown. Apparent K_D_ values are indicated for each protein-compound pair and should be interpreted as relative affinity estimates, since binding responses did not always reach a clear saturation plateau. n.d. =  non-determined. (**B**) Summary of the relative SPR binding responses from (**A**). Relative binding is indicated as +++, ++, + or – based on the consistency and strength of the sensorgram responses under the tested conditions. +++, most convincing relative SPR response; ++, clear but weaker or less well-resolved SPR response; +, weak SPR response or possible cross-reactivity; –, no convincing interaction detected. Source data are available online for this figure.

**Figure EV2:**
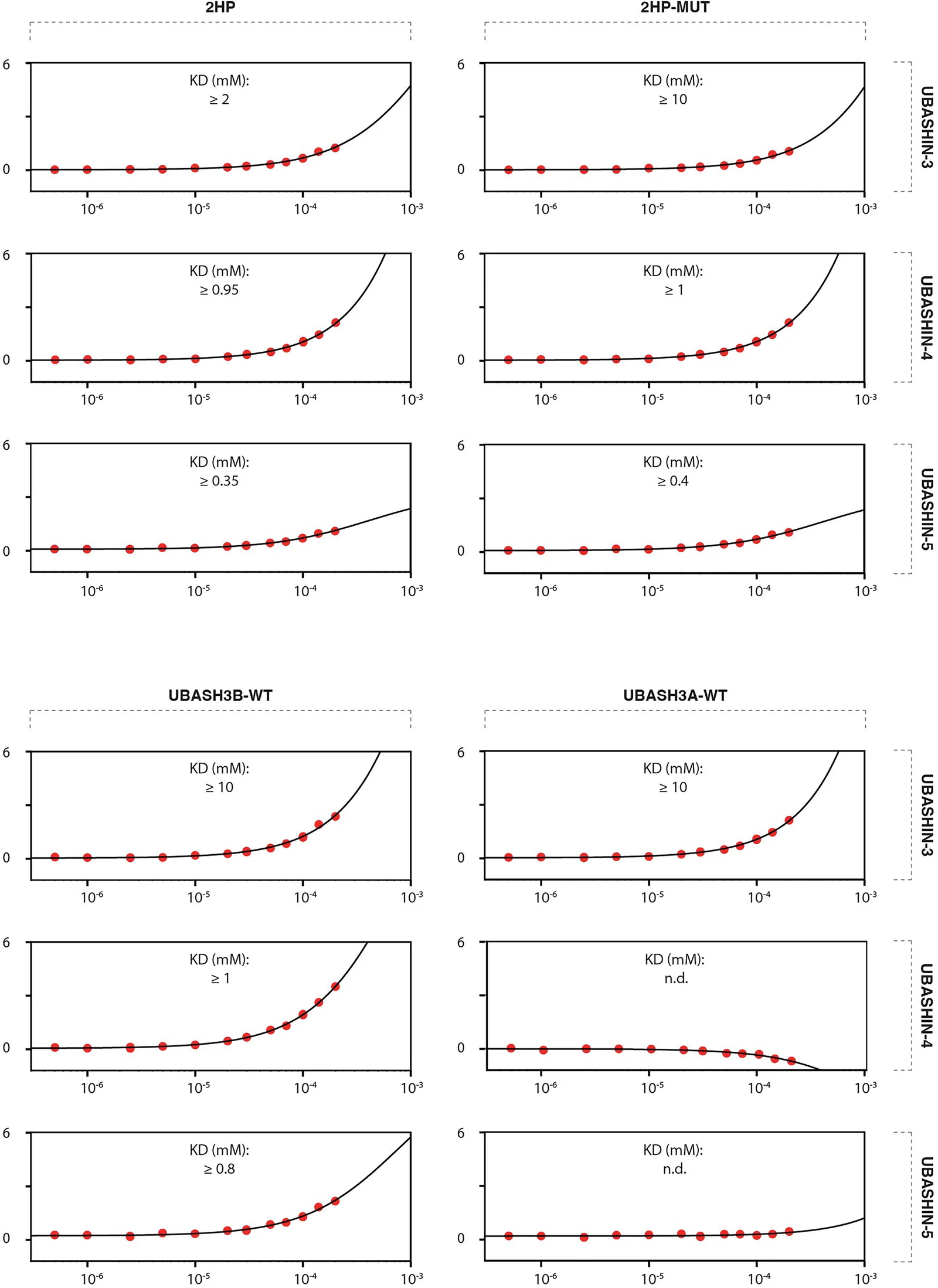
Steady-state SPR binding analysis of UBASHIN derivatives. Steady-state binding curves derived from surface plasmon resonance (SPR) analysis of UBASHIN-3, UBASHIN-4 and UBASHIN-5 binding to purified MBP-tagged UBASH3B 2HP, UBASH3B 2HP-MUT, UBASH3B-WT and UBASH3A-WT proteins. Apparent K_D_ values are indicated for each protein–compound pair. Because several curves did not reach a clear saturation plateau within the tested concentration range, Kd values should be interpreted as apparent affinity estimates used for relative comparison between constructs, rather than precise equilibrium constants. n.d. non-determined values.

To further optimize the compound for subsequent in vitro and in vivo studies, we synthesized the additional derivatives UBASHIN-4 and UBASHIN-5 (Appendix Fig. S1A), which displayed enhanced plasma stability (Appendix Fig. S1B). To validate the affinity of the several derivatives to UBASH3B, we conducted Surface Plasmon Resonance (SPR) using purified maltose binding protein (MBP)-tagged 2HP and 2HP-MUT isolated domains, as well as MBP-UBASH3B FL and the closely homologous protein MBP-UBASH3A FL (Fig. EV1D). A similar binding trend was observed for all compounds tested (UBASHIN-3, UBASHIN-4 and UBASHIN-5) with the 2HP domain (Fig. 4A,B). Interestingly, the 2HP catalytically-inactive mutant domain can still bind to all compounds tested, displaying only a slight reduction in binding relative to the 2HP WT (Fig. 4A,B). This result indicates that the structural integrity of the entire 2HP domain, but not solely its catalytically active residues, contributes to the interaction with UBASHIN. Moreover, UBASH3B FL binds to all compounds tested with lower affinity relative to the isolated 2HP domain (Fig. 4A,B), suggesting that the 2HP domain is the primary site of interaction within UBASH3B. Finally, UBASH3A FL displayed only weak and compound-dependent interaction, consistent with partial paralog cross-reactivity rather than a shared binding profile across the series (Fig. 4A,B). Steady-state analysis was also performed as the signals reached plateaus at each individual concentration (Fig. EV2). Although signal saturation was not achieved at high compound concentrations, precluding precise affinity constant determination, the derived K_D_ values should be considered as apparent affinity estimates, providing a useful comparison between constructs rather than exact equilibrium constants. These estimates confirmed our preliminary conclusions drawn from the kinetic data and altogether indicate that the UBASHIN series preferentially engages the 2HP domain of UBASH3B.

### Inhibition of UBASH3B by UBASHIN leads to prolonged mitotic arrest

**Figure 5:**
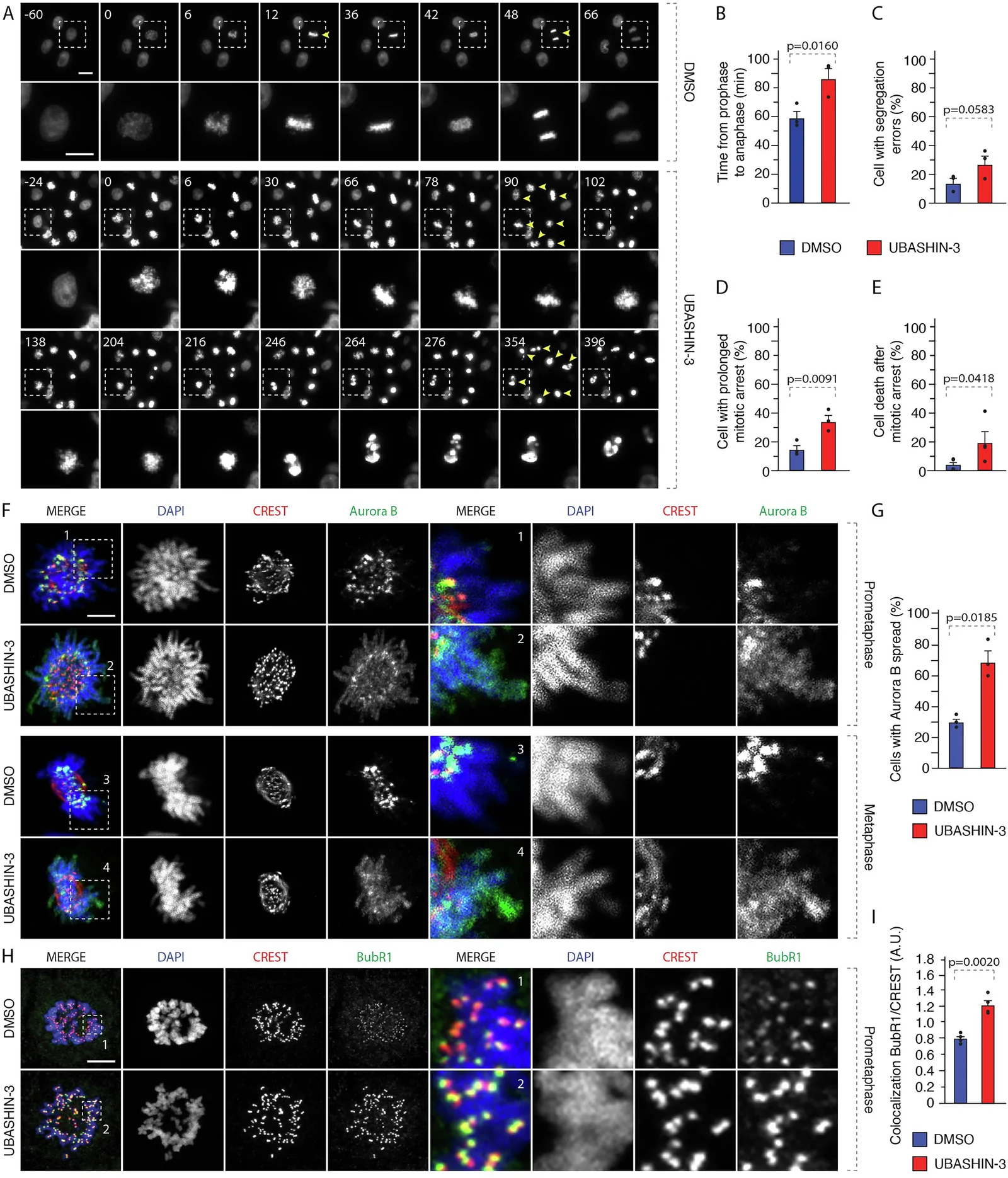
Inhibition of UBASH3B by UBASHIN leads to prolonged mitotic arrest. (**A**) Live-cell imaging of HeLa cells stably expressing H2B-mCherry treated with DMSO or UBASHIN-3. Large-field images show the tracked cell population and enlarged cropped regions highlight the representative cell followed through mitosis. Time-lapse frames show mitotic progression, with timestamps in minutes relative to nuclear envelope breakdown (time = 0). Scale bars, 10 µm for large-field images and 5 µm for cropped regions. (**B**–**E**) Quantification of mitotic phenotypes in DMSO- and UBASHIN-3-treated cells: time from prophase to anaphase (**B**), % of cells with chromosome segregation errors (**C**), % of cells undergoing prolonged mitotic arrest (**D**), and % of cells undergoing mitotic cell death (**E**). Data are shown as mean ± SEM from three independent experiments; individual data points are shown. *P* values were calculated using one-tailed unpaired Student’s *t* tests. (**F**) Representative IF images of prometaphase and metaphase HeLa cells treated with DMSO or UBASHIN-3 and stained for Aurora B (green), CREST (red) and DNA (DAPI, blue). Zoomed regions highlight Aurora B localization at chromosomes. Scale bar, 5 µm. (**G**) Quantification of cells displaying Aurora B spreading along chromosome arms. Data are shown as mean ± SEM from three independent experiments; individual data points are shown. *P* values were calculated using a one-tailed unpaired Student’s *t* test. (**H**) Representative IF images of prometaphase HeLa cells treated with DMSO or UBASHIN-3 and stained for BubR1 (green), CREST (red) and DNA (DAPI, blue). Zoomed regions highlight kinetochore-associated BubR1 signal. Scale bar, 5 µm. (**I**) Quantification of BubR1 intensity normalized to CREST at kinetochores. Data are shown as mean ± SEM from four independent experiments; individual data points are shown. *P* values were calculated using a two-tailed Welch’s *t* test. A.U. arbitrary units. Source data are available online for this figure.

Having confirmed the binding of UBASHIN derivatives to UBASH3B and their preferential engagement of the 2HP domain, we next sought to understand the consequences of UBASH3B inhibition on mitotic progression. HeLa cells stably expressing H2B-mCherry as a chromosomal marker were treated with UBASHIN-3 and analyzed by live-cell imaging (Fig. 5A; Movies EV3 and 4). UBASHIN-3 treatment significantly extended the average duration from prophase to anaphase to 85 min relative to 58 min in the DMSO-treated controls (Fig. 5B). Furthermore, UBASHIN-3-treated cells displayed chromosome segregation defects and prolonged mitotic arrest, ultimately leading to mitotic cell death (Fig. 5C–E), highly reminiscent of the effects of UBASH3B downregulation by siRNA (Krupina et al, 2016).

**Figure EV3:**
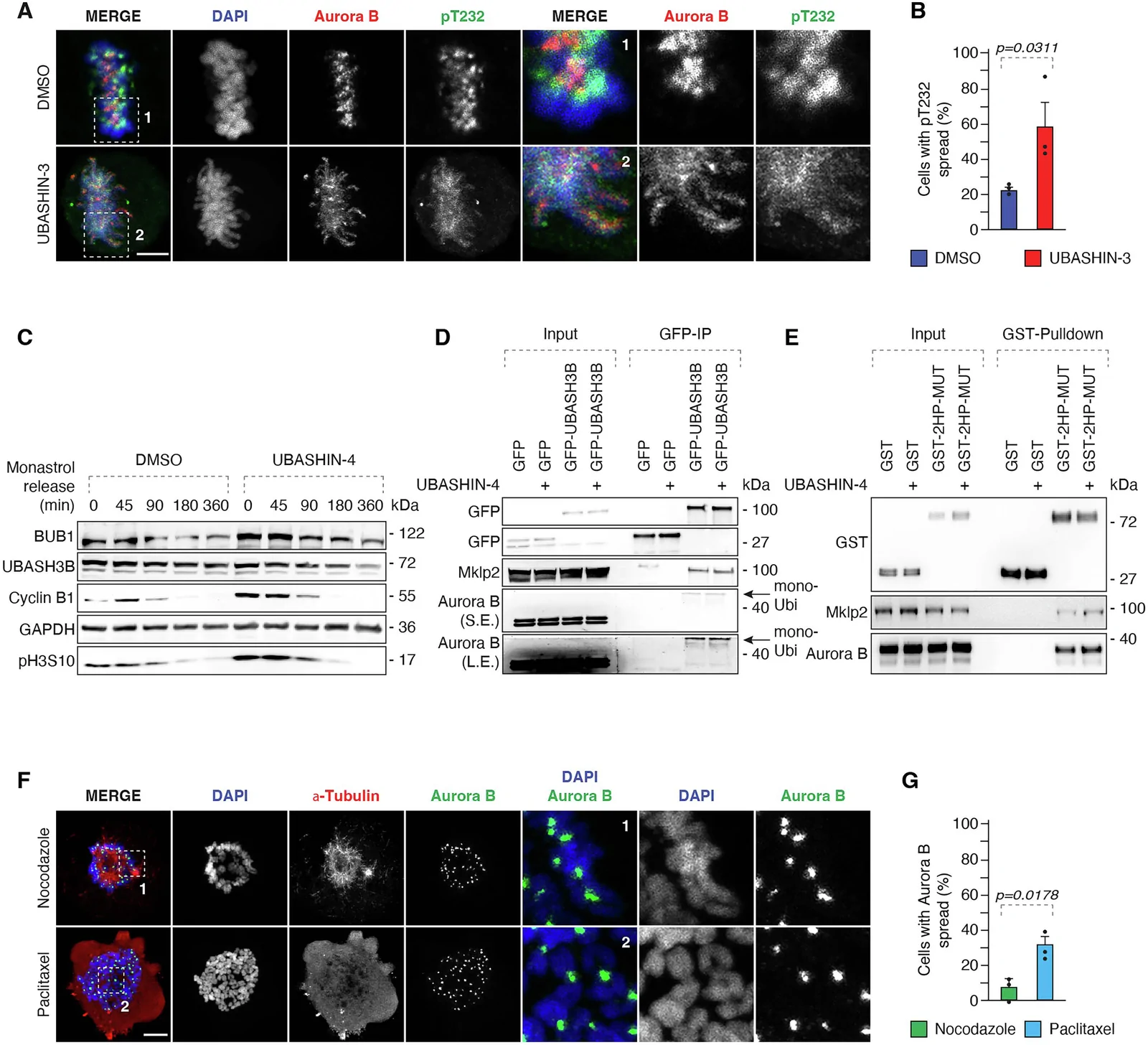
UBASHIN alters Aurora B localization and activity without disrupting UBASH3B-Aurora B/Mklp2 interactions. (**A**) Representative IF images of MDA-MB-231 cells treated with DMSO or UBASHIN-3 and stained for Aurora B (red), phosphorylated Aurora B at T232 (green) and DNA (DAPI, blue). Zoomed regions highlight Aurora B and pT232 localization on mitotic chromosomes. Scale bar, 5 µm. (**B**) Quantification of cells displaying phosphorylated Aurora B T232 signal spreading along chromosome arms. Data are shown as mean ± SEM from three independent experiments; individual data points are shown. *P* values were calculated using a one-tailed unpaired Student’s *t* test. (**C**) WB analysis of HeLa cells treated with DMSO or UBASHIN-3 following release from monastrol-induced prometaphase arrest. Cells were analyzed with antibodies against Bub1, UBASH3B, Cyclin B1, pH3S10 and GAPDH (loading control). (**D**) GFP immunoprecipitation analysis of UBASH3B interaction with Aurora B and Mklp2 in mitotically synchronized HeLa cells treated with DMSO or UBASHIN-4. (**E**) In vitro binding analysis of Aurora B and Mklp2 interaction with GST or GST-2HP-MUT in the presence or absence of UBASHIN-4. (**F**) Representative IF images of HeLa cells treated with Nocodazole or Paclitaxel and stained for Aurora B (green), α-tubulin (red) and DNA (DAPI, blue). Zoomed regions highlight Aurora B localization. Scale bar, 7.5 µm. (**G**) Quantification of cells displaying Aurora B spreading along chromosome arms after Nocodazole or Paclitaxel treatment. Data are shown as mean ± SEM from three independent experiments; individual data points are shown. *P* values were calculated using two-tailed Welch’s *t* tests.

To determine whether these defects are associated with the reported role of UBASH3B on Aurora B dynamics (Krupina et al, 2016), we analyzed the localization of this mitotic kinase by immunofluorescence microscopy. UBASHIN-3 disrupted the localization of Aurora B to centromeres, leading to its spreading along chromosome arms compared to DMSO-treated prometaphase and metaphase cells (Fig. 5F,G). Moreover, Aurora B activity, measured as the ratio of pT232-phosphorylated Aurora B to total Aurora B on chromosomes of metaphase cells, was markedly reduced upon UBASHIN-3 treatment, further confirming the compound’s effect on localized activity of Aurora B (Fig. EV3A,B). These findings demonstrate that pharmacological inhibition of UBASH3B disrupts the fidelity of mitotic progression, closely mimicking the phenotypes observed upon UBASH3B depletion (Krupina et al, 2016) and mutation of its 2HP domain (Fig. 2).

Indeed, published data demonstrate that downregulation of UBASH3B upregulates SAC signaling, resulting in mitotic arrest, and cell death (Krupina et al, 2016, 2018), raising a possibility that UBASHIN treatment may affect checkpoint-associated responses. Interestingly, immunofluorescence analysis of the SAC serine/threonine-protein kinase BUB1 beta (BubR1) revealed that UBASHIN-3 treatment significantly elevated the intensity of BubR1 on individual kinetochores, as indicated by the relative BubR1/CREST ratio, compared with that in DMSO-treated cells, consistent with sustained mitotic checkpoint engagement (Fig. 5H,I). WB analysis of cells released from monastrol-induced prometaphase arrest confirmed moderate increase in levels of the mitotic markers Cyclin B1 and phosphorylated histone H3 (pH3S10), while UBASH3B protein levels remained largely unaffected during the time-course experiment (Fig. EV3C), supporting delayed mitotic exit. Taken together, pharmacological inhibition of UBASH3B leads to Aurora B mislocalization, retention of SAC components and mitotic arrest, defects that closely resemble those observed upon downregulation of this protein in human cells (Krupina et al, 2016, 2018).

### Inhibition of UBASH3B disrupts its localization to the mitotic spindle

Next, we sought to understand the mechanism by which UBASHIN inhibits UBASH3B during mitosis. We first investigated the effect of UBASHIN on the interaction of UBASH3B with Aurora B and the microtubule motor protein Mklp2, which is involved in Aurora B localization to the anaphase spindle (Gruneberg et al, 2004). Co-immunoprecipitation (co-IP) assays of GFP-tagged UBASH3B in mitotically synchronized cells revealed no significant changes in binding to Mklp2 or modified, presumably monoubiquitylated form of Aurora B (Krupina et al, 2016) upon UBASHIN-4 treatment (Fig. EV3D), suggesting that UBASHIN does not interfere with the recognition of the Aurora B/Mklp2 complex by UBASH3B. To further assess whether UBASHIN alters UBASH3B interactions through its 2HP-dependent mitotic function, we examined the 2HP-mutant protein in vitro. Using purified GST-tagged 2HP-MUT UBASH3B, we found that UBASHIN-4 did not affect binding to Aurora B or Mklp2 (Fig. EV3E), consistent with the co-IP data (Fig. EV3D) and suggesting that the inhibitor does not directly disrupt these interactions.

**Figure 6:**
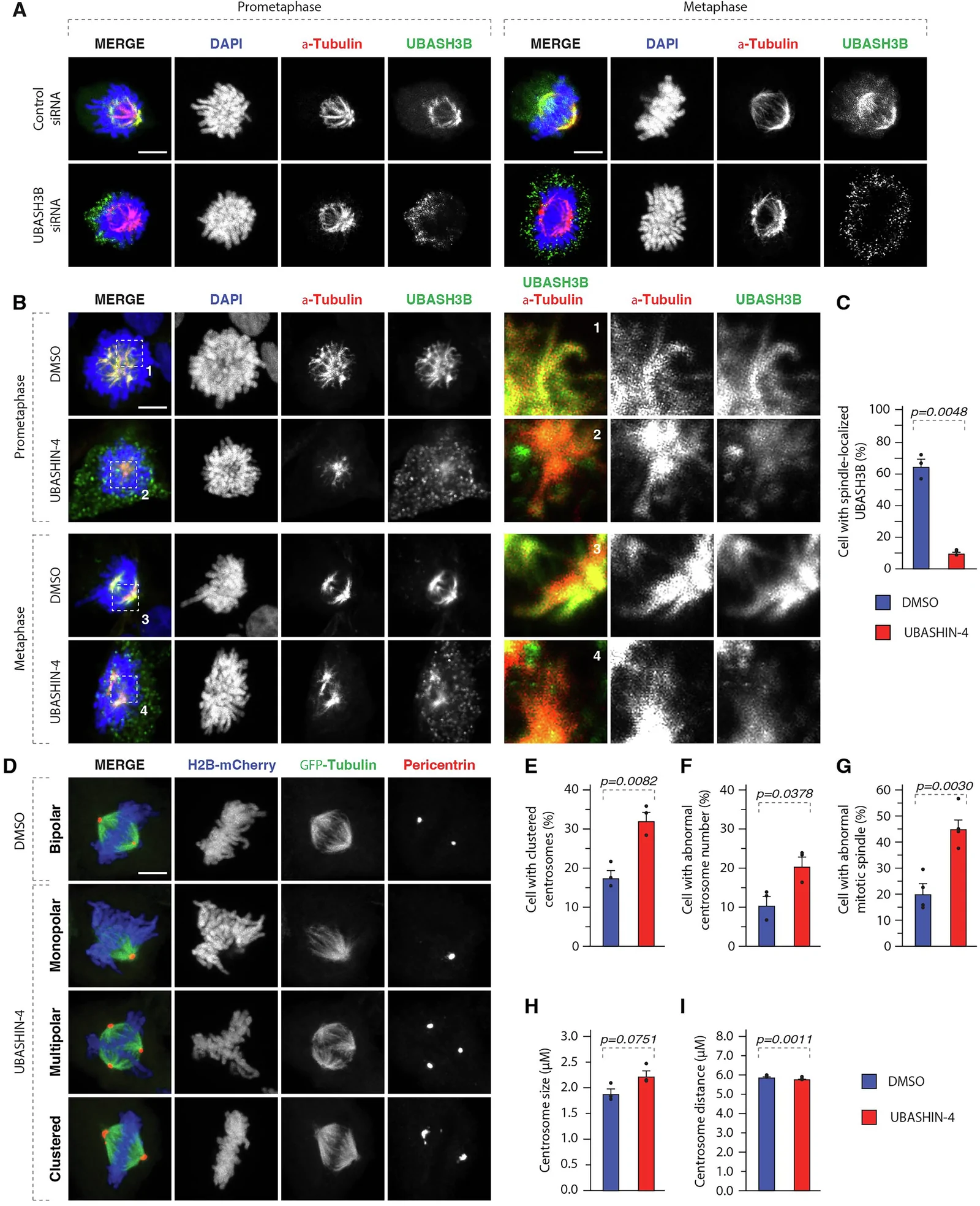
Inhibition of UBASH3B disrupts its localization to the mitotic spindle. (**A**) Representative IF images of MDA-MB-231 cells treated with control siRNA or UBASH3B siRNA, showing endogenous UBASH3B localization during prometaphase and metaphase. Cells were stained for α-Tubulin (red), UBASH3B (green) and DNA (DAPI, blue). Scale bars, 5 µm. (**B**) Representative IF images of MDA-MB-231 cells treated with DMSO or UBASHIN-4, showing endogenous UBASH3B localization during prometaphase and metaphase. Cells were stained for α-tubulin (red), UBASH3B (green) and DNA (DAPI, blue). Zoomed regions highlight UBASH3B localization relative to spindle microtubules. Scale bar, 5 µm. (**C**) Quantification of cells with spindle-enriched UBASH3B localization under the indicated treatment conditions. Data are shown as mean ± SEM from three independent experiments; individual data points are shown. *P* values were calculated using two-tailed Welch’s *t* tests. (**D**) Representative IF images of HeLa cells stably expressing H2B-mCherry and GFP-Tubulin, treated with DMSO or UBASHIN-4. Cells were stained for pericentrin (red) to visualize centrosomes. Examples of bipolar, monopolar, multipolar and clustered centrosome/spindle phenotypes are shown. Scale bar, 5 µm. (**E**–**I**) Quantification of spindle and centrosome phenotypes in DMSO- and UBASHIN-4-treated cells: % of cells with clustered centrosomes (**E**), % of cells with abnormal centrosome number (**F**), % of cells with abnormal spindle architecture (**G**), centrosome size (**H**), and centrosome distance in bipolar spindles (**I**). Data are shown as mean ± SEM from three or four independent experiments, as indicated by individual data points. *P* values were calculated using two-tailed Welch’s *t* tests. Source data are available online for this figure.

We therefore examined the effect of UBASHIN on the subcellular localization of endogenous UBASH3B, which interacts with microtubules to facilitate Aurora B relocalization from centromeres to the mitotic spindle prior to anaphase (Krupina et al, 2016). To validate the specificity of the antibody used for endogenous UBASH3B staining, we transfected MDA-MB-231 cells with control or UBASH3B-targeting siRNA and examined UBASH3B localization by immunofluorescence during prometaphase and metaphase. In control cells, endogenous UBASH3B showed a clear enrichment at the mitotic spindle, where it colocalized with α-tubulin (Fig. 6A). In contrast, UBASH3B depletion markedly reduced this spindle-associated signal, leaving only diffuse cytoplasmic background staining (Fig. 6A), thereby confirming that the observed spindle localization reflects specific detection of endogenous UBASH3B.

Having confirmed the specificity of the UBASH3B antibody and its spindle-associated localization, we next investigated whether UBASHIN affects the mitotic distribution of endogenous UBASH3B. Indeed, UBASHIN-4 treatment caused a diffuse cytoplasmic distribution of UBASH3B, with significantly reduced spindle enrichment compared to DMSO-treated cells during prometaphase and metaphase (Fig. 6B,C). This aberrant UBASH3B localization closely mimicked the phenotypes observed upon mutation of its 2HP domain (Fig. 2H,I), further underscoring the importance of this domain for proper mitotic targeting of UBASH3B. Given the disrupted localization of UBASH3B, we assessed the effect of UBASHIN on spindle integrity. Immunofluorescence analysis revealed aberrant spindle architecture in UBASHIN-4 treated cells, including the presence of multipolar and monopolar spindles, clustered centrosomes, and centrosomes of increased size, while centrosome distance in bipolar spindles was decreased (Fig. 6D–I).

The observed defects in spindle architecture upon UBASHIN treatment led us to investigate whether Aurora B mislocalization (Figs. 1, 3, and 5) might reflect a general perturbation of microtubule dynamics. To address this, we treated HeLa cells with Nocodazole and Paclitaxel, two spindle poisons that, as expected, disrupted spindle organization (Fig. EV3F,G) but did not induce Aurora B relocalization to chromosome arms, consistent with published literature (Kelly et al, 2007; Wang et al, 2012). Collectively, these results indicate that UBASHIN disrupts UBASH3B localization to the mitotic spindle, leading to defects in spindle architecture and altered Aurora B localization in a manner distinct from general spindle perturbation, which together may contribute to SAC-associated mitotic arrest.

### Sensitivity of cancer cells to UBASH3B inhibition

**Figure 7:**
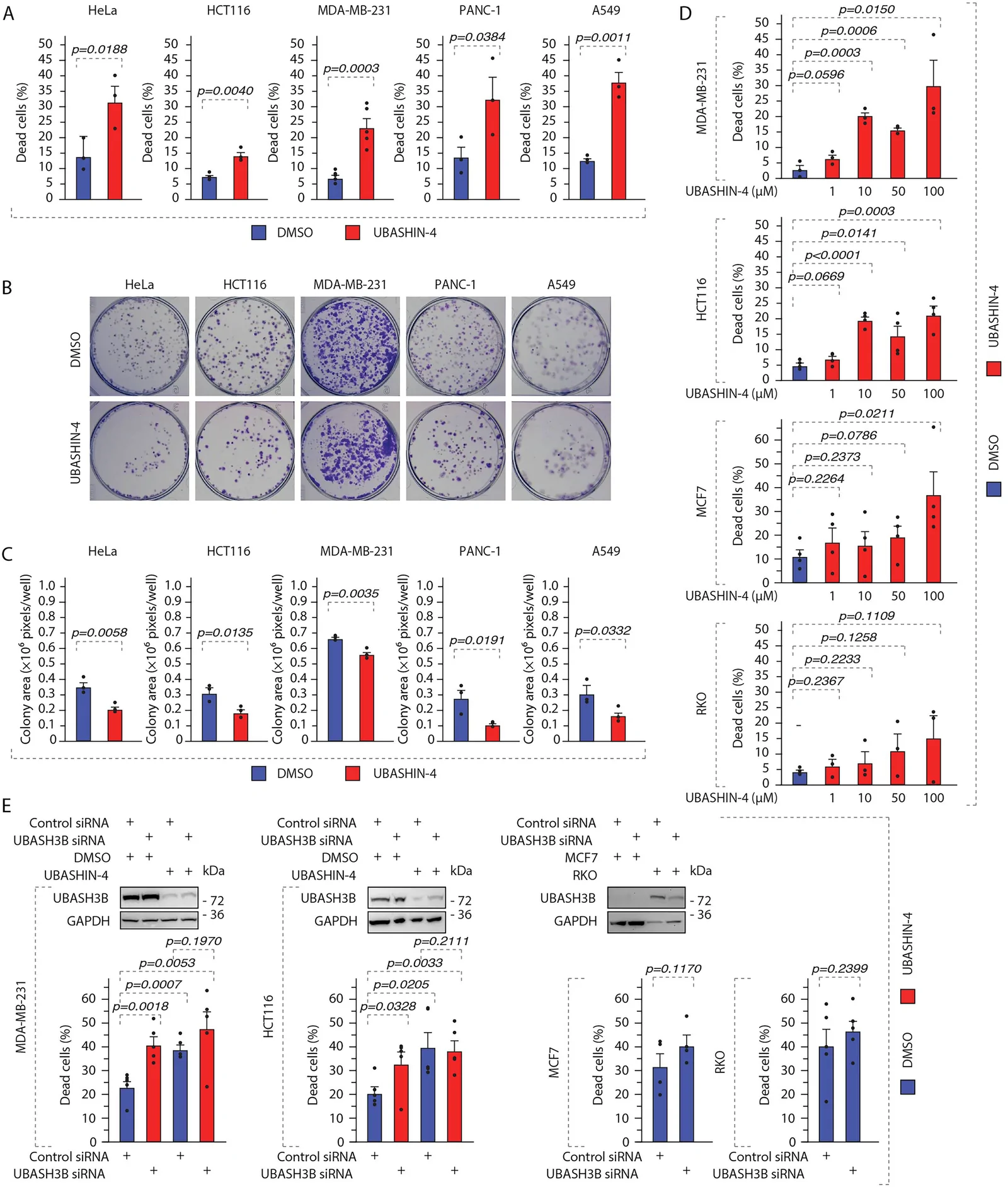
Sensitivity of cancer cells to UBASH3B inhibition. (**A**) Quantification of cell death in HeLa, HCT116, MDA-MB-231, PANC-1 and A549 cells treated with DMSO or UBASHIN-4, assessed by Trypan Blue Exclusion (TBE). Data are shown as mean ± SEM from three to five independent experiments; individual data points are shown. *P* values were calculated using one-tailed unpaired Student’s *t* tests. (**B**) Representative colony formation assay images of HeLa, HCT116, MDA-MB-231, PANC-1 and A549 cells treated with DMSO or UBASHIN-4. Colonies were stained with crystal violet. (**C**) Quantification of total colony-positive area, expressed as ×10⁶ pixels/well from the colony formation assays shown in (**B**). Data are shown as mean ± SEM from three independent experiments; individual data points are shown. *P* values were calculated using one-tailed unpaired Student’s *t* tests. (**D**) Dose-response analysis of UBASHIN-4-induced cell death in MDA-MB-231, HCT116, MCF7 and RKO cells, assessed by TBE. Data are shown as mean ± SEM from three or four independent experiments; individual data points are shown. *P* values were calculated using one-tailed unpaired Student’s *t* tests. (**E**) Comparison of UBASHIN-4 treatment and siRNA-mediated UBASH3B depletion in MDA-MB-231, HCT116, MCF7 and RKO cells. Upper panels show WB analysis of UBASH3B depletion; GAPDH was used as a loading control. Lower panels show quantification of cell death assessed by TBE under the indicated conditions. Data are shown as mean ± SEM from four or five independent experiments; individual data points are shown. *P* values were calculated using one-tailed unpaired Student’s *t* tests. Source data are available online for this figure.

**Figure EV4:**
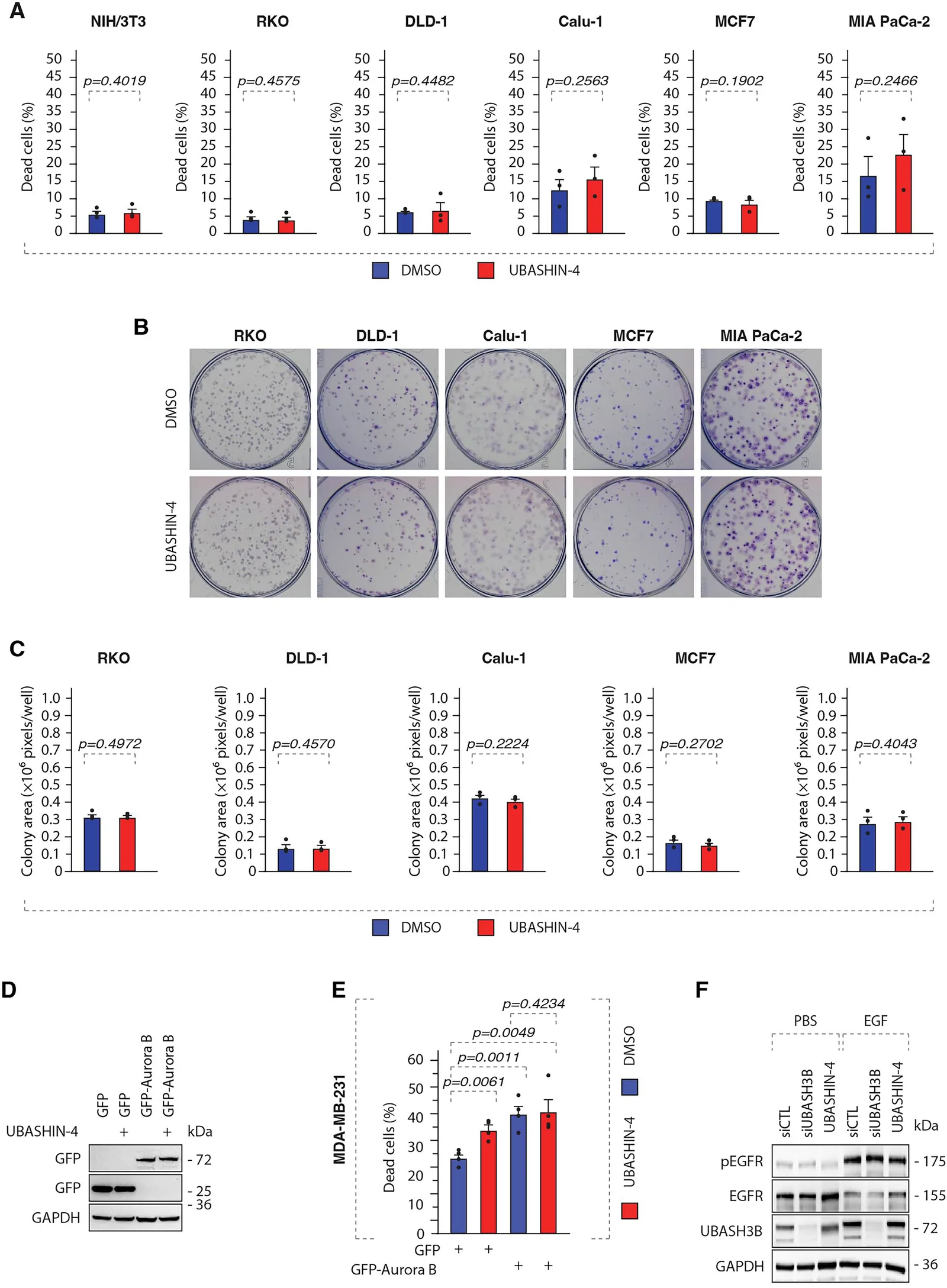
Sensitivity of cancer cells to UBASHIN is not associated with Aurora B abundance or EGFR signaling. (**A**) Quantification of cell death in NIH/3T3, RKO, DLD-1, CALU-1, MCF7 and MIA PACA-2 cells treated with DMSO or UBASHIN-4, assessed by Trypan Blue Exclusion (TBE). Data are shown as mean ± SEM from three independent experiments; individual data points are shown. *P* values were calculated using one-tailed unpaired Student’s *t* tests. (**B**) Representative colony formation assay images of RKO, DLD-1, CALU-1, MCF7 and MIA PACA-2 cells treated with DMSO or UBASHIN-4. Colonies were stained with crystal violet. (**C**) Quantification of total colony-positive area, expressed as ×10⁶ pixels/well, from the colony formation assays shown in (**B**). Data are shown as mean ± SEM from three independent experiments; individual data points are shown. *P* values were calculated using one-tailed unpaired Student’s *t* tests. (**D**) WB analysis of GFP-Aurora B overexpression in MDA-MB-231 cells treated with DMSO or UBASHIN-4. GAPDH was used as a loading control. (**E**) Quantification of cell death in MDA-MB-231 cells expressing GFP or GFP-Aurora B and treated with DMSO or UBASHIN-4, assessed by TBE. Data are shown as mean ± SEM from four independent experiments; individual data points are shown. *P* values were calculated using one-tailed unpaired Student’s *t* tests. (**F**) WB analysis of EGFR phosphorylation in MDA-MB-231 cells treated with DMSO, UBASHIN-4 or UBASH3B siRNA in the presence or absence of EGF stimulation. Cells were analyzed with antibodies against pEGFR, EGFR, UBASH3B and GAPDH.

Delays in chromosome alignment and frequent cell death upon UBASHIN treatment prompted us to evaluate its cytotoxic effects and identify the cancer cell lines most vulnerable to UBASH3B inhibition. To this end, we analyzed cancer lines derived from different tissues and displaying varying degrees of aneuploidy and CIN (Appendix Fig. S2A). Trypan Blue (TB) assays revealed that treatment with UBASHIN-4 induced significant cell death in several cell lines analyzed, including HeLa (cervical), HCT116 (colorectal), MDA-MB-231 (breast), PANC-1 (pancreatic), and A549 (lung), compared to DMSO control-treated cells (Fig. 7A). In contrast, non-responsive cell lines, including NIH/3T3 (mouse fibroblasts), RKO (colorectal), DLD-1 (colorectal), CALU-1 (lung), MCF7 (breast), and MIA PACA-2 (pancreatic), displayed minimal sensitivity to the inhibitor, with no significant increase in cell death (Fig. EV4A). Similarly, colony formation assays demonstrated reduced clonogenic growth in responsive cell lines treated with UBASHIN-4, indicating an impaired proliferative capacity (Fig. 7B,C). Because colony formation integrates both cell death and proliferative recovery after drug exposure, we cannot exclude that UBASHIN impairs both short-term viability and longer-term clonogenic growth in responsive cell lines. The non-responsive cell lines showed no significant changes in colony formation upon UBASHIN-4 treatment, indicating their resistance to UBASH3B inhibition (Fig. EV4B,C).

To delineate the concentration range over which cells respond to UBASHIN, we performed dose-response analyses in two UBASHIN-responsive cell lines, MDA-MB-231 and HCT116, and two non-responsive cell lines, MCF7 and RKO. In MDA-MB-231 and HCT116 cells, UBASHIN-4 reduced viability at concentrations as low as 10 μM, with no further increase in cell death at higher concentrations, indicating that the maximal effect was reached within the low micromolar range (Fig. 7D). In contrast, MCF7 and RKO cells remained largely unaffected up to 50 μM, confirming their resistant status (Fig. 7D). Only a small and variable decrease in viability was observed in MCF7 cells at 100 μM, possibly attributable to compound solubility limitations (Fig. 7D). Together, these results indicate that UBASHIN exerts robust cytotoxicity in a subset of cell lines within a low micromolar window, whereas other cell lines remain largely insensitive across the tested concentration range.

To directly assess whether UBASHIN cytotoxicity reflects on-target inhibition of UBASH3B, we compared pharmacological treatment with siRNA-mediated UBASH3B depletion. In UBASHIN-responsive MDA-MB-231 and HCT116 cells, UBASH3B silencing reduced viability to a similar extent as UBASHIN treatment (Fig. 7E). Moreover, combining UBASH3B knockdown with UBASHIN in these cell lines did not further increase cell death (Fig. 7E), indicating that genetic and pharmacological perturbation converge on the same mitotic pathway. In contrast, in UBASHIN-non-responsive MCF7 and RKO cells, UBASH3B knockdown did not significantly affect viability, mirroring their lack of response to UBASHIN treatment (Fig. 7E). Together, these findings support the conclusion that UBASHIN acts on-target through UBASH3B and that cellular sensitivity reflects UBASH3B engagement rather than non-specific toxicity.

### Molecular determinants of cancer cell sensitivity to UBASHIN

Having identified a subset of cancer cell lines sensitive to UBASHIN, we next sought to define the molecular characteristics associated with this sensitivity. In particular, we examined whether responsiveness correlated with UBASH3B or Aurora B abundance, SAC-associated mitotic signaling, functional responses to perturbation of the mitotic network, or pre-existing CIN.

Our results show that UBASHIN disrupts Aurora B localization (Fig. 5F,G) and reduces its localized activity on mitotic chromosomes (Fig. EV3A,B). Since elevated Aurora B expression is frequently associated with cancer aggressiveness (Vats et al, 2025), we asked whether UBASHIN remains effective in the context of Aurora B overexpression. To address this, we transiently overexpressed Aurora B in MDA-MB-231 cells and assessed cell viability by TB in the presence or absence of UBASHIN-4. Aurora B overexpression alone modestly reduced cell viability, consistent with the mitotic stress imposed by excess Aurora B activity, but did not alter the extent of cell death induced by UBASHIN-4 (Fig. EV4D,E). Together, these results indicate that UBASHIN efficacy is maintained despite increased Aurora B levels, supporting the conclusion that sensitivity is not determined by total Aurora B abundance but rather by disruption of UBASH3B-dependent Aurora B spatial regulation.

**Figure 8:**
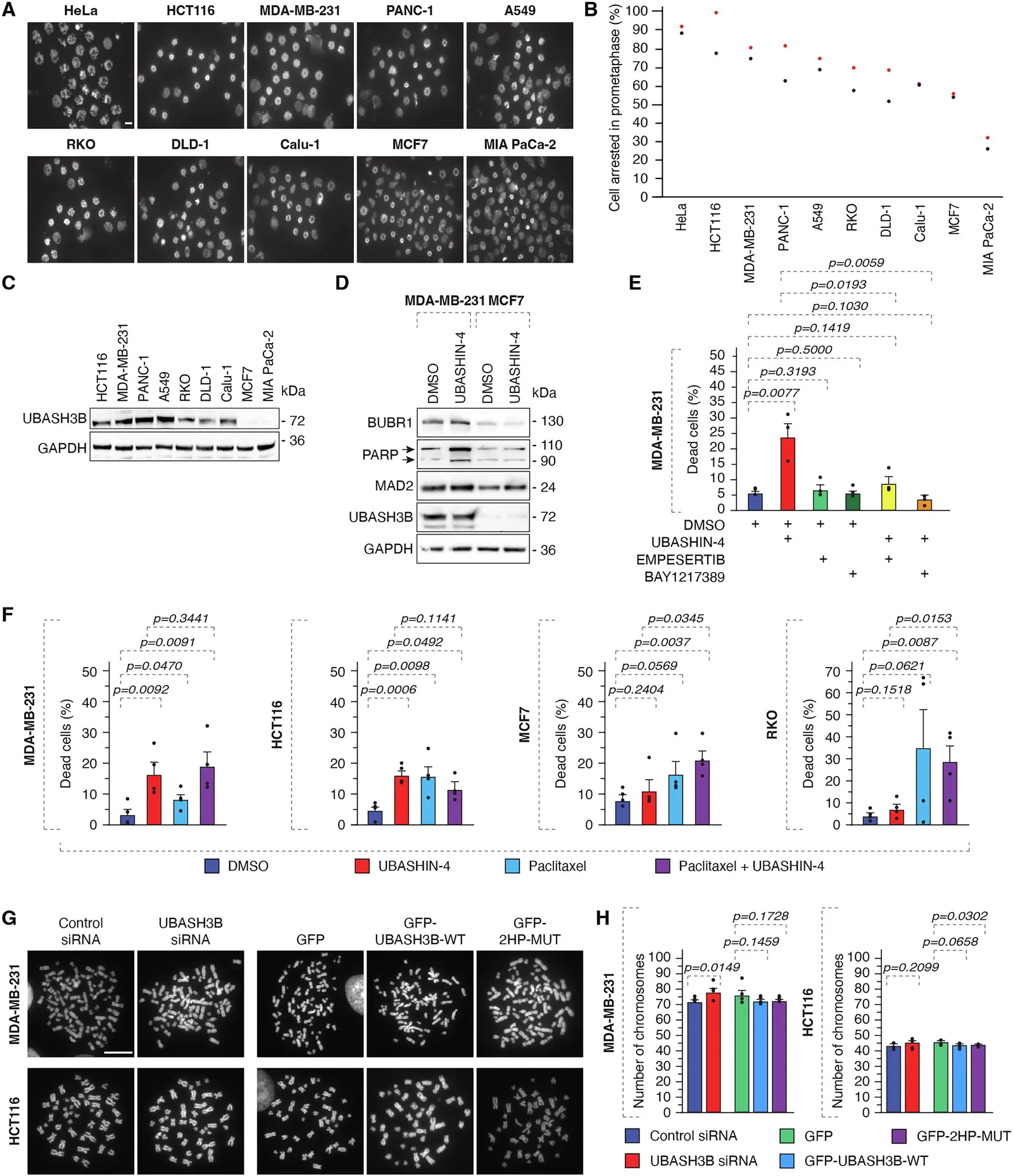
Molecular determinants of cancer cell sensitivity to UBASHIN. (**A**) Representative DAPI images of the indicated cell lines after Paclitaxel treatment to assess prometaphase accumulation. Scale bar, 15 µm. (**B**) Quantification of cells arrested in prometaphase under the conditions shown in (**A**). Individual values from two independent experiments are shown; each color represents one independent experiment. Data are presented descriptively, without error bars or inferential statistical testing. (**C**) WB analysis of UBASH3B protein levels in the indicated cell lines. GAPDH was used as a loading control. (**D**) WB analysis of BubR1, PARP1, MAD2 and UBASH3B in MDA-MB-231 and MCF7 mitotic cells treated with control siRNA or UBASH3B siRNA, in the presence or absence of UBASHIN-4. GAPDH was used as a loading control. (**E**) Quantification of cell death in MDA-MB-231 cells treated with DMSO, UBASHIN-4, MPS1 inhibitors, or the indicated combinations, assessed by TBE. Data are shown as mean ± SEM from three independent experiments; individual data points are shown. *P* values were calculated using one-tailed unpaired Student’s *t* tests. (**F**) Quantification of cell death in MDA-MB-231, HCT116, MCF7 and RKO cells treated with DMSO, UBASHIN-4, Paclitaxel, or the combination, assessed by TBE. Data are shown as mean ± SEM from four independent experiments; individual data points are shown. *P* values were calculated using one-tailed unpaired Student’s *t* tests. (**G**) Representative chromosome spreads from MDA-MB-231 and HCT116 cells after UBASH3B depletion or overexpression of GFP, GFP-UBASH3B-WT or GFP-2HP-MUT. Scale bar, 5 µm. (**H**) Quantification of chromosome number in the conditions shown in (**G**). Data are shown as mean ± SEM from four independent experiments; individual data points are shown. *P* values were calculated using one-tailed unpaired Student’s *t* tests. Source data are available online for this figure.

Our earlier findings suggested that UBASHIN affects SAC-associated mitotic signaling, as reflected by increased BubR1 kinetochore accumulation and delayed mitotic exit (Fig. 5). This prompted us to ask whether differences in mitotic arrest responses might contribute to the variable sensitivity of cancer cell lines to UBASHIN. To this end, we assessed the percentage of cells that undergo prometaphase arrest upon Paclitaxel treatment, a commonly used taxane agent known to potentiate the mitotic checkpoint through its microtubule-stabilizing activity (Yang and Horwitz, 2017). Sensitive cell lines demonstrated robust prometaphase accumulation, whereas non-responsive cell lines displayed a markedly weaker mitotic arrest response (Fig. 8A,B). Moreover, WB analysis revealed that the sensitive cell lines tended to express higher levels of UBASH3B than the non-sensitive ones (Fig. 8C), suggesting a correlation between UBASH3B abundance and the response to Paclitaxel-induced mitotic stress across the cell lines examined.

To further explore cell line-specific responses to UBASH3B inhibition, we compared expression of SAC-associated and apoptotic markers between MDA-MB-231 and MCF7 cells, two breast cancer cell lines showing differential UBASHIN sensitivity and UBASH3B expression. Notably, MDA-MB-231 cells displayed moderately higher protein levels of SAC-associated proteins, including BubR1 and MAD2, compared to MCF7 cells (Fig. 8D). UBASHIN-4 treatment did not alter the total protein levels of these markers in either cell line, suggesting that the inhibitor does not affect their expression but may instead promote their kinetochore retention in sensitive cells, consistent with enhanced mitotic checkpoint signaling (Figs. 8A,B and  5H,I). Importantly, UBASHIN-4 treatment triggered cleavage of PARP1, a marker of apoptotic cell death, in MDA-MB-231 cells, whereas MCF7 cells showed no significant increase in cleaved PARP1 levels (Fig. 8D). These results are consistent with the mitotic cell death observed in UBASHIN-treated HeLa cells (Fig. 5A,E) and support the conclusion that MDA-MB-231 cells are sensitive to UBASH3B inhibition, while MCF7 cells remain largely unaffected.

Finally, given the established role of UBASH3B in EGFR signaling in breast cancer (Lee et al, 2013), we examined whether this pathway contributes to UBASHIN-induced cell death. WB analysis demonstrated that neither UBASHIN-4 treatment nor UBASH3B knockdown affected EGFR phosphorylation in MDA-MB-231 cells upon EGF stimulation (Fig. EV4F), suggesting that UBASHIN-induced cell death is unlikely to be mediated by perturbation of EGFR signaling.

### Cancer cell sensitivity to UBASHIN is linked to SAC-associated mitotic responses rather than basal CIN

Having excluded EGFR signaling and Aurora B overexpression as major determinants of UBASHIN sensitivity, and given our earlier data pointing to altered SAC-associated mitotic signaling, we asked whether checkpoint engagement and basal CIN might help explain the differential sensitivity observed across cancer cell lines.

To further assess whether checkpoint weakening modulates UBASHIN sensitivity, we examined the efficacy of UBASHIN upon MPS1 inhibition (Bolanos-Garcia, 2025). As expected, UBASHIN-4 monotherapy induced significant cell death in MDA-MB-231 cells relative to DMSO-treated cells, as measured by TB assays (Fig. 8E). Under our short-term assay conditions (16 h), both MPS1 inhibitors tested (Empesertib and BAY1217389) did not induce significant cell death (Fig. 8E), consistent with the notion that they are more effective in synergy with taxanes under conditions of strong mitotic stress (Wengner et al, 2016; Maia et al, 2018). Interestingly, co-treatment with either MPS1 inhibitor attenuated the cytotoxic effect of UBASHIN (Fig. 8E). This result suggests that UBASHIN-induced cell death may require a checkpoint-competent mitotic state, and that MPS1 inhibition may allow cells to exit or slip through mitosis, thereby reducing the cell death signal detected in our assay.

Since MPS1 inhibition attenuated UBASHIN-induced cell death, we asked whether increasing microtubule-dependent mitotic stress via Paclitaxel (Yang and Horwitz, 2017) would enhance UBASHIN-mediated cytotoxicity. To address this, we treated UBASHIN-responsive and non-responsive cancer cell lines with UBASHIN-4, Paclitaxel, or the combination and assessed cell viability by TB assays. As expected, UBASHIN-4 alone efficiently reduced viability in MDA-MB-231 and HCT116 cells, but not in MCF7 and RKO cells (Fig. 8F). Paclitaxel alone induced cell death in UBASHIN-responsive cells and showed a modest, near-significant tendency to reduce viability in the non-responsive lines (Fig. 8F). Notably, combined treatment did not further increase cell death in responsive cell lines but markedly sensitized MCF7 and RKO cells, which became susceptible to the combination despite being resistant to UBASHIN-4 monotherapy (Fig. 8F). These data suggest that UBASH3B inhibition can cooperate with Paclitaxel-induced mitotic stress to compromise cancer cell viability in otherwise resistant cellular contexts.

To determine whether the observed effects reflected a broader impact of UBASH3B on CIN, we quantified the chromosome number after UBASH3B depletion or overexpression in two UBASHIN-responsive cell lines with markedly different karyotypic backgrounds: the highly aneuploid MDA-MB-231 cells and the nearly diploid HCT116 cells (Appendix Fig. S2A). In MDA-MB-231 cells, siRNA-mediated UBASH3B depletion led to a modest increase in chromosome number, whereas overexpression of GFP-UBASH3B-WT or GFP-2HP-MUT showed only a non-significant tendency toward reduced chromosome number (Fig. 8G,H). In HCT116 cells, UBASH3B depletion had no detectable effect, and overexpression of either construct caused only a slight, non-significant reduction in chromosome number (Fig. 8G,H).

In parallel, we analyzed TCGA datasets (Data ref: The Cancer Genome Atlas Research Network, dbGaP phs000178.v11.p8) corresponding to the cancer types represented by the cell lines tested for UBASHIN sensitivity (Appendix Fig. S2A), to assess whether UBASH3B expression correlates with aneuploidy in patient tumors. Across cancer types, UBASH3B showed only weak and inconsistent associations with aneuploidy, including a modest positive correlation in breast cancer, a modest negative correlation in colorectal and lung cancer, and no association in pancreatic cancer (Appendix Fig. S2B). Together, these data indicate that UBASH3B does not substantially alter chromosome number in the tested cell lines and is not consistently associated with aneuploidy in patient tumors, arguing against basal CIN as the primary determinant of UBASHIN sensitivity.

### UBASH3B inhibition reduces tumor growth in mouse cancer models without affecting healthy animals

Having established UBASHIN as an on-target inhibitor of UBASH3B (Fig. 7E), our data support a model in which cancer cells with dysregulated mitotic machinery depend on UBASH3B to maintain proper Aurora B spatial regulation and mitotic progression, thereby creating a mitotic vulnerability amenable to in vivo exploitation. To test this, we aimed to validate our findings in breast and colorectal cancer models, both of which show sensitivity to UBASHIN in cellular assays and represent diseases with high global mortality, together accounting for nearly 2 million deaths annually (Bray et al, 2024).

**Figure EV5:**
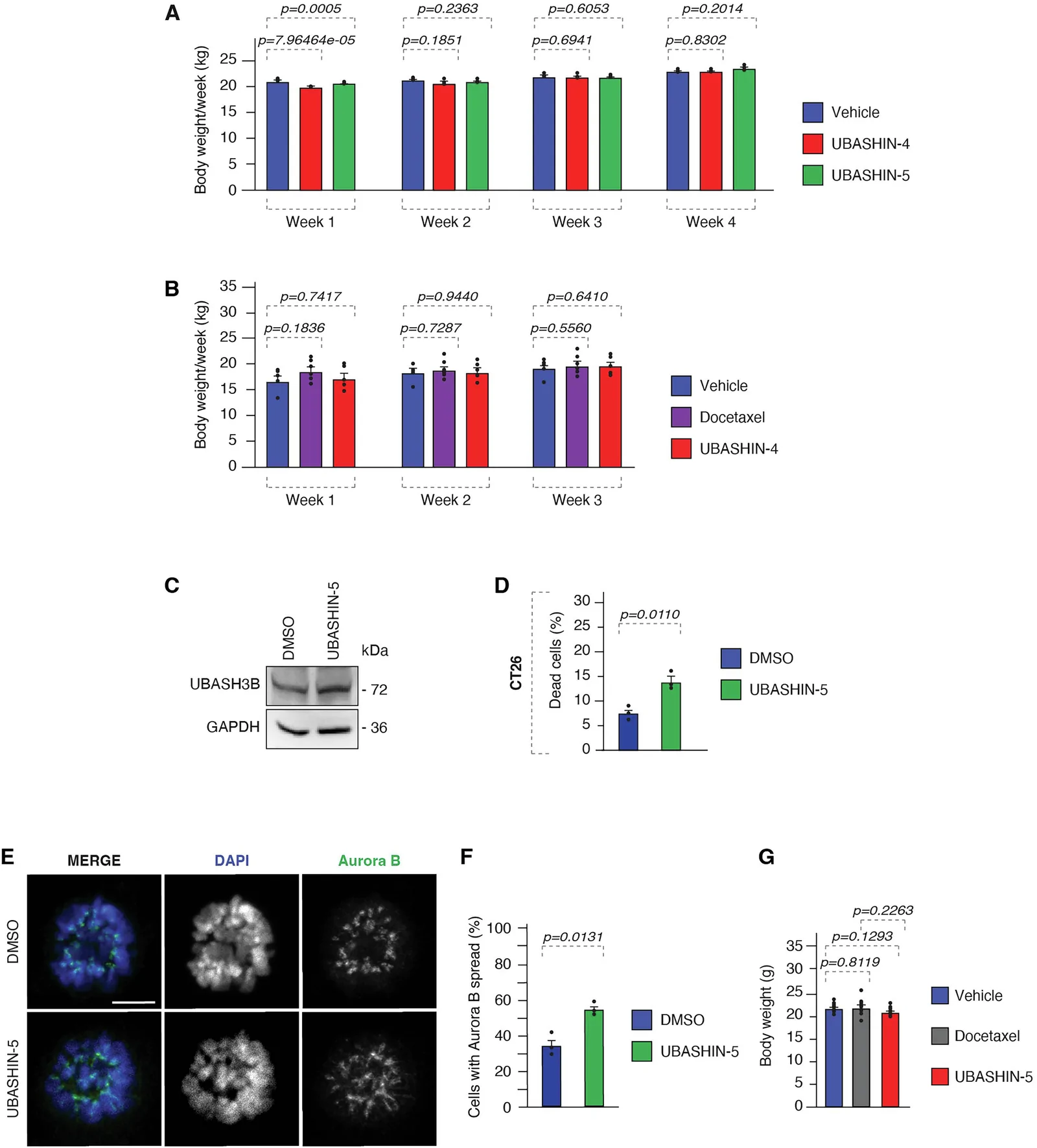
UBASHIN tolerability in vivo and CT26 cellular response to UBASH3B inhibition. (**A**) Body weight measurements of WT female Balb/c mice treated with vehicle, UBASHIN-4 or UBASHIN-5 during the pilot in vivo tolerability study. Data are shown as mean ± SEM from *n* = 3 mice per treatment group. *P* values were calculated using two-tailed Welch’s *t* tests. (**B**) Body weight measurements of mice from the breast cancer xenograft experiment treated with vehicle, docetaxel or UBASHIN-4. Data are shown as mean ± SEM from *n* = 5 vehicle-treated, *n* = 6 docetaxel-treated and *n* = 5 UBASHIN-4-treated animals included in the final analysis; *P* values were calculated using two-tailed Welch’s *t* tests. (**C**) WB analysis of UBASH3B protein levels in CT26 cells treated with DMSO or UBASHIN-5. GAPDH was used as a loading control. (**D**) Quantification of cell death in CT26 cells treated with DMSO or UBASHIN-5, assessed by TBE. Data are shown as mean ± SEM from three independent experiments; individual data points are shown. *P* values were calculated using a two-tailed Welch’s *t* test. (**E**) Representative IF images of CT26 cells treated with DMSO or UBASHIN-5 and stained for Aurora B (green) and DNA (blue, DAPI). Scale bar, 5 µm. (**F**) Quantification of cells displaying Aurora B spreading along chromosome arms under the conditions shown in (**E**). Data are shown as mean ± SEM from three independent experiments; individual data points are shown. *P* values were calculated using a two-tailed Welch’s *t* test. (**G**) Body weight measurements of mice from the syngeneic colorectal cancer model treated with vehicle, docetaxel or UBASHIN-5. Data are shown as mean ± SEM from *n* = 8 mice per group; individual data points are shown. *P* values were calculated using two-tailed Welch’s *t* tests.

To provide additional evidence for the tolerability of UBASH3B inhibitors in normal tissues (Figs. 3B and EV4A), we performed pilot in vivo experiments in WT female Balb/c (8 weeks old) mice using intraperitoneally-delivered optimized compounds UBASHIN-4 and UBASHIN-5. Throughout the experiment, no abnormal morphology or behavior was observed during daily macroscopic examinations. Additionally, no abnormal weight loss occurred in UBASHIN-treated groups, and mice continued to gain weight over the course of treatment (Fig. EV5A), in agreement with the absence of detectable phenotypes in UBASH3B KO mice (Carpino et al, 2004).

**Figure 9:**
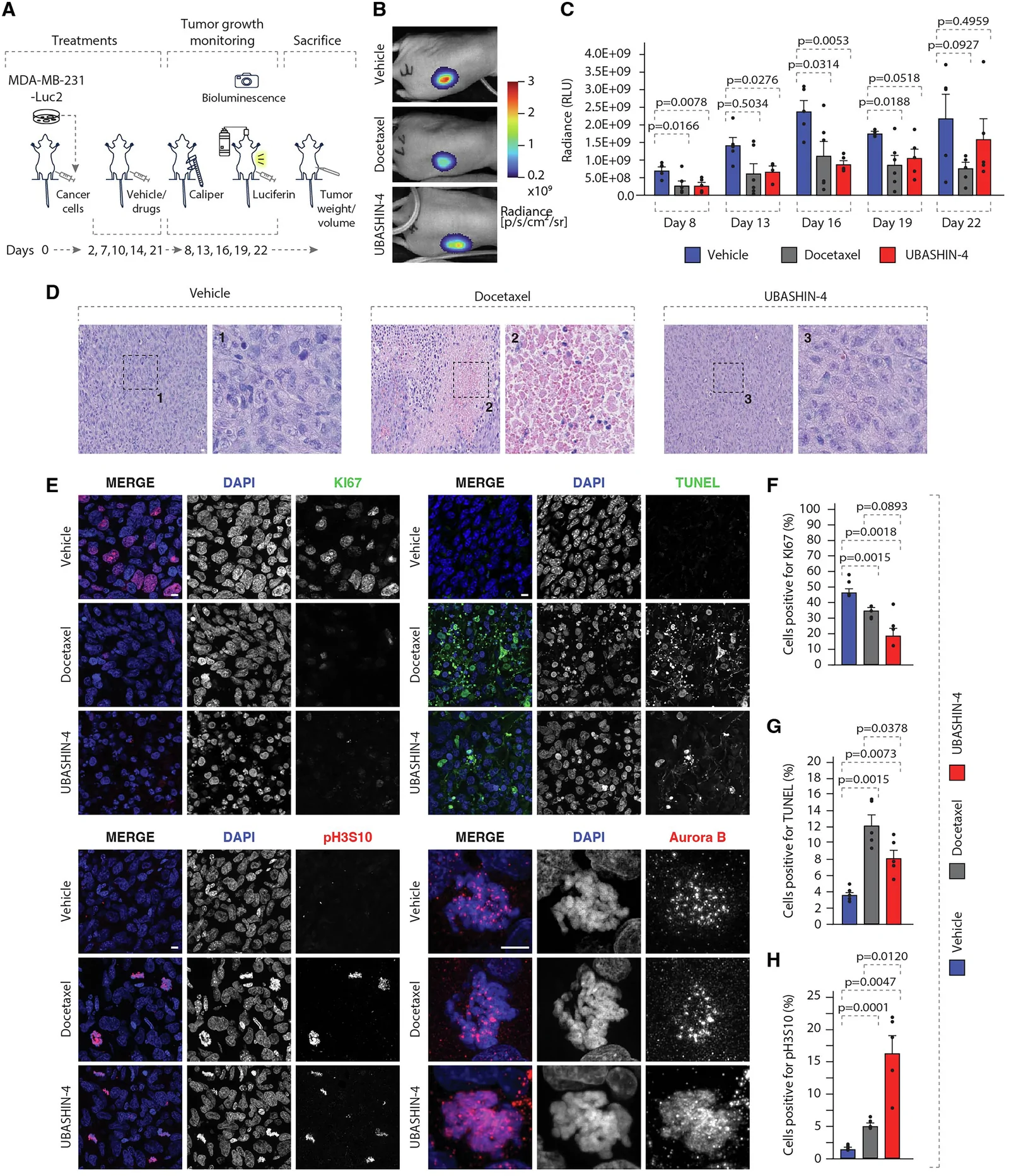
UBASH3B inhibition reduces tumor growth in a breast cancer xenograft model. (**A**) Experimental timeline for the breast cancer xenograft model. MDA-MB-231-Luc2 cells were subcutaneously injected into female Balb/c nude mice (*n* = 6 mice per group at study initiation), followed by intraperitoneal treatment with vehicle, docetaxel or UBASHIN-4, bioluminescence monitoring on days 8, 13, 16, 19 and 22, and tumor collection for histological and immunohistochemical analyses. One vehicle-treated mouse and one UBASHIN-4-treated mouse were excluded from all further analyses of this entire experiment after outlier identification using Grubbs’ test. (**B**) Representative bioluminescence images of MDA-MB-231-Luc2 xenografts from mice treated with vehicle, docetaxel or UBASHIN-4, corresponding to day 16 of the treatment period. (**C**) Quantification of tumor bioluminescence activity in relative light units (RLU) from MDA-MB-231-Luc2 xenografts in mice treated with vehicle, docetaxel or UBASHIN-4 on days 8, 13, 16, 19 and 22 of treatment. Data are shown as mean ± SEM from *n* = 5 vehicle-treated, *n* = 6 docetaxel-treated and *n* = 5 UBASHIN-4-treated animals; individual data points are shown. *P* values were calculated using two-tailed Welch’s *t* tests. Some values were excluded after outlier identification using Grubbs’ test. (**D**) Representative hematoxylin and eosin (H&E)-stained tumor sections from MDA-MB-231-Luc2 xenografts in mice treated with vehicle, docetaxel and UBASHIN-4, showing tissue morphology including necrotic regions. Scale bar, 50 µm. (**E**) Representative images of MDA-MB-231-Luc2 xenograft tumor sections stained for Ki67 (green), TUNEL (green), pH3S10 (red) or Aurora B (red). DNA was stained with DAPI. Scale bars, 20 and 5 µm. (**F**–**H**) Quantification of Ki67-positive cells (**F**), TUNEL-positive cells (**G**) and pH3S10-positive cells (**H**) from vehicle-, docetaxel- and UBASHIN-4-treated mice. Data are shown as mean ± SEM from *n* = 5 vehicle-treated, *n* = 6 docetaxel-treated and *n* = 5 UBASHIN-4-treated animals included in the final analysis; individual data points are shown. *P* values were calculated using two-tailed Welch’s *t* tests. Some values were excluded after outlier identification using Grubbs’ test. Source data are available online for this figure.

To evaluate the anticancer potential of UBASHIN in vivo, we used a breast cancer xenograft model (Fig. 9A), in which female Balb/c nude mice were subcutaneously injected with MDA-MB-231 cells. These cells express high levels of UBASH3B protein, moderately elevated levels of SAC-associated proteins (Fig. 8D), and are highly sensitive to UBASH3B inhibition in vitro (Fig. 7). This TNBC model has also demonstrated sensitivity to UBASH3B downregulation via shRNA-mediated knockdown (Lee et al, 2013). Mice were treated intraperitoneally twice per week with either UBASHIN-4, docetaxel (a taxane and standard chemotherapy agent) as a positive control, or a vehicle (solvent) as a negative control. No significant differences in body weight and general health were observed among the treatment groups throughout the experiment, confirming good tolerability of UBASHIN (Fig. EV5B).

Bioluminescence imaging revealed a significant reduction in tumor growth in UBASHIN-4- and docetaxel-treated mice relative to vehicle-treated controls over the 22-day experimental period (Fig. 9B,C). Histopathological examination of tumor sections by hematoxylin and eosin (H&E) staining showed extensive necrosis in docetaxel-treated tumors, whereas UBASHIN-4-treated tumor tissue exhibited less necrosis despite achieving a comparable tumor growth reduction (Fig. 9D). Immunohistochemical analysis revealed reduced Ki67 staining in UBASHIN-4- and docetaxel-treated tumors, indicating decreased proliferation, while TUNEL staining showed increased apoptosis relative to the vehicle group (Fig. 9E–G). Together, these results suggest that UBASHIN exerts antiproliferative effects in this TNBC model with potentially reduced cytotoxic side effects compared to docetaxel, while remaining well tolerated in healthy animals.

To link the antitumor activity of UBASHIN to its effects on mitotic progression in vivo, we analyzed tumor sections for mitotic markers by immunohistochemistry. UBASHIN-4- and docetaxel-treated tumors exhibited increased numbers of pH3S10-positive cells, a marker of mitotic entry and progression, reflecting an elevated mitotic index or delayed mitotic exit upon treatment (Fig. 9E,H). Notably, UBASHIN-treated tumors appeared to display a stronger mitotic arrest than docetaxel-treated tumors in this model.

Importantly, UBASHIN-4-treated tumors exhibited Aurora B spreading along chromosome arms, in contrast to docetaxel-treated and vehicle-control tumors, where Aurora B remained localized as puncta at the centromeres (Fig. 9E). Taken together, these results indicate that UBASHIN suppresses tumor growth in a breast cancer model by disrupting the UBASH3B-Aurora B mitotic pathway, consistent with delayed mitotic progression and increased cell death in vivo.

To assess the generalizability of these findings in an additional cancer model, we utilized a syngeneic model of colorectal cancer, in which mouse colon adenocarcinoma cells CT26 were injected subcutaneously to female WT Balb/c mice. We confirmed that CT26 cells express UBASH3B (Fig. EV5C) and are sensitive to UBASH3B inhibition in vitro. UBASHIN-5 treatment induced significant cell death in CT26 cells (Fig. EV5D) and disrupted Aurora B localization, causing its mislocalization from centromeres to chromosome arms, (Fig. EV5E,F) without substantially affecting UBASH3B protein levels (Fig. EV5C). These observations are consistent with the findings across multiple cancer cell lines of human origin (Appendix Fig. S2A).

**Figure 10:**
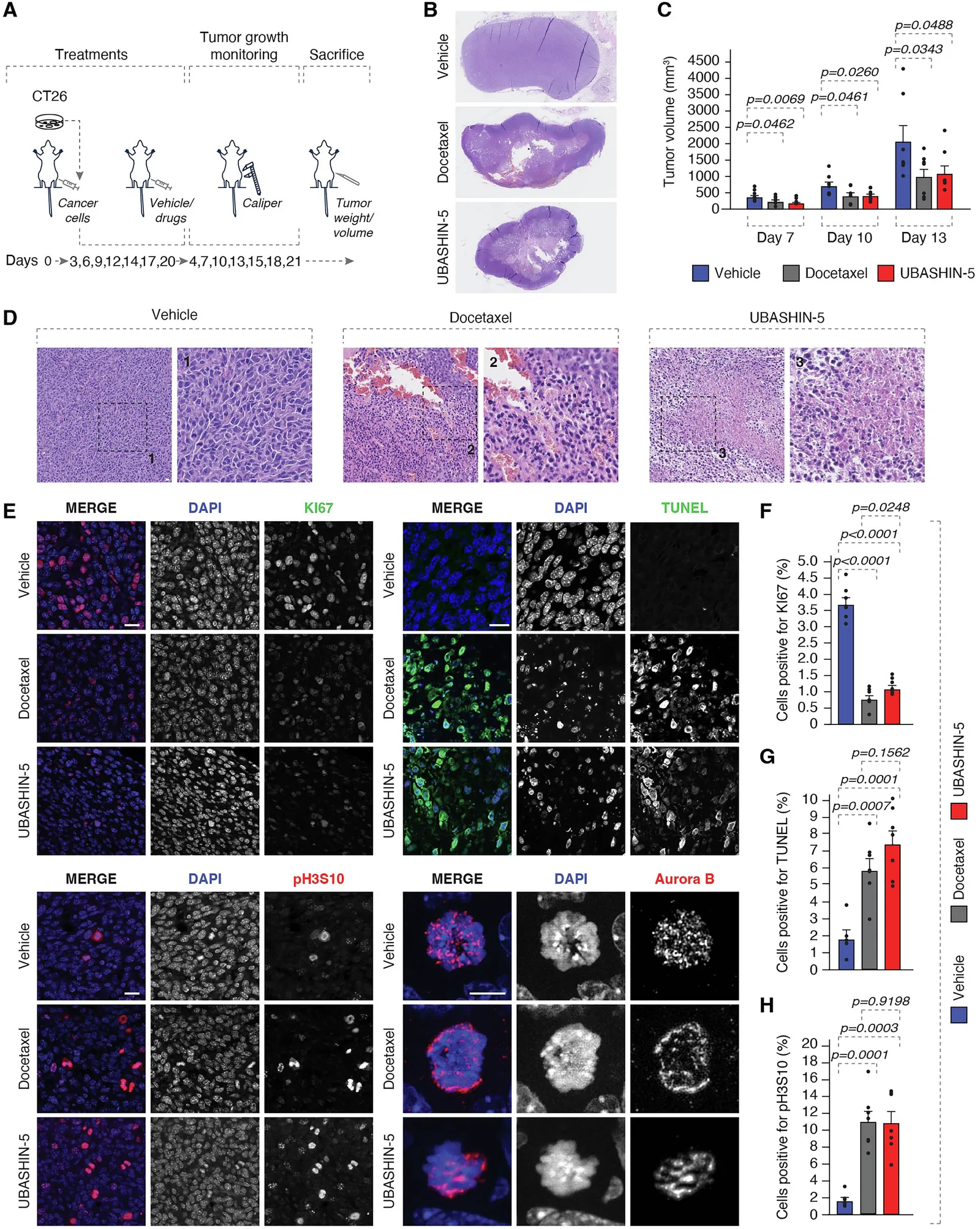
UBASH3B inhibition reduces tumor growth in a colorectal cancer mouse model. (**A**) Experimental timeline for the syngeneic colorectal cancer model. CT26 cells were subcutaneously injected into female Balb/c mice (*n* = 8 mice per group at study initiation), followed by intraperitoneal treatment with vehicle, docetaxel and UBASHIN-5. Tumor growth was monitored by caliper measurements and tumors were collected for histological and immunohistochemical analyses. (**B**) Representative images of CT26 syngeneic excised tumors from vehicle-, docetaxel- and UBASHIN-5-treated mice at the experimental endpoint, illustrating differences in tumor size and tissue morphology. Scale bar, 500 µm. (**C**) Tumor volume measurements from vehicle-, docetaxel- and UBASHIN-5-treated mice on days 7, 10 and 13. Data are shown as mean ± SEM from *n* = 7 vehicle-treated, *n* = 6 docetaxel-treated and *n* = 7 UBASHIN-5-treated animals; individual data points are shown. *P* values were calculated using one-tailed unpaired Student’s *t* tests. Some values were excluded after outlier identification using Grubbs’ test. (**D**) Representative H&E-stained of CT26 syngeneic tumor sections from vehicle-, docetaxel- and UBASHIN-5-treated mice, showing tissue morphology including necrotic regions. Scale bars, 50 µm. (**E**) Representative images of CT26 syngeneic tumor sections stained for Ki67, TUNEL, pH3S10 or Aurora B. DNA was stained with DAPI. Scale bars, 20 and 5 µm. (**F**–**H**) Quantification of Ki67-positive cells (**F**), TUNEL-positive cells (**G**) and pH3S10-positive cells (**H**) from vehicle-, docetaxel- and UBASHIN-5-treated mice. Data are shown as mean ± SEM from *n* = 7 vehicle-treated, *n* = 6 docetaxel-treated and *n* = 7 UBASHIN-5-treated animals; individual data points are shown. *P* values were calculated using two-tailed Welch’s *t* tests. Some values were excluded after outlier identification using Grubbs’ test. Source data are available online for this figure.

Mice were treated with UBASHIN-5, docetaxel, or vehicle according to the treatment schedule shown in Fig. 10A. Tumor volume was significantly reduced in both UBASHIN-5- and docetaxel-treated mice relative to the vehicle group (Fig. 10B,C), with no significant differences in body weight between experimental groups (Fig. EV5G). As in the breast cancer model, histological analysis of tumor sections revealed widespread necrosis in docetaxel-treated tumors, whereas UBASHIN-treated tumors exhibited markedly less necrosis despite comparable tumor suppression (Fig. 10D). Together, these results indicate that UBASH3B inhibition reduces tumor growth in a colorectal cancer model through a potentially less cytotoxic mechanism than docetaxel.

Finally, immunohistochemical analysis confirmed decreased Ki67 staining and increased TUNEL positivity in UBASHIN-5-treated tumors, indicating reduced proliferation and increased apoptosis (Fig. 10E–G). In agreement with the results in the TNBC model, UBASHIN-treated tumors displayed increased pH3S10 staining (Fig. 10E,H), reflecting an elevated mitotic index or delayed mitotic progression, together with Aurora B mislocalization (Fig. 10E), linking the antitumor activity of UBASHIN to disruption of the UBASH3B–Aurora B mitotic pathway.

Taken together, these findings suggest that UBASH3B inhibition represents a promising and potentially better-tolerated antimitotic strategy warranting further evaluation in breast and colorectal cancers.

## Discussion

It remains poorly understood how cancer cells tolerate high levels of mitotic errors while continuing to proliferate. We hypothesized that cancer cells may depend on specific regulators of cell division to limit additional segregation errors and survive under persistent mitotic stress. Pharmacological targeting of such factors could, in turn, represent an attractive, cancer-specific strategy to reduce cancer cell proliferation. To evaluate this hypothesis and explore the therapeutic potential of the previously characterized mitotic function of UBASH3B (Krupina et al, 2016), we first sought to identify a protein domain amenable to pharmacological targeting. Here, by combining biophysical, cellular and in vivo analyses, we provide evidence that the 2HP domain of UBASH3B constitutes a druggable vulnerability in the UBASH3B-Aurora B mitotic pathway. We further identify UBASHIN as a small-molecule inhibitor that preferentially engages the 2HP domain (Figs. 3G,H, 4A,B and EV2) and disrupts UBASH3B localization to spindle-associated structures (Fig. 6A–C), alters Aurora B localization and activity (Figs. 5F,G and EV3A,B), delays mitotic progression, and promotes mitotic cell death (Fig. 5A–E) in the context of SAC-associated mitotic signaling (Figs. 5H,I and EV3C). In vivo, UBASHIN reduces tumor growth in breast and colorectal cancer models without overt toxicity in healthy animals (Figs. 9B,C, 10B,C and  EV5A,B,G). Together, these findings support UBASH3B-dependent Aurora B regulation as a targetable mitotic vulnerability in cancer.

Mechanistically, our data identify the 2HP domain as a critical determinant of UBASH3B mitotic localization and Aurora B positioning. While our previous research highlighted the role of the UBA domain in binding monoubiquitylated Aurora B and ensuring its centromeric retention (Krupina et al, 2016), the present study shows that the 2HP domain provides an additional layer of regulation by contributing to UBASH3B localization at spindle-associated structures and Aurora B positioning at mitotic chromosomes. This distinction is important because mutation of the 2HP domain disrupts UBASH3B spindle enrichment (Fig. 2H,I), Aurora B centromeric localization (Fig. 1D,E) and mitotic progression (Fig. 2A–G), indicating that this domain is required for the mitotic function of UBASH3B. These phenotypes are consistent with the broader principle that mitotic duration is tightly constrained in mammalian cells and that even modest extensions can be biologically meaningful. Recent work has shown that mitotic delay can be sensed by threshold-dependent surveillance pathways, including p53/p21-associated responses, and that sub-threshold mitotic prolongations can be integrated across cell cycles to influence daughter-cell fate (Meitinger et al, 2024; Sparr and Meitinger, 2025; Fulcher et al, 2025). In this context, the increased chromosome segregation errors, prolonged mitotic progression and elevated mitotic cell death (Fig. 2A–G) observed upon 2HP mutation suggest that UBASH3B contributes to maintaining mitotic timing within a permissive range, and that disruption of this control can convert a normally tolerable delay into a lethal mitotic stress response.

Recent evidence further emphasizes that conserved histidine residues within the catalytic core of the 2HP domain are critical for its enzymatic function and structural integrity (Yin et al, 2020). Specifically, these residues are essential for the phosphodiesterase activity of UBASH3B, which may hydrolyze substrates such as cyclic NADP and cyclic RNA oligonucleotides. However, the precise identity and physiological relevance of these substrates in mitotic progression, spindle organization, and SAC-associated signaling remain unclear and will be the subject of future investigation. Interestingly, the catalytic 2HP mutant retains the ability to bind to the inhibitors identified in this study (Figs. 4A,B and EV2), suggesting that additional 2HP structural features contribute to inhibitor binding and may influence UBASH3B mitotic function independently of, or in addition to, its catalytic activity. This observation aligns with prior findings that the full-length UBASH3B protein displays different substrate preferences compared to its isolated 2HP domain, indicating that interdomain crosstalk may influence its conformation and specificity (Yin et al, 2020). Further studies should explore how the UBA, SH3, and 2HP domains each contribute to UBASH3B conformation, localization and mitotic function. Likewise, future efforts are needed to dissect whether UBASHIN derivatives affect the enzymatic, structural, or adaptor-like functions of the UBASH3B 2HP domain.

Our biochemical data indicate that the UBASHIN series preferentially engages the UBASH3B 2HP domain, while UBASH3A shows only weak and compound-dependent interaction, indicating partial paralog cross-reactivity (Figs. 4A,B and EV2). This observation should be interpreted in light of the distinct biology of the two TULA-family proteins. UBASH3A is predominantly expressed in T-cells and lymphoid tissues and is linked to immune signaling regulation (Wang et al, 2025; Tsygankov, 2020), whereas UBASH3B is broadly expressed and has been established as a mitotic regulator (Krupina et al, 2016, 2018). Given this functional divergence, the cellular phenotypes described here are most likely driven by UBASH3B engagement, and underscore that the UBASHIN series exploits a cancer-associated mitotic vulnerability. This is particularly relevant given the reported contrasting roles of UBASH3A and UBASH3B in cancer biology: while UBASH3B promotes tumor progression, UBASH3A has been implicated as a tumor suppressor in leukemia, inhibiting proliferation through mechanisms such as induction of HSPA1B expression (Wang et al, 2024).

The small molecule inhibitors developed in this study selectively target the mitotic function of UBASH3B and phenocopy the effects of 2HP mutation and UBASH3B downregulation on Aurora B mislocalization and prolonged mitotic arrest (Figs. 1D,E, 2A–G and 5A–G). Mechanistically, UBASHIN derivatives do not detectably interfere with the interaction of UBASH3B with Aurora B or Mklp2 (Fig. EV3D,E), nor do they affect UBASH3B protein levels (EV3C). Instead, UBASH3B inhibition disrupts its localization to the mitotic spindle (Fig. 6A–C) and leads to abnormal spindle architecture, including multipolar and monopolar spindles (Fig. 6D–I). Based on these data, we favor a model in which UBASHIN interferes with a 2HP-dependent spindle-targeting or spindle-retention function of UBASH3B, rather than acting as a general microtubule poison. This interpretation is supported by the observation that nocodazole and paclitaxel disrupt spindle organization but do not reproduce the same Aurora B chromosome-arm spreading phenotype (Fig. EV3F,G). The 2HP domain may regulate UBASH3B association with microtubules through an enzymatic or pseudoenzymatic mechanism, or by controlling a conformation required for interaction with an unidentified spindle-associated adaptor. Alternatively, UBASH3B association with spindle microtubules may be essential for maintaining proper Aurora B dynamics and, indirectly, spindle integrity, consistent with published findings that microtubules contribute to a positive feedback loop important for the centromeric recruitment and activation of Aurora B during early mitosis (Banerjee et al, 2014). Understanding how UBASH3B interacts with spindle-associated structures, and how UBASHIN disrupts this association, may facilitate the design of more selective and potent therapeutic compounds.

Our results also reveal that pharmacological targeting of UBASH3B does not affect all cancer cells equally, but rather exposes a vulnerability in selected cellular contexts (Figs. 7A–C and EV4A–C). Although UBASH3B expression tends to be higher in responsive cell lines (Fig. 8C), our data suggest that expression alone is unlikely to be sufficient as a universal predictor of sensitivity. Instead, UBASHIN responsiveness appears to reflect a broader dependence on UBASH3B-linked mitotic regulation, involving Aurora B spatial control, the ability to sustain mitotic arrest, and the cellular response to mitotic stress. In further support of this view, increasing Aurora B abundance was not sufficient to bypass UBASHIN-induced cell death (Fig. EV4D,E). This suggests that the relevant vulnerability is not total Aurora B abundance per se, but the spatial regulation of Aurora B during mitosis. In this context, UBASH3B inhibition may be particularly disruptive because it perturbs where Aurora B acts, rather than simply how much Aurora B is present. One possible consequence of altered Aurora B spatial regulation is defective correction of KT-MT attachment errors. Aurora B is a central regulator of attachment error correction (Sen et al, 2021), and its mislocalization after UBASH3B inhibition could plausibly contribute to chromosome alignment defects, segregation errors and mitotic delay. Such defects could, in principle, promote downstream consequences such as chromosome missegregation or micronuclei formation, although these specific outcomes were not directly tested here. This model is consistent with the chromosome segregation defects observed after 2HP mutation or UBASHIN treatment (Figs. 2A–F and  5A–E) and provides a possible link between UBASH3B-dependent Aurora B regulation and mitotic cell death.

Furthermore, our results suggest that the cellular response to UBASHIN depends on how mitotic stress and SAC-associated signaling are integrated. MPS1 inhibition attenuated UBASHIN-induced cell death, whereas Paclitaxel sensitized otherwise non-responsive cells to UBASHIN (Fig. 8E,F). These apparently different outcomes are not necessarily contradictory. MPS1 inhibition can weaken checkpoint signaling and promote premature mitotic exit or slippage, potentially reducing the time cells spend in the UBASHIN-induced mitotic state. In contrast, Paclitaxel imposes microtubule-dependent mitotic stress, which may expose or enhance dependence on UBASH3B-linked mitotic regulation in cells that are otherwise poorly responsive to UBASHIN alone. This interpretation is consistent with previous work showing that cell fate after antimitotic treatment is not dictated solely by the duration of mitotic arrest or by checkpoint status (Gascoigne and Taylor, 2008). Rather, competing processes that drive mitotic cell death, mitotic exit, Cyclin B1 degradation, and post-mitotic survival can collectively determine outcome. Such regulatory complexity may help explain why MPS1 inhibition and Paclitaxel differentially modify UBASHIN response, and why UBASHIN sensitivity varies across cancer cell lines.

Our data also argue against a simple model in which UBASHIN sensitivity is dictated by basal aneuploidy or pre-existing CIN. Chromosome-spread analysis showed only modest effects of UBASH3B depletion or overexpression on chromosome number, and TCGA analyses revealed weak, cancer-type-specific associations between UBASH3B expression and aneuploidy (Fig. 8G,H; Appendix Fig. S2B). These findings indicate that UBASH3B expression alone does not linearly track with CIN in primary tumors and may instead reflect context-dependent roles in constraining or accommodating CIN. Such context-specific reliance on UBASH3B may in turn sensitize cells to UBASHIN by unmasking their dependence on finely tuned mitotic regulation.

Our work also distinguishes UBASH3B’s mitotic function from its previously described role in EGFR signaling in TNBC (Lee et al, 2013). While EGFR signaling relies on the C-terminal UBASH3B phosphatase activity to dephosphorylate and inactivate the E3 ubiquitin ligase CBL (Lee et al, 2013; Kowanetz et al, 2004), we did not observe major effects on EGFR activation upon treatment with UBASHIN (Fig. EV4F). These findings suggest that the mitotic role of UBASH3B is likely mechanistically distinct from its signaling function, consistent with results showing no significant role for the PGM domain during mitosis (Fig. 1D,E). This raises the possibility that dual dependencies on UBASH3B in certain cancers may create potent therapeutic vulnerabilities, whereby combining inhibitors that target its mitotic activity with agents that modulate its phosphatase function in tyrosine kinase-driven tumors could provide a synergistic strategy and therapeutic advantage.

To effectively target the mitotic function of UBASH3B for cancer therapy, it was essential to validate our findings from cultured cancer cells in relevant animal models. Indeed, the in vivo studies provided preclinical proof-of-principle for the therapeutic potential of UBASHIN derivatives, demonstrating a significant reduction in tumor growth in breast and colorectal cancer models (Figs. 9B,C and 10B,C), diseases associated with high global mortality rates. Importantly, these inhibitors exhibit minimal toxicity in normal tissues (Fig. EV5A,B,G), which addresses a critical limitation of traditional antimitotic therapies that often result in systemic toxicity (Tischer and Gergely, 2019). While shRNA knockdown studies have previously highlighted UBASH3B’s role in metastasis (Lee et al, 2013), our pharmacological approach enables a more direct and clinically relevant means of disrupting cancer-specific mitotic functions. We demonstrate that UBASHIN not only reduces tumor growth in breast and colorectal cancer models (Figs. 9B,C and 10B,C) but also impairs Aurora B localization (Figs. 9E and 10E) and induces mitotic arrest directly in tumor tissue (Figs. 9E,H and 10E,H). Other studies on colorectal cancer identified frequent overexpression of Aurora B and have shown that targeting mitotic regulators including Aurora B, PLK1 and Mad2, can induce mitotic defects and suppress tumor growth in preclinical models (Diogo et al, 2017). Our results extend this concept by identifying UBASH3B-dependent Aurora B regulation as an additional mitotic pathway that can be pharmacologically perturbed to reduce tumor growth in vivo.

Although further studies are needed to investigate the long-term effects of UBASHIN derivatives and potential off-target interactions, our study lays the groundwork for future efforts to improve and refine their therapeutic application. In addition, validating the efficacy of UBASH3B inhibition across a broader spectrum of cancers, as well as in more physiologically relevant models such as genetically engineered or carcinogenesis-driven mouse models, will be important for defining its full clinical potential. Collectively, these findings establish UBASH3B-targeted mitotic inhibition as a promising and selective anticancer strategy warranting further clinical exploration.

## Methods

**Table:** Reagents and tools table

Reagent/resource	Reference or source	Identifier or catalog number
**Experimental models**		
HeLa Kyoto	IGBMC cell culture facility	N/A
HeLa H2B-mCherry/GFP-α-tubulin	Steigemann et al, 2009	N/A
HeLa GFP-UBASH3B-WT	This study	N/A
HeLa GFP-2HP-MUT	This study	N/A
hTERT RPE-1	ATCC	Cat# CRL-4000
GM03458 fibroblasts	IGBMC cell culture facility	N/A
F31 fibroblasts	IGBMC cell culture facility	N/A
HCT116	ATCC	Cat# CCL-247
MDA-MB2-31	ATCC	Cat# HTB-26
MDA-MB-231-Luc2	Molière et al, 2024	N/A
PANC-1	ATCC	Cat# CRL-1469
A549	ATCC	Cat# CCL-185
RKO	ATCC	Cat# CRL-2577
DLD-1	ATCC	Cat# CCL-221
Calu-1	ATCC	Cat# HTB-54
MCF7	ATCC	Cat# HTB-22
MIA PaCa-2	ATCC	Cat# CRL-1420
NIH/3T3	ATCC	Cat# CRL-1658
CT26	ATCC	Cat# CRL-2638
Female Balb/c mice, 8 weeks old (*Mus musculus*)	IGBMC/ICS animal facility	Facility approval C-67-218-37
Female Balb/c nude mice, 6 weeks old (*Mus musculus*)	IGBMC/ICS animal facility	Facility approval C-67-218-37
*Escherichia coli* BL21	NEW ENGLAND BioLabs	Cat# c2527h
**Recombinant DNA**		
pcDNA3.1-FLAG	IGBMC cloning facility	N/A
pcDNA3.1-FLAG-UBASH3B-WT	This study	N/A
pcDNA3.1-FLAG-UBASH3B-ΔPGM	This study	N/A
pcDNA3.1-FLAG-UBASH3B-PGM-MUT/H391A	This study	N/A
pcDNA3.1-FLAG-UBASH3B-2HP-MUT/H127A/T129A + H212A/T214A	This study	N/A
pEGFP-N1	Clontech	Cat# 6085-1
pEGFP -Aurora B	This study	N/A
pGEX6P1	GE Healthcare Life Sciences	Cat# 27-1542-01
pGEX6P1- UBASH3B-ΔPGM	This study	N/A
pGEX6P1- UBASH3B-UBA	This study	N/A
pGEX6P1- UBASH3B-2HP	This study	N/A
pGEX6P1- UBASH3B-SH3	This study	N/A
pGEX6P1- UBASH3B-2HP-MUT	This study	N/A
pET21a-His-BirA	Addgene	Cat# 20857
AviTag_pETM41-MBP control	This study	N/A
AviTag_pETM41-MBP-UBASH3B-2HP	This study	N/A
AviTag_pETM41-MBP-UBASH3B-2HP-MUT	This study	N/A
AviTag_pETM41-MBP-UBASH3B-FL	This study	N/A
AviTag_pETM41-MBP-UBASH3A-FL	This study	N/A
pcDNA5/FRT/TO	IGBMC cloning facility	N/A
pcDNA5/FRT/TO-GFP-UBASH3B-WT	This study	N/A
pcDNA5/FRT/TO-GFP-UBASH3B-2HP-MUT	This study	N/A
**Antibodies**		
Rabbit polyclonal anti-UBASH3B, WB 1:1000	ProteinTech	Cat# 19563-1-AP
Rabbit polyclonal anti-UBASH3B, IF 1:100	Sigma-Aldrich	Cat# HPA038605
Rat monoclonal anti-tubulin, IF 1:4000	Abcam	Cat# ab195884
Mouse polyclonal anti-Aurora B, IF 1:500	BD Biosciences	Cat# 611083
Rabbit polyclonal anti-Aurora B, WB 1:1000, IF 1:500, IHC 1:200	Abcam	Cat# ab2254
Mouse monoclonal anti-GFP, WB 1:2000	Abcam	Cat# ab3277
Human polyclonal anti-CREST, IF 1:500	Antibodies Incorporated	Cat# 15-234
Mouse monoclonal anti-BUBR1, WB 1:1000, IF 1:500	BD Biosciences	Cat# 612502
Rabbit monoclonal anti-BUBR1, IF 1:500	Invitrogen	Cat# 720297
Mouse monoclonal anti-FLAG, WB 1:1000, IF 1:1000	Sigma-Aldrich	Cat# F3165
Rabbit monoclonal anti-FLAG, WB 1:1000, IF 1:1000	Cell Signaling Technology	Cat# 14793
Mouse monoclonal anti-GST, WB 1:2000	IGBMC Monoclonal Antibody Facility	N/A
Rabbit polyclonal anti-Cyclin B1, WB 1:2000	GeneTex	Cat# GTX100911
Mouse monoclonal anti-GAPDH, WB 1:10000	GeneTex	Cat# GTX627408
Rabbit polyclonal anti-Pericentrin, IF 1:1000	Abcam	Cat# ab4448
Rabbit polyclonal anti-MAD2, WB 1:2000	Bethyl	Cat# A300-301A
Rabbit polyclonal anti-Aurora B pT232 IF 1:100	Rockland/Tebu Bio	Cat# 600-401-677
Rabbit polyclonal anti-PARP, WB 1:1000	Cell Signaling Technology	Cat# 9542
Rabbit polyclonal anti-EGFR, WB 1:1000	Proteintech	Cat# 18986-1-AP
Rabbit polyclonal anti-phospho-EGFR Tyr1068, WB 1:1000	Cell Signaling Technology	Cat# 234S
Rabbit monoclonal anti-Ki-67, IHC 1:200	Thermo Fisher Scientific	Cat# MA5-14520
Rabbit polyclonal anti-phospho-Histone H3 Ser10 / pH3S10, WB 1:1000, IHC 1:200	Millipore	Cat# 06-570
Rabbit polyclonal anti-Kif20A / Mklp2, WB 1:1000	Proteintech	Cat# 15911-1-AP
Mouse polyclonal anti-MBP, WB 1:2000	New England Biolabs	Cat# E8032S
Rabbit polyclonal anti-Bub1, WB 1:1000	Genetex	Cat# GTX107497
Goat anti-mouse Alexa Fluor 488, IF:500	Life Technologies	Cat# W3007B
Goat anti-mouse Alexa Fluor 555, IF:500	Life Technologies	Cat# A-21424
Goat anti-mouse Alexa Fluor 647, IF:500	Life Technologies	Cat# A-21236
Goat anti-rabbit Alexa Fluor 488, IF:500	Life Technologies	Cat# A-11034
Goat anti-rabbit Alexa Fluor 555, IF:500	Life Technologies	Cat# A-21429
Goat anti-rabbit Alexa Fluor 647, IF:500	Life Technologies	Cat# A-21245
Goat anti-human Alexa Fluor 488, IF:500	Life Technologies	Cat# A-11013
Goat anti-human Alexa Fluor 555, IF:500	Life Technologies	Cat# A-21433
Goat anti-rat Alexa Fluor 633, IF:500	Life Technologies	Cat# A-21094
Goat anti-rabbit IgG-HRP conjugate, WB 1:5000	Biorad	Cat# 1706515
Goat anti-mouse IgG-HRP conjugate, WB 1:5000	Biorad	Cat# 1706516
**Oligonucleotides and other sequence-based reagents**		
Control siRNA/non-targeting siRNA	Dharmacon	Cat# D-001210-02-05
UBASH3B siRNA, Hs_STS-1_5 FlexiTube siRNA	Qiagen	Cat# SI02647022
UBASH3B siRNA, Hs_STS-1_7 FlexiTube siRNA	Qiagen	Cat# SI02647036
GST-UBASH3B-ΔPGM forward primer, EcoRI	This study	5′-AATAATGAATTCATGGCTCAGTACGGCCACCCCAGTCCGCTCGGCAG-3′
GST-UBASH3B-ΔPGM reverse primer, SalI	This study	5′-ATAATGTCGACTCATTCATCAGCCTTAATGTAATTCTCAGC-3′
GST-UBASH3B-SH3 forward primer, EcoRI	This study	5′-AATAATGAATTCATGGCTAACCATGAGACATTACAGGTCATCTACC-3′
GST-UBASH3B-SH3 reverse primer, SalI	This study	5′-ATAATGTCGACTCATTCATCAGCCTTGGTAATGTAATTCTCAGGC-3′
GST-UBASH3B-2HP forward primer, EcoRI	This study	5′-AATAATGAATTCATGTCCCATGTCGGTGACCCCTTC-3′
GST-UBASH3B-2HP reverse primer, SalI	This study	5′-AATAATGTCGACTCAAAATCGGATATCCCGAGAAAATATG-3′
GFP-UBASH3B forward primer, XhoI	This study	5′-AATAATCTCGAGATGGCTCAGTACGGCCACCCC-3′
GFP-UBASH3B reverse primer, BamHI	This study	5′-AATAATGGATCCTTATTCTTGAAGCAAGGTCTC-3′
GFP-UBASH3B-WT/ GFP-2HP-MUT stable-cell-line forward primer, KpnI	This study	5′-AATAATGGTACCATGGTGAGCAAGGGCGAGGAG-3′
GFP-UBASH3B-WT/ GFP-2HP-MUT stable-cell-line reverse primer, NotI	This study	5′-AATAATGCGGCCGCTTATTCTTGAAGCAAGGTCTC-3′
FLAG-UBASH3B-ΔPGM forward primer, BamHI	This study	5′-AATAATGGATCCATGGATTACAAGGATGACGACGATAAGGCTCAGTACGGCCACCCCAGTCCG-3′
FLAG-UBASH3B-ΔPGM reverse primer, XhoI	This study	5′-ATAATCTCGAGTTATTCATCAGCCTTGGTAATGTAATTCTCAGGC-3′
FLAG-UBASH3B-WT/ FLAG-UBASH3B-2HP-MUT forward primer, BamHI	This study	5′-AATAATGGATCCATGGATTACAAGGATGACGACGATAAGGCTCAGTACGGCCACCCCAGTCCG-3′
FLAG-UBASH3B-WT/ FLAG-UBASH3B-2HP-MUT reverse primer, XhoI	This study	5′-AATAATCTCGAGTTATTCTTGAAGCAAGGTCTCTC-3′
UBASH3B H127A/T129A mutagenesis forward primer	This study	5′-CACAACATCTTCCCCGCCATCGCACTCTGCCAGTTCTTTATGTGCG-3′
UBASH3B H127A/T129A mutagenesis reverse primer	This study	5′-CATAAAGAACTGGCAGAGTGCGATGGCGGGGAAGATGTTGTGTGCC-3′
UBASH3B H212A/T214A mutagenesis forward primer	This study	5′-CATAAGAAGCAGCTAGCTGTGGCCCTGGCTTACCACTTCCAAGC-3′
UBASH3B H212A/T214A mutagenesis reverse primer	This study	5′-GAAGTGGTAAGCCAGGGCCACAGCTAGCTGCTTCTTATGAGG-3′
**Chemicals, enzymes and other reagents**		
jetPEI	Polyplus transfection	Cat# 101-01 N
Lipofectamine RNAiMAX	Thermo Fisher Scientific/Invitrogen	Cat# 13778150
Thymidine	Sigma-Aldrich	Cat# T1895-5G
Monastrol	Sigma-Aldrich	Cat# M8515
STLC / S-trityl-L-cysteine	Enzo Life Sciences	Cat# ALX-105-011-M500
Paclitaxel	Sigma-Aldrich	Cat# T-7191
Nocodazole	Sigma-Aldrich	Cat# M-1404
Empesertib	Selleck	Cat# S8214
BAY1217389	Selleck	Cat# S8215
EGF	Sigma-Aldrich	Cat# SRP3027
LIVE/DEAD Fixable Dead Cell Stain kit	Merck-Millipore	Cat# 04511-1KT-F
4′,6-Diamidino-2-phenylindole dihydrochloride (DAPI)	Sigma-Aldrich	Cat# D8417
MOWIOL 4-88 Reagent	Millipore	Cat# 475904-M
SiR-DNA	Sprirochrom	Cat# SC007
In Situ Cell Death Detection Kit	Roche	Cat# 11684795910
D-luciferin	MedChemExpress	Cat# HY-12591A
UBASHIN-1/FD-E09	This study	N/A
UBASHIN-2	This study	N/A
UBASHIN-3	This study	N/A
UBASHIN-4 /LIT-13-108	This study	N/A
UBASHIN-5/LIT-14-132	This study	N/A
Negative control compound SR-12-40	This study	N/A
DMEM high glucose, 4.5 g/L glucose	IGBMC cell culture facility	N/A
DMEM low glucose, 1 g/L glucose	IGBMC cell culture facility	N/A
DMEM/Ham’s F12	IGBMC cell culture facility	N/A
Minimum Essential Medium/MEM	IGBMC cell culture facility	N/A
MEM alpha/α-MEM	IGBMC cell culture facility	N/A
RPMI medium without HEPES	IGBMC cell culture facility	N/A
Fetal calf serum/FCS	IGBMC cell culture facility	N/A
Penicillin-streptomycin	IGBMC cell culture facility	N/A
Gentamycin	IGBMC cell culture facility	N/A
Non-essential amino acids/NEAA	IGBMC cell culture facility	N/A
Sodium pyruvate	IGBMC cell culture facility	N/A
Deoxyribonucleosides	IGBMC cell culture facility	N/A
Blasticidin	IGBMC cell culture facility	N/A
Puromycin	IGBMC cell culture facility	N/A
**Software**		
Fiji Image Analysis	https://imagej.net/Fiji	N/A
QuPath	https://qupath.github.io/	N/A
NDP.view2	Hamamatsu	Cat# U12388-01
Ilastik	https://www.ilastik.org/	N/A
TCGA tumor datasets	dbGaP phs000178.v11.p8	TCGA-BRCA, TCGA-COAD/READ, TCGA-LUAD/LUSC, TCGA-PAAD cohorts

### Cell lines and culture conditions

Cell lines were obtained from the sources indicated in the Reagents and Tools Table, including ATCC where applicable, and maintained for limited passages. Cell cultures were regularly tested for mycoplasma contamination by the IGBMC cell culture facility and were negative. Cells were maintained at 37 °C in a humidified incubator containing 5% CO₂. Unless otherwise stated, penicillin-streptomycin (P/S) was used at 1% and gentamicin at 50 µg/mL. HeLa Kyoto cells were cultured in high-glucose DMEM containing 4.5 g/L glucose, supplemented with 10% fetal calf serum (FCS) and P/S. HeLa cells stably expressing H2B-mCherry and EGFP-α-tubulin were cultured in high-glucose DMEM containing 4.5 g/L glucose, supplemented with 5% FCS and P/S, and maintained with blasticidin (0.5 µg/mL) and puromycin (0.5 µg/mL). HeLa GFP-UBASH3B-WT and HeLa GFP-2HP-MUT inducible cell lines were cultured in high-glucose DMEM supplemented with 10% FCS and P/S, and maintained with hygromycin B (200 µg/mL). GM03458 and F31 fibroblasts were cultured in high-glucose DMEM-GlutaMAX supplemented with 10% FCS, 1 mM sodium pyruvate and gentamicin. RKO cells were cultured in Minimum Essential Medium supplemented with 10% FCS, non-essential amino acids, 1 mM sodium pyruvate and gentamicin. HCT116 cells were cultured in low-glucose DMEM containing 1 g/L glucose, supplemented with 7.5% FCS and gentamicin. A549 cells were cultured in DMEM/Ham’s F12 supplemented with 10% FCS and gentamicin. PANC-1 cells were cultured in high-glucose DMEM containing 4.5 g/L glucose, supplemented with 10% FCS, 1 mM sodium pyruvate and gentamicin. DLD-1, MDA-MB-231 and CT26 cells were cultured in RPMI medium without HEPES, supplemented with 10% FCS and gentamicin. MCF7 and MIA PACA-2 cells were cultured in low-glucose DMEM containing 1 g/L glucose, supplemented with 10% FCS, 2.5% FCS and gentamicin. CALU-1 cells were cultured in MEMα supplemented with deoxyribonucleosides, 10% FCS and gentamicin. NIH/3T3 cells were cultured in DMEM supplemented with 10% FCS and gentamicin. hTERT HTERT RPE-1 cells were cultured in DMEM/F12 supplemented with 10% FCS, P/S and hygromycin B.

### Plasmids, siRNAs and transfections

Plasmid and siRNA transfections were performed using the reagents listed in the Reagents and Tools Table, according to the manufacturers’ instructions. For siRNA-mediated silencing, cells were transfected with control siRNA or a mix of two UBASH3B-targeting siRNAs using Lipofectamine RNAiMAX. Unless otherwise stated, cells were seeded in six-well plates 24 h before transfection and transfected with 40 pmol total siRNA per well. For UBASH3B depletion, 20 pmol of each UBASH3B-targeting siRNA was combined. Cells were collected or processed for downstream experiments 48 h after transfection. For plasmid expression experiments, cells were transfected using jetPEI. Unless otherwise stated, cells were seeded in 6-well plates 24 h before transfection and transfected with 2 µg plasmid DNA per well. Cells were collected or processed for downstream experiments 48 h after transfection.

### Molecular cloning

UBASH3B constructs used for cell-based experiments were generated by PCR amplification with a high-fidelity DNA polymerase, restriction enzyme cloning and site-directed mutagenesis, using the plasmid backbones and primers listed in the Reagents and Tools Table. FLAG-tagged UBASH3B constructs used for rescue experiments were cloned into pcDNA3.1-FLAG. GFP-tagged UBASH3B constructs used for generation of inducible stable cell lines were cloned into pcDNA5/FRT/TO. GST-tagged UBASH3B fragments used for recombinant protein production and fluorescence polarization assays were cloned into pGEX6P1. Site-directed mutagenesis was used to generate the 2HP-MUT and PGM-MUT/H391A constructs. The 2HP-MUT construct carries alanine substitutions in the conserved catalytic motifs of the 2HP domain, H127A/T129A and H212A/T214A. For SPR experiments, MBP-fusion constructs encoding MBP alone, MBP-UBASH3B-2HP, MBP-UBASH3B-2HP-MUT, MBP-UBASH3B-FL and MBP-UBASH3A-FL were cloned into AviTag_pETM41. AviTag_pETM41 constructs were co-expressed with pET21a-His-BirA to allow BirA-mediated biotinylation during bacterial expression. All constructs were verified by sequencing or by the relevant internal validation before use.

### Generation of stable GFP-UBASH3B cell lines

Stable inducible HeLa cell lines expressing GFP-UBASH3B-WT or GFP-UBASH3B-2HP-MUT were generated using the Flp-In T-REx system. GFP-tagged UBASH3B constructs cloned into pcDNA5/FRT/TO were co-transfected with pOG44 into Flp-In T-REx HeLa cells. Stable integrants were selected with hygromycin B (200 µg/mL). Single-cell clones were isolated by fluorescence-activated cell sorting after doxycycline induction, and clones expressing GFP-tagged UBASH3B at levels close to endogenous UBASH3B were selected for further experiments. For experiments using these stable lines, GFP-UBASH3B expression was induced with doxycycline (1 µg/mL) for 24 h before cell collection or downstream analysis.

### Cell synchronization

For double thymidine block and release (DTBR), cells were treated with thymidine (2 mM) for 16 h, released into fresh medium for 8 h, and treated again with thymidine (2 mM) for 16 h; the mitotic peak occurred approximately 9 h after the second release. Prometaphase enrichment was performed using monastrol (100 µM, 16 h), STLC (5 µM, 16 h), paclitaxel (1 µM, 16 h) or nocodazole (100 ng/mL, 16 h), depending on the experiment. For monastrol-release experiments, cells were washed three times with pre-warmed medium and released into fresh pre-warmed medium for the indicated time-points before analysis.

### Drug treatments

Unless otherwise stated, UBASHIN compounds were used at 10 µM for 16 h before collection or further analysis, and DMSO was used as vehicle control. For UBASHIN time-course and dose-response experiments, treatment times and concentrations are indicated in the corresponding figures. For MPS1 inhibition experiments, cells were treated for 16 h with UBASHIN-4 (10 µM) Empesertib (100 nM), BAY1217389 (100 nM), or the indicated combinations before Trypan Blue exclusion analysis. For paclitaxel co-treatment experiments, cells were treated for 16 h with UBASHIN-4 (10 µM), paclitaxel (1 µM), or the combination before Trypan Blue exclusion analysis. For EGFR pathway analysis, cells were treated with DMSO, UBASHIN-4 or UBASH3B siRNA as indicated, and stimulated with EGF (100 ng/mL) for 1 h before collection for western blot analysis of EGFR phosphorylation.

### Immunofluorescence microscopy and image analysis

For cytospin-based immunofluorescence, mitotic cells were collected by scraping and centrifuged onto glass slides using a cytocentrifuge for 5 min at 1000 rpm. Cells were fixed with 4% paraformaldehyde for 10 min at room temperature, washed with PBS, permeabilized with 0.5% NP-40 for 5 min and blocked with 3% BSA in PBS containing 0.01% Triton X-100. Cells were incubated with primary antibodies for 1 h at room temperature, washed, and incubated with fluorescent secondary antibodies for 45 min at room temperature in the dark. DNA was stained with DAPI and samples were mounted in Mowiol.

For adherent-cell immunofluorescence, cells were grown directly on coverslips and processed using the same fixation, permeabilization, blocking and antibody-incubation conditions. For endogenous UBASH3B localization analyses, cells were processed using a PHEM-based extraction/fixation protocol to preserve the spindle-associated UBASH3B pool. Because this extraction condition can affect spindle organization, all compared samples were processed in parallel using the same protocol, and spindle-organization analyses were performed only between matched conditions.

Images were acquired using epifluorescence or spinning-disk confocal microscopy with a ×63 objective. Z-stacks were acquired over a 6 µm range with 0.5 µm steps and processed as maximum-intensity projections. Image analysis was performed using Fiji/ImageJ. For Aurora B localization analyses, mitotic cells were classified according to whether Aurora B was concentrated at centromeres or spread along chromosome arms. For pAurora B T232 analyses, cells were scored for phospho-Aurora B signal spreading along chromosome arms. For BubR1 analyses, kinetochore BubR1 signal intensity was quantified and normalized to CREST. For UBASH3B localization analyses, cells were scored according to enrichment of endogenous or GFP-tagged UBASH3B at spindle-associated structures. Unless otherwise stated, at least 100 cells were analyzed per condition and per experiment. All antibodies and fluorescent secondary antibodies are listed in the Reagents and Tools Table.

### Live-cell microscopy and mitotic phenotype analysis

Live-cell microscopy was performed as previously described, with minor modifications (Pangou et al, 2021; Guerber et al, 2023). Cells were seeded in 35-mm glass-bottom dishes with four compartments and maintained at 37 °C and 5% CO₂ during imaging. Where indicated, SiR-DNA was added 1 h before imaging to visualize chromosomes. DTBR-synchronized cells were imaged 8 h after release using a Yokogawa W1 spinning-disk system equipped with a Leica 63×/1.0 water-immersion objective. Images were acquired for 8 h every 5–10 min as Z-stacks spanning 25 µm with 2 µm steps. Image analysis was performed using Fiji/ImageJ. Mitotic timing was measured from prophase onset or nuclear envelope breakdown to anaphase onset, mitotic exit, mitotic cell death or the end of the movie, depending on the experiment. Cells were further classified for chromosome segregation errors, prolonged mitotic arrest, cell death during mitosis or cell death after mitotic exit, as indicated in the corresponding analyses.

### Western blotting and GFP-UBASH3B immunoprecipitation

Western blotting was performed as previously described (Guerber et al, 2023), with minor modifications. Cells were collected by centrifugation, washed with cold PBS and lysed in RIPA buffer supplemented with protease and phosphatase inhibitors. Lysates were incubated on ice for 30 min and clarified by centrifugation at 14,000 rpm for 30 min at 4 °C. Protein concentration was measured using Bradford assay. Samples were boiled in Laemmli buffer containing β-mercaptoethanol, resolved on 4–12% Bis-Tris gradient gels and transferred to PVDF membranes. Membranes were blocked in milk- and/or BSA-containing buffer, incubated with primary antibodies overnight at 4 °C, washed in TBS-T, incubated with HRP-conjugated secondary antibodies and developed using chemiluminescent HRP substrate. For GFP-UBASH3B immunoprecipitation, mitotically enriched HeLa cells expressing GFP-UBASH3B were lysed in Triton X-100-based IP buffer supplemented with protease inhibitors. Cleared lysates were incubated with GFP-Trap agarose beads, and bound proteins were washed and eluted in Laemmli buffer containing β-mercaptoethanol before western blot analysis. All antibodies are listed in the Reagents and Tools Table.

### Trypan Blue exclusion assay

Cell death was assessed by Trypan Blue exclusion. Unless otherwise stated, 75,000 cells/well were seeded in 12-well plates 24 h before treatment. After treatment, cells were collected by trypsinization; for paclitaxel-containing conditions, cells were collected by scraping to retain rounded mitotic cells. Cell suspensions were mixed 1:1 with 0.4% Trypan Blue and viable and dead cells were counted using a hemocytometer or automated counter. The percentage of dead cells was calculated as the number of Trypan Blue-positive cells divided by the total number of cells counted.

### Flow-cytometry-based cell death analysis

Cell death was analyzed by flow cytometry using the LIVE/DEAD Fixable Dead Cell Stain kit according to the manufacturer’s instructions. Cells were seeded in 6-well plates and treated the following day with DMSO vehicle or UBASHIN-1 (1 µM) for 24 h. Cells were then stained with the fluorescent viability dye and analyzed by flow cytometry. Debris was excluded based on forward- and side-scatter profiles, and the percentage of dead cells was calculated as the fraction of LIVE/DEAD-positive cells among total gated cells.

### Colony formation assay

Colony formation assays were performed as previously described, with minor modifications (Pangou et al, 2021). 500 cells were seeded in six-well plates in technical triplicates and treated with DMSO or UBASHIN-4 (10 µM). Treatment was renewed every 48 h, and colonies were allowed to form for 14 days. Cells were then washed with PBS, fixed with 4% paraformaldehyde and stained with 0.1% crystal violet for 30 min. Plates were imaged macroscopically and total colony-positive area per well was quantified using an automated Fiji/ImageJ pipeline.

### Chromosome spread analysis

Chromosome spread analysis was performed to assess chromosome number after UBASH3B depletion or overexpression. Cells were seeded in six-well plates, transfected with siRNAs or plasmid DNA as described above and were processed 48 h after transfection. To enrich metaphase cells, colcemid was added at 1 µg/mL for 3 h before collection. Cells were then trypsinized, collected by centrifugation and incubated in ice-cold 0.56% KCl for 6 min at room temperature. Cells were fixed by dropwise addition of ice-cold methanol/acetic acid (3:1), followed by one to two additional fixation washes. Fixed cells were resuspended in methanol/acetic acid and dropped onto alcohol-cleaned glass slides. Slides were air-dried for at least 1 h at room temperature and mounted with DAPI-containing mounting medium. Chromosomes were imaged using fluorescence microscopy, and chromosome numbers were counted from complete, single-cell metaphase spreads.

### Recombinant protein expression and purification

GST-tagged UBASH3B fragments were expressed in *E. coli* BL21. Protein expression was induced with IPTG (0.5 mM) overnight at 20 °C when bacterial cultures reached an OD₆₀₀ of 0.6–0.8. Bacterial pellets were resuspended in GST lysis buffer (50 mM Tris-HCl pH 7.5, 150 mM NaCl, 10% glycerol, 3 mM MgCl₂, 1 mM DTT and 1% Triton X-100), lysed by sonication and clarified by centrifugation. GST-tagged proteins were purified using glutathione sepharose beads, washed in lysis buffer and eluted with reduced glutathione. Where indicated, GST was cleaved by overnight incubation with PreScission protease at 4 °C.

Biotinylated MBP-fusion proteins used for SPR experiments were expressed in *E. coli* BL21(DE3) with His-BirA for BirA-mediated biotinylation during bacterial expression. Protein expression was induced with IPTG (1 mM) overnight at 18 °C in LB medium supplemented with biotin (50 µM). Bacterial pellets from 3 L cultures were resuspended in MBP lysis buffer (50 mM Tris-HCl pH 7.0, 400 mM NaCl, 5 mM DTT and 50 µM biotin), lysed by sonication and clarified by ultracentrifugation. MBP-fusion proteins were purified by amylose affinity chromatography and eluted with maltose, followed by desalting, ion-exchange chromatography and size-exclusion chromatography. Purified biotinylated proteins were concentrated, snap-frozen and stored at −80 °C.

### GST pull-down assay

GST pull-down assays were performed as previously described (Krupina et al, 2016), with minor modifications. Recombinant GST or GST-tagged UBASH3B fragments were immobilized on Glutathione Sepharose 4B beads and incubated with cell extracts for 2–3 h at 4 °C under constant rotation in lysis buffer. Beads were then washed extensively with lysis buffer, and bound proteins were eluted by boiling in Laemmli buffer containing β-mercaptoethanol. Samples were analyzed by SDS-PAGE and western blotting using the indicated antibodies.

### TCGA data analysis

Publicly available TCGA tumor datasets (Data ref: The Cancer Genome Atlas Research Network, dbGaP phs000178.v11.p8) were used to assess the relationship between UBASH3B expression and tumor aneuploidy in cancer types corresponding to the cell-line panel analyzed in this study. UBASH3B expression values were compared with published tumor aneuploidy scores, and correlations were calculated using Pearson correlation analysis.

### Surface plasmon resonance (SPR)

All SPR experiments were performed on a Biacore T200 instrument (GE Healthcare) at 25 °C, with the autosampler rack base maintained at 15 °C. Streptavidin-coated SAD 500 L sensor chips and surfactant polysorbate 20 (P20) were sourced from Xantec. The running buffer for immobilization consisted of PBS (10 mM phosphate, 175 mM NaCl, pH 7.4) filtered through a 0.22-μm membrane and supplemented with 0.005% (v/v) P20. Sensor surfaces were conditioned with three 30-second pulses of 10 mM HCl, 50 mM NaOH, and 1 M NaCl, followed by thorough washing. Biotinylated ligands were immobilized to achieve levels between 2500 and 10,000 response units (RU). A biotinylated MBP control was immobilized on one flow cell per chip to monitor non-specific binding and refractive index changes. The kinetics running buffer comprised PBS filtered through a 0.22-μm membrane and supplemented with 0.05% (v/v) P20 and 1% DMSO. Six startup buffer injections were performed to condition the sensor surface before each run. Analytes (0–200 μM, 8–12 concentrations) were injected at 30 μL/min over all flow cells for 120 s (association) followed by 600 s for dissociation. Three concentrations (2.5, 30, and 100 μM) were duplicated in non-consecutive cycles to assess reproducibility. No regeneration step was required, as the dissociation phase allows the signal to return to baseline level. All interactions were analyzed in duplicate across two independent sensor surfaces. Data were processed using a modified double-referencing method (Fournane et al, 2011). Sensorgrams were first corrected for buffer effects using BiaEvaluation 3.2 software (GE Healthcare). Using an in-house Python script, the bulk refractive index changes were then corrected by subtracting the MBP reference signal, considering the following weighted correction factor:

$$\frac{{{MW}}_{{MBP}}}{{{MW}}_{{Lig}}}/\frac{{{Immob}}_{{Lig}}}{{{Immob}}_{{MBP}}}$$

where MW and Immob denote molecular weights and immobilization levels, respectively, for MBP and the ligand. Finally, each double-referenced sensorgram was further normalized by dividing by the theoretical maximum normalized binding signal R_max_, according to:

$${R}_{max }=\frac{{{MW}}_{{analyte}}}{{{MW}}_{{Lig}}}{{Immob}}_{{Lig}}$$

and assuming a 1:1 interaction with a ligand 100% active on the surface (Dobrovodský and Di Primo, 2023). The full process allows to directly compare sensorgrams obtained for different ligand with different immobilization levels and with analytes with different molecular weights. The kinetic signals displayed square profiles, as usually seen for low molecular weight analytes. The plateau during the association phase reached for each concentration, increased with the analyte concentrations, suggesting a direct interaction.

Equilibrium binding signals (*R*_*eq*_) were calculated by averaging a 5-s window before dissociation. Responses were fit to a 1:1 binding isotherm using custom Python scripts. Normalized signals (≤ 60 RU) excluded MBP contributions and enabled direct comparison across ligands and analytes. Square-shaped sensorgrams indicated rapid dissociation, while the absence of concentration-dependent plateaus observed for several analytes confirmed the specific interaction.

### Fluorescence polarization titration

Fluorescence polarization assays were performed in 96-well black polystyrene plates (Costar 3686, Corning, Acton, MA) in a total volume equals to 15 μL. The fluorescence was measured with Envision apparatus (Perkin-Elmer Waltham, MA). The polarization degrees (FP) were measured with an excitation wavelength set at 530 nm and an emission wavelength set at 595 nm. All titrations were performed by adding increasing amounts of protein to a fixed concentration of fluorescent compounds (0.3 μM) in assay buffer (50 mM HEPES, 150 mM KCl, 0.2% Pluronic F-127, pH 7.5). The binding affinity of fluorescent compounds for the proteins, expressed by dissociation constant (Kd = 1/Kass), then was calculated by fitting the sigmoidal dose-dependent FP change as a function of protein concentration using Microsoft Excel solver and the following equations:

1 $$P+{Lf}\Longleftrightarrow {PL}$$

2 $${Kd}=([P]* [{Lf}])/[PL]$$

3 $$[{Pt}]=[P]+[PL]$$

4 $$[{Lt}]=[{Lf}]+[PL]$$

where [P], [*Lf*] et [PL] are, respectively, the equilibrium concentrations of the free protein, of the free ligand and of the free ligand–protein complex. [Pt] is a total protein concentration (X input), [Lt] is a total ligand concentration (constant), and Kd is the dissociation constant (variable to be fitted).

The fraction of ligand bound to protein (Fb) is described by:

5 $${Fb}=[{Lt}]-[{Lf}][{Lt}]$$

Substituting Eqs. (3) and (4) into Eqs. (2) and (5) we obtain:

$${Fb}=({{\rm{Kd}}}+[{{\rm{Lt}}}]+[{{\rm{Pt}}}]-\surd ({({{\rm{Kd}}}+[{{\rm{Lt}}}]+[{{\rm{Pt}}}])}^{2}-4[{{\rm{Lt}}}][{{\rm{Pt}}}]))/2[{Lt}]$$

Anisotropy (*r*) as a function of *Fb* is described as follows:

$$r={Fb}.{rb}+(1-{Fb}).{rf}$$

where *r* is the experimental anisotropy value, *rb* is the anisotropy value of ligand–protein complex, and *rf* is the anisotropy value of free ligand.

### Design of UBASHIN-1 derivatives

Firstly, UBASHIN-3 was obtained by replacing the lissamine rhodamine dye by an adamantyl group, thus removing the fluorescent character of the UBASHIN-1 while keeping its hydrophobic properties. Secondly, we enhanced enzymatic stability of UBASHIN-3 by mutating the two key L-amino acids (Lys and Phe) by the corresponding D-amino acids. Finally, to increase plasma stability, we replaced the adamantyl moieties of UBASHIN-3 by a fluorocarbon chain (FC), leading to UBASHIN-5. This approach was recently applied to prepare metabolically stable peptides targeting GPCRs for the potential treatment of hyponatremia, cardiovascular diseases or multiple sclerosis (Birmpili et al, 2024; Flahault et al, 2021; Girault-Sotias et al, 2025).

### Synthesis of compounds UBASHIN-1, UBASHIN-2, UBASHIN-3, UBASHIN-4, UBASHIN-5 and negative control

#### Reagents

Reagents were obtained from commercial sources and used without any further purification. Fmoc-L-amino acids were purchased from Iris biotech and Sigma-Aldrich. Fmoc-NH-SPOrT (0.57 mmol.g^−1^ resin) resin was prepared as previously described (Bonnet et al, 2008). Overall yields for the solid-phase syntheses were calculated based on the initial loadings provided by the supplier (0.69 mmol.g^−1^).

#### General peptide synthesis

Peptides were synthesized by standard Fmoc solid-phase chemistry starting from Fmoc-NH-SPOrT resin, using DMF as a solvent. The coupling of each amino acid (4 equiv.) was carried out using HATU (3.9 equiv.) and DIEA (12 equiv.) in DMF. Fmoc groups were removed using a 20% v/v solution of piperidine in DMF. Washing steps were performed using DMF, DCM and MeOH.

#### General procedure for the solid-phase click chemistry

Resin-bound alkyne (0.1 mmol) was preswollen in a DMF/piperidine (4/1, v/v) mixture. In two separated vials, CuI (5 equiv.) and 2-azidoethan-1-amine (2 equiv.) were dissolved in DMF/piperidine (4/1, v/v, 2 mL) using ultrasonic bath. Both solutions were mixed altogether and placed again 5 min in an ultrasonic bath. The resulting mixture (4 mL) was added to preswollen resin-bound alkyne. Following shaking for 6 h at RT, the resin was washed successively with 4/1 (by vol) DMF/piperidine (3×), DMF (3×), CH_2_Cl_2_ (3×), CH_2_Cl_2_/MeOH (3×), MeOH (3x), CH_2_Cl_2_ (3×).

#### Peptide acylation

The acylation of deprotected NH_2_ group with fluorocarbon chain (2 equiv.) was performed using HATU (1.9 equiv.) and DIEA (8 equiv.) in DMF. The acylation of deprotected NH_2_ group with adamantane-1-carboxylic acid (4 equiv.) or 4,4,5,5,6,6,7,7,8,8,9,9,10,10,11,11,11-heptadecafluoroundecanoic acid (fluorocarbon chain - FC) was performed using HATU (3.9 equiv.) and DIEA (12 equiv.) in DMF. The mixture was shaken at RT for 45 min. The solution was drained. The resin was washed with DMF, DCM and MeOH and dried in vacuo.

#### Peptide sulfonylation with lissamine rhodamine B sulfonyl chloride

The sulfonylation of deprotected NH_2_ group with Lissamine Rhodamine B Sulfonyl Chloride (2.5 equiv.) was performed using DIEA (12 equiv.) in DCM. Following shaking for 6 h at RT in the dark, the resin was washed successively with DMF, DMF/DIEA (9/1, v/v), THF/H_2_O (2/1, v/v), DCM, DCM/MeOH (1/1, v/v), MeOH (5 times).

#### Peptide cleavage and deprotection

Final peptides were deprotected and cleaved from the resin under acidic conditions (95% TFA, 5% thioanisole, 5% triisopropylsilane) for 3 h with shaking at RT. The resin was removed by vacuum filtration, and the peptides were precipitated with cold diethyl ether (50 mL). The precipitated peptides were washed with cold diethyl ether. Finally, peptides were either freeze-dried or directly purified.

#### Peptide purification

Crude peptides were purified by semi-preparative reversed-phase HPLC chromatography (Gilson PLC2020 System) on a SunFire C18 column (5 μm, 19 × 150 mm) using a linear gradient (5–95% in 40 min, flow-rate of 18 ml.min^−1^) of solvent B (0.1% TFA in MeCN, v/v) in solvent A (0.1% TFA in H_2_O, v/v). Detection was set at 220 and 254 nm. Fractions containing the desired peptide were freeze-dried.

#### RP-HPLC and mass spectrometric analysis

The mass of the purified product was obtained on an Agilent Technologies 6520 Accurate-Mass Q.Tof LC/MS apparatus equipped with a Zorbax SB C18 column (1.8 μm, 2.1 × 50 mm) using electrospray ionization (ESI) and a time-of-flight analyzer (TOF). The purity was assessed by RP-HPLC on a C18 Ascentis Express (2.7 μm, 4.6 mm × 7.5 mm) using a linear gradient (5–100% in 7.39 min, flow rate of 1.6 mL.min^−1^) of solvent B (0.1% TFA in ACN, v/v) in solvent A (0.1% TFA in water, v/v).

**UBASHIN-1** was synthesized following the general procedure starting from Fmoc-NH-SPOrT resin (0.11 mmol). The resin was submitted to click chemistry procedure and sulfonylation with lissamine rhodamine B sulfonyl chloride. Following deprotection and cleavage, the compound was isolated as a pink solid (46.7 mg, 33%); *t*_R_ = 5.44 min; RP-HPLC purity >95%; MS (ESI): calcd for C_57_H_69_ClN_13_O_10_S_2_ [M + H^+^] 1194.43, found 1194.43.

**UBASHIN-2** was synthesized following the general procedure starting from Fmoc-NH-SPOrT resin (0.11 mmol). Following deprotection and cleavage, the compound was isolated as a white solid (19.4 mg, 26%); *t*_R_ = 3.67 min; RP-HPLC purity > 95%; MS (ESI): calcd for C_28_H_35_ClN_7_O_4_ [M + H^+^] 568.23, found 568.19.

**UBASHIN-3** was synthesized following the general procedure starting from Fmoc-NH-SPOrT resin (0.2 mmol). The resin was submitted to click chemistry procedure and acylation with adamantane-1-carboxylic acid. Following deprotection and cleavage, the compound was isolated as a white solid (71.9 mg, 38%); *t*_R_ = 4.36 min; RP-HPLC purity >95%; MS (ESI): calcd for C_41_H_55_ClN_11_O_5_ [M + H^+^] 816.40, found 816.40.

**UBASHIN-4** was synthesized following the general procedure starting from Fmoc-NH-SPOrT resin (0.29 mmol). The resin was submitted to  click chemistry procedure and acylation with adamantane-1-carboxylic acid. Following deprotection and cleavage, the compound was isolated as a white solid. The compound was then treated with Dowex Resin to obtain an acetate salt (97 mg, 37%); *t*_R_ = 4.31 min; RP-HPLC purity >98%; MS (ESI): calcd for C_41_H_55_ClN_11_O_5_ [M + H^+^] 816.40, found 816.40.

**UBASHIN-5** was synthesized following the general procedure starting from Fmoc-NH-SPOrT resin (0.25 mmol). Then, the resin was submitted to click chemistry procedure and acylation with fluorocarbon chain. Following deprotection and cleavage, the compound was isolated as a white solid. The compound was then treated with Dowex resin to obtain an acetate salt (142.8 mg, 48%); *t*_R_ = 5.79 min ; RP-HPLC purity >95%; MS (ESI): calcd for C_41_H_44_ClF_17_N_11_O_5_ [M + H^+^] 1128.28, found 1128.20.

### Plasma stability assay

Ex vivo stability assessments for UBASHIN-3 and UBASHIN-4 were conducted in mouse plasma at 37 °C (Appendix Fig. S1B). Compounds were prepared as 1 mM DMSO stocks, diluted to 10 μM in plasma (400 μL final volume), and incubated at 37 °C. Aliquots (70 μL) were collected at 0, 0.5, 2, 5, and 24 h, mixed with 175 μL ice-cold acetonitrile, and frozen. Positive controls (e.g., procaine) were processed identically. Samples were thawed, vortexed (5 min), sonicated (1 min), and centrifuged (15,000 ×g, 5 min, 10 °C). Supernatants were analyzed using a Shimadzu LC-MS 8030 UHPLC-triple quadrupole system. UBASHIN-4 showed high ex vivo plasma stability, with 81% of the compound remaining after 24 h, whereas UBASHIN-3 was rapidly degraded. In vivo plasma exposure was assessed separately. To improve in vivo performance, UBASHIN-5 was synthesized with an albumin-binding FC chain, increasing its in vivo plasma half-life 8-fold (118 min vs. 15 min for UBASHIN-4).

### Animal experiments

Mice were bred and maintained at the accredited IGBMC/PHENOMIN-ICS animal facility (C-67-218-37), in compliance with French and EU regulations on the use of laboratory animals. Animal experiments were approved by the Com’Eth ethical committee (Comité d’Ethique en Expérimentation Animale n°017, Strasbourg, France) and the French Ministry of Higher Education and Research (APAFIS# 18645-2019012511432750 v3). Mice were housed under controlled temperature, humidity, ventilation, noise and light conditions, with a 12-h light-dark cycle and free access to water and standard rodent chow.

For pilot tolerability studies, female Balb/c mice (8 weeks old) were treated by intraperitoneal injection with Vehicle, UBASHIN-4 or UBASHIN-5 three times per week. Vehicle-treated mice received 150 mM NaCl supplemented with 10% Cremophor. Mice were monitored daily for morphology and behavior, and body weight was measured regularly during the treatment period.

The breast adenocarcinoma cell line MDA-MB-231 (ATCC HTB-26) expressing the luciferase reporter gene luc2 (Photinus pyralis) was generated using the method described before (Molière et al, 2024). Eighteen Balb/c nude mice (6 weeks old) were subcutaneously injected with 10^6 luciferase-expressing MDA-MB-231 cells on day 0 and randomized into three groups of six mice. Mice received intraperitoneal injections of 100 µL of either vehicle (150 mM NaCl, 10% Cremophor), Docetaxel (Fresenius Kabi; 3 mg/kg in 150 mM NaCl), or UBASHIN-4 (5 mg/kg in 150 mM NaCl, 10% Cremophor) on days 2, 7, 10, 14, 17, and 21. Tumor growth was monitored on days 8, 13, 16, 19, and 22 using bioluminescence imaging (BLI). Mice were injected intraperitoneally with 100 μL of D-luciferin (15 mg/mL, XenoLight, PerkinElmer) and anesthetized with isoflurane (induction: 3%, maintenance: 1% in oxygen at 0.5–1 L/min). BLI scans were performed using an IVIS Spectrum Imaging System (PerkinElmer). Images were acquired over 16 min with 1–4 s exposure times and a 12.5 cm field of view. Regions of interest (ROIs) were analyzed to quantify total flux, radiance (light emitted per unit area and solid angle), signal variability, and peak signal intensity. On day 22, animals were euthanized, and tumors were excised, weighed, fixed in cold 4% (w/v) paraformaldehyde (PFA) in PBS and embedded in paraffin.

For the syngeneic colorectal cancer model, female Balb/c mice (8 weeks old) were subcutaneously injected with 10^5^ CT26 (ATCC CRL-2638) mouse colon adenocarcinoma cells on day 0 and randomized into three groups of eight mice. Treatments started on day 3 and were administered every 2 days by intraperitoneal injection. Mice received Vehicle, Docetaxel or UBASHIN-5. Vehicle-treated mice received 150 mM NaCl supplemented with 10% Cremophor, while Docetaxel and UBASHIN-5 were administered at 3 mg/kg. Tumor growth was monitored by caliper measurements, and body weight was monitored regularly during the treatment period. The ethical endpoint was defined as a tumor volume of 2000 mm^3^. The experiment was stopped on day 21, animals were euthanized, and tumors were excised, weighed, fixed and embedded in paraffin.

### Histology and tumor-section immunostaining

Following collection, tumors were fixed overnight in cold 4% (w/v) paraformaldehyde (PFA) in PBS. After removal of the fixative, the tissues were rapidly rinsed in PBS, dehydrated and embedded in paraffin. In all, 5-µm-thick sections were made. For histology, sections were stained with hematoxylin and eosin (H&E). For IHC, sections were processed for antigen retrieval for 20 min in a steamer with Tris-EDTA buffer (pH 9.0) (10 mM Tris base, 1 mM EDTA). They were rinsed in PBS containing 0.1% (v/v) Tween 20 (PBST) (three times for 5 min each) and then incubated in a humidified chamber for 16 h at 4 °C with primary antibodies against Aurora B, Ki67 and pH3S10. After rinsing in PBST (three times for 5 min each), detection of the bound primary antibodies was performed for 1 h at room temperature in a humidified chamber using an Alexa Fluor 647-conjugated donkey anti-rabbit IgG. The sections were then counterstained with 4′,6-diamidino-2-phenylindole (DAPI) diluted at 10 µg/ml in the mounting medium (Citifluor MWL4-88, EMS) to label nuclei. Labelled sections were digitized for quantification using a Zeiss AxioScan7 slide scanner. Representative images shown in the figures were acquired using a Zeiss Apotome system. Apoptotic cells were detected using the In Situ Cell Death Detection Kit (TUNEL assay). Briefly, sections were deparaffinized, treated with Proteinase K diluted in PBS (20 µg/ml), rinsed in PBST (three times for 5 min each) and TUNEL mixture was applied during 1 h in a humidified chamber at 37 °C. Image analysis and segmentation of tissue sections were performed using Ilastik, NDP.view2 and QuPath software.

### Statistics

Statistical analyses were performed using the tests indicated in the figure legends. Data are presented as mean ± SEM unless otherwise stated. The number of independent experiments, cells, fields, spreads or animals analyzed is indicated in the corresponding figure legends. Sample sizes were not predetermined by statistical methods but were based on previous experimental experience and standard practice. For comparisons between two groups, Student’s *t* tests or Welch’s *t* tests were used as indicated. Paired tests were used when measurements were matched across independent experiments. One-tailed tests were used only for pre-specified directional comparisons, as indicated in the figure legends. Unless otherwise stated, all biologically valid samples and acquired data points were included; exclusions were limited to technical failures or predefined quality-control criteria. For animal experiments, outliers were identified using Grubbs’ test and excluded before statistical analysis where indicated. Mice were randomly allocated to treatment groups before treatment initiation, and investigators were not blinded to treatment allocation or analysis. *P* values below 0.05 were considered statistically significant.

## Supplementary information

Appendix

Peer Review File

Movie EV1

Movie EV2

Movie EV3

Movie EV4

Source data Fig. 1

Source data Fig. 2

Source data Fig. 3

Source data Fig. 4

Source data Fig. 5

Source data Fig. 6

Source data Fig. 7

Source data Fig. 8

Source data Fig. 9

Source data Fig. 10

Expanded View Figures

## Data Availability

Custom Python scripts used for SPR data processing and analysis are publicly available at: https://github.com/YvesNomine/SPR_Kinetics_Analysis/. The source data of this paper are collected in the following database record: biostudies:S-SCDT-10_1038-S44321-026-00519-0.
