# Supplementary material for: Pharmacological inhibition of UBASH3B induces mitotic arrest and selectively reduces tumor growth
Source: EMBO Mol Med. 2026 Sep 22;18(11):4260–99. doi: 10.1038/s44321-026-00519-0 (PMC13748553; doi:10.1038/s44321-026-00519-0)
Supplement: Supplementary file 1 [file 44321_2026_519_MOESM17_ESM.pdf]

Expanded View Figures

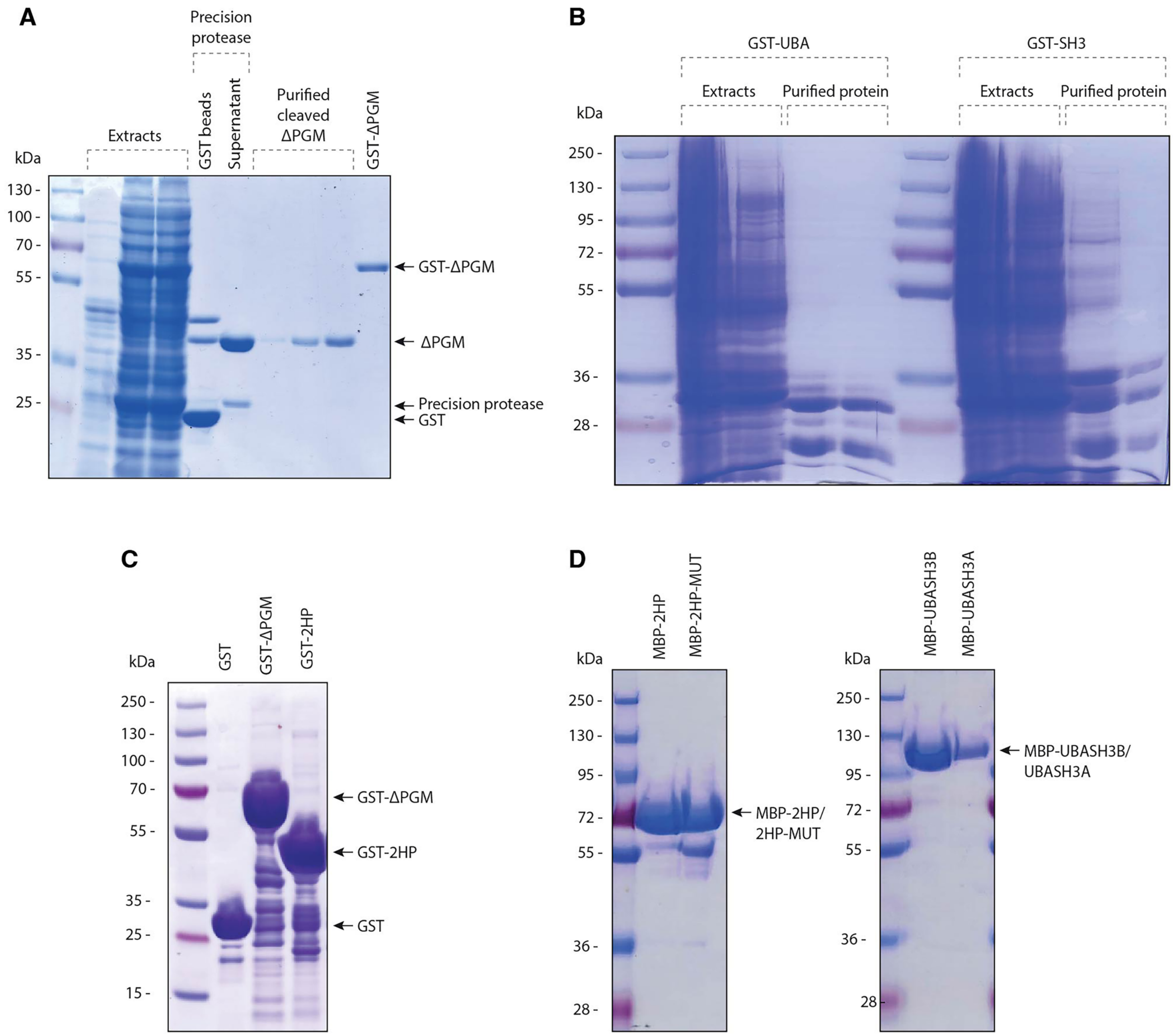

Figure EV1. Purification of UBASH3B and UBASH3A protein fragments used for biochemical assays.

(**A**) Coomassie-stained SDS-PAGE gel showing purification of GST-UBASH3B-ΔPGM from bacterial extracts and cleavage to release the UBASH3B-ΔPGM fragment used for FP assays. Bands corresponding to GST-UBASH3B-ΔPGM, cleaved UBASH3B-ΔPGM, GST and precision protease are indicated. (**B**) Coomassie-stained SDS-PAGE gel showing bacterial extracts and purified GST-UBA and GST-SH3 fragments used for FP assays. (**C**) Coomassie-stained SDS-PAGE gel showing purified GST-UBASH3B-ΔPGM and GST-2HP fragments used for FP assays. (**D**) Coomassie-stained SDS-PAGE gel showing purified MBP-tagged UBASH3B 2HP, UBASH3B 2HP-MUT, UBASH3B-WT and UBASH3A-WT proteins used for SPR analysis.

© The Author(s)

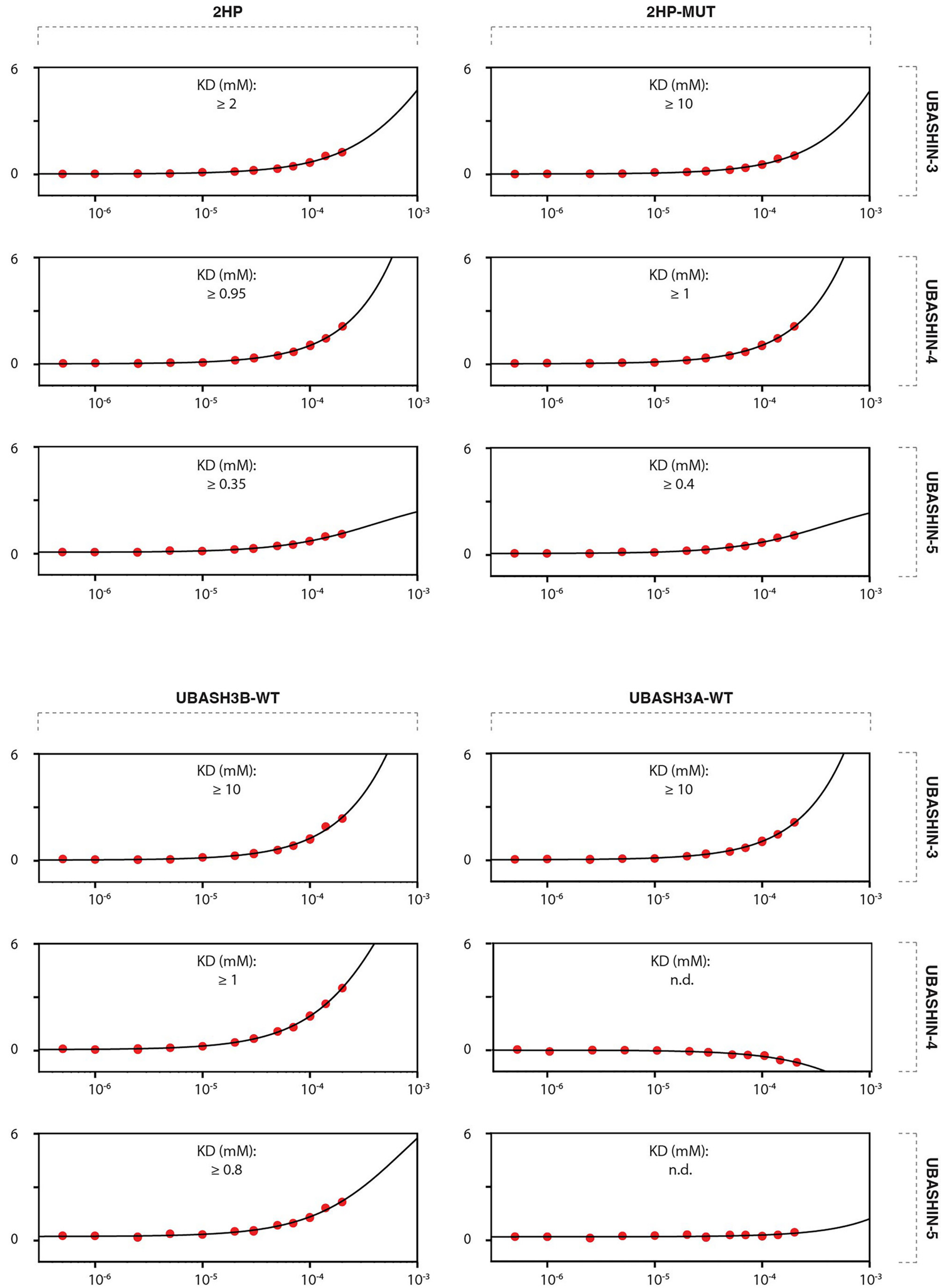
2HP
2HP-MUT
UBASHIN-3
KD (mM): ≥ 2
KD (mM): ≥ 10
UBASHIN-4
KD (mM): ≥ 0.95
KD (mM): ≥ 1
UBASHIN-5
KD (mM): ≥ 0.35
KD (mM): ≥ 0.4
UBASH3B-WT
UBASH3A-WT
UBASHIN-3
KD (mM): ≥ 10
KD (mM): ≥ 10
UBASHIN-4
KD (mM): ≥ 1
KD (mM): n.d.
UBASHIN-5
KD (mM): ≥ 0.8
KD (mM): n.d.
6
0
10-6
10-5
10-4
10-3

 © The Author(s)

◀ **Figure EV2. Steady-state SPR binding analysis of UBASHIN derivatives.**

Steady-state binding curves derived from surface plasmon resonance (SPR) analysis of UBASHIN-3, UBASHIN-4 and UBASHIN-5 binding to purified MBP-tagged UBASH3B 2HP, UBASH3B 2HP-MUT, UBASH3B-WT and UBASH3A-WT proteins. Apparent K_D values are indicated for each protein–compound pair. Because several curves did not reach a clear saturation plateau within the tested concentration range, Kd values should be interpreted as apparent affinity estimates used for relative comparison between constructs, rather than precise equilibrium constants. n.d. non-determined values.

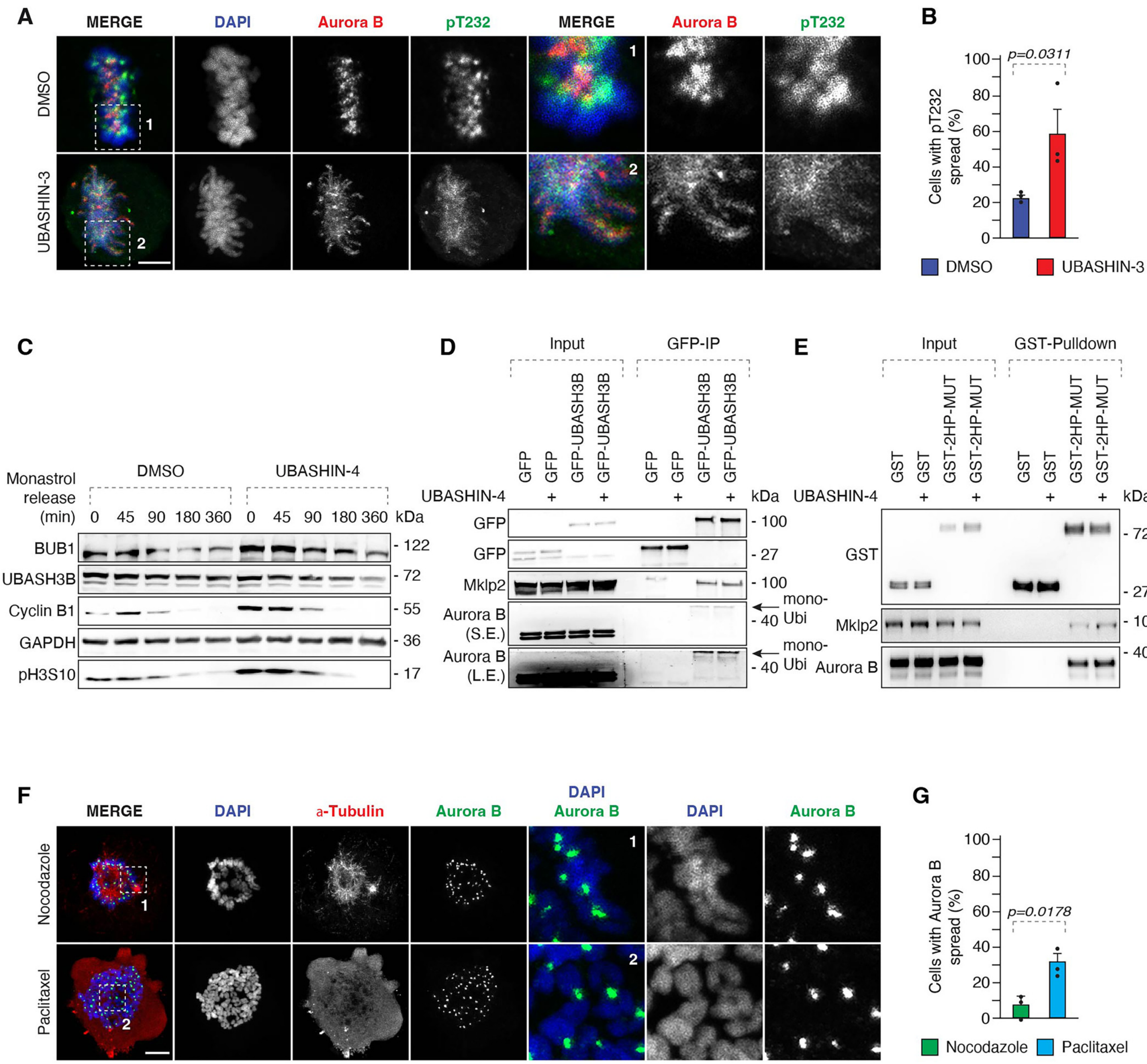

Figure EV3. UBASHIN alters Aurora B localization and activity without disrupting UBASH3B-Aurora B/Mklp2 interactions.

(**A**) Representative IF images of MDA-MB-231 cells treated with DMSO or UBASHIN-3 and stained for Aurora B (red), phosphorylated Aurora B at T232 (green) and DNA (DAPI, blue). Zoomed regions highlight Aurora B and pT232 localization on mitotic chromosomes. Scale bar, 5 µm. (**B**) Quantification of cells displaying phosphorylated Aurora B T232 signal spreading along chromosome arms. Data are shown as mean ± SEM from three independent experiments; individual data points are shown. *P* values were calculated using a one-tailed unpaired Student's *t* test. (**C**) WB analysis of HeLa cells treated with DMSO or UBASHIN-3 following release from monastrol-induced prometaphase arrest. Cells were analyzed with antibodies against Bub1, UBASH3B, Cyclin B1, pH3S10 and GAPDH (loading control). (**D**) GFP immunoprecipitation analysis of UBASH3B interaction with Aurora B and Mklp2 in mitotically synchronized HeLa cells treated with DMSO or UBASHIN-4. (**E**) In vitro binding analysis of Aurora B and Mklp2 interaction with GST or GST-2HP-MUT in the presence or absence of UBASHIN-4. (**F**) Representative IF images of HeLa cells treated with Nocodazole or Paclitaxel and stained for Aurora B (green), α-tubulin (red) and DNA (DAPI, blue). Zoomed regions highlight Aurora B localization. Scale bar, 7.5 µm. (**G**) Quantification of cells displaying Aurora B spreading along chromosome arms after Nocodazole or Paclitaxel treatment. Data are shown as mean ± SEM from three independent experiments; individual data points are shown. *P* values were calculated using two-tailed Welch's *t* tests.

 © The Author(s)

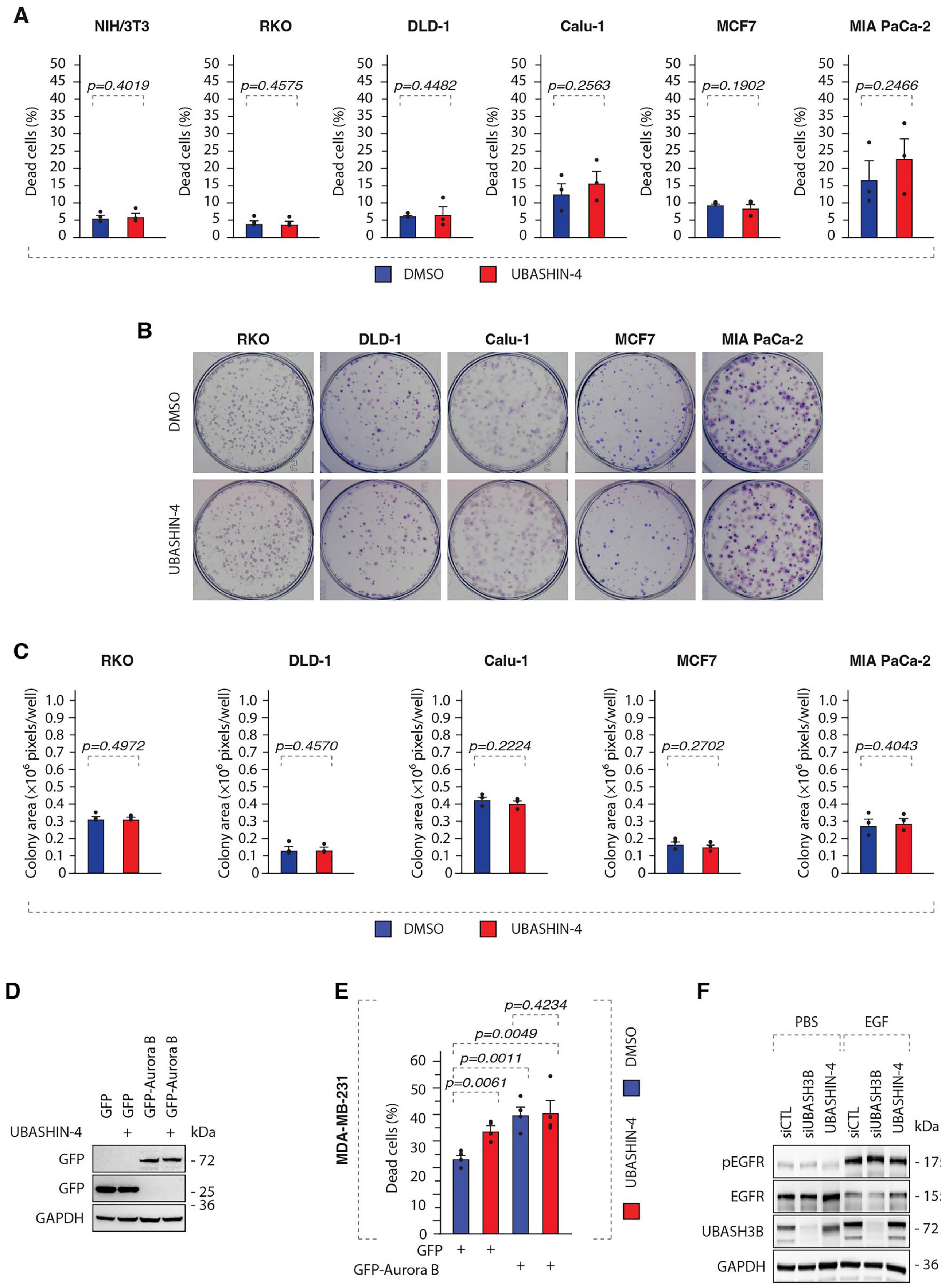
A
NIH/3T3
RKO
DLD-1
Calu-1
MCF7
MIA PaCa-2
Dead cells (%)
p=0.4019
p=0.4575
p=0.4482
p=0.2563
p=0.1902
p=0.2466
DMSO
UBASHIN-4
B
RKO
DLD-1
Calu-1
MCF7
MIA PaCa-2
DMSO
UBASHIN-4
C
RKO
DLD-1
Calu-1
MCF7
MIA PaCa-2
Colony area (×10^6 pixels/well)
p=0.4972
p=0.4570
p=0.2224
p=0.2702
p=0.4043
DMSO
UBASHIN-4
D
GFP
GFP
GFP-Aurora B
GFP-Aurora B
UBASHIN-4
kDa
GFP
GFP
GAPDH
- 72
- 25
- 36
E
MDA-MB-231
Dead cells (%)
p=0.4234
p=0.0049
p=0.0011
p=0.0061
GFP
GFP-Aurora B
DMSO
UBASHIN-4
F
PBS
EGF
siCTL
siUBASH3B
UBASHIN-4
siCTL
siUBASH3B
UBASHIN-4
kDa
pEGFR
EGFR
UBASH3B
GAPDH
- 175
- 155
- 72
- 36

© The Author(s)

Figure EV4. Sensitivity of cancer cells to UBASHIN is not associated with Aurora B abundance or EGFR signaling.

(**A**) Quantification of cell death in NIH/3T3, RKO, DLD-1, CALU-1, MCF7 and MIA PACA-2 cells treated with DMSO or UBASHIN-4, assessed by Trypan Blue Exclusion (TBE). Data are shown as mean ± SEM from three independent experiments; individual data points are shown. *P* values were calculated using one-tailed unpaired Student's *t* tests. (**B**) Representative colony formation assay images of RKO, DLD-1, CALU-1, MCF7 and MIA PACA-2 cells treated with DMSO or UBASHIN-4. Colonies were stained with crystal violet. (**C**) Quantification of total colony-positive area, expressed as $\times 10^6$ pixels/well, from the colony formation assays shown in (**B**). Data are shown as mean ± SEM from three independent experiments; individual data points are shown. *P* values were calculated using one-tailed unpaired Student's *t* tests. (**D**) WB analysis of GFP-Aurora B overexpression in MDA-MB-231 cells treated with DMSO or UBASHIN-4. GAPDH was used as a loading control. (**E**) Quantification of cell death in MDA-MB-231 cells expressing GFP or GFP-Aurora B and treated with DMSO or UBASHIN-4, assessed by TBE. Data are shown as mean ± SEM from four independent experiments; individual data points are shown. *P* values were calculated using one-tailed unpaired Student's *t* tests. (**F**) WB analysis of EGFR phosphorylation in MDA-MB-231 cells treated with DMSO, UBASHIN-4 or UBASH3B siRNA in the presence or absence of EGF stimulation. Cells were analyzed with antibodies against pEGFR, EGFR, UBASH3B and GAPDH.

 © The Author(s)

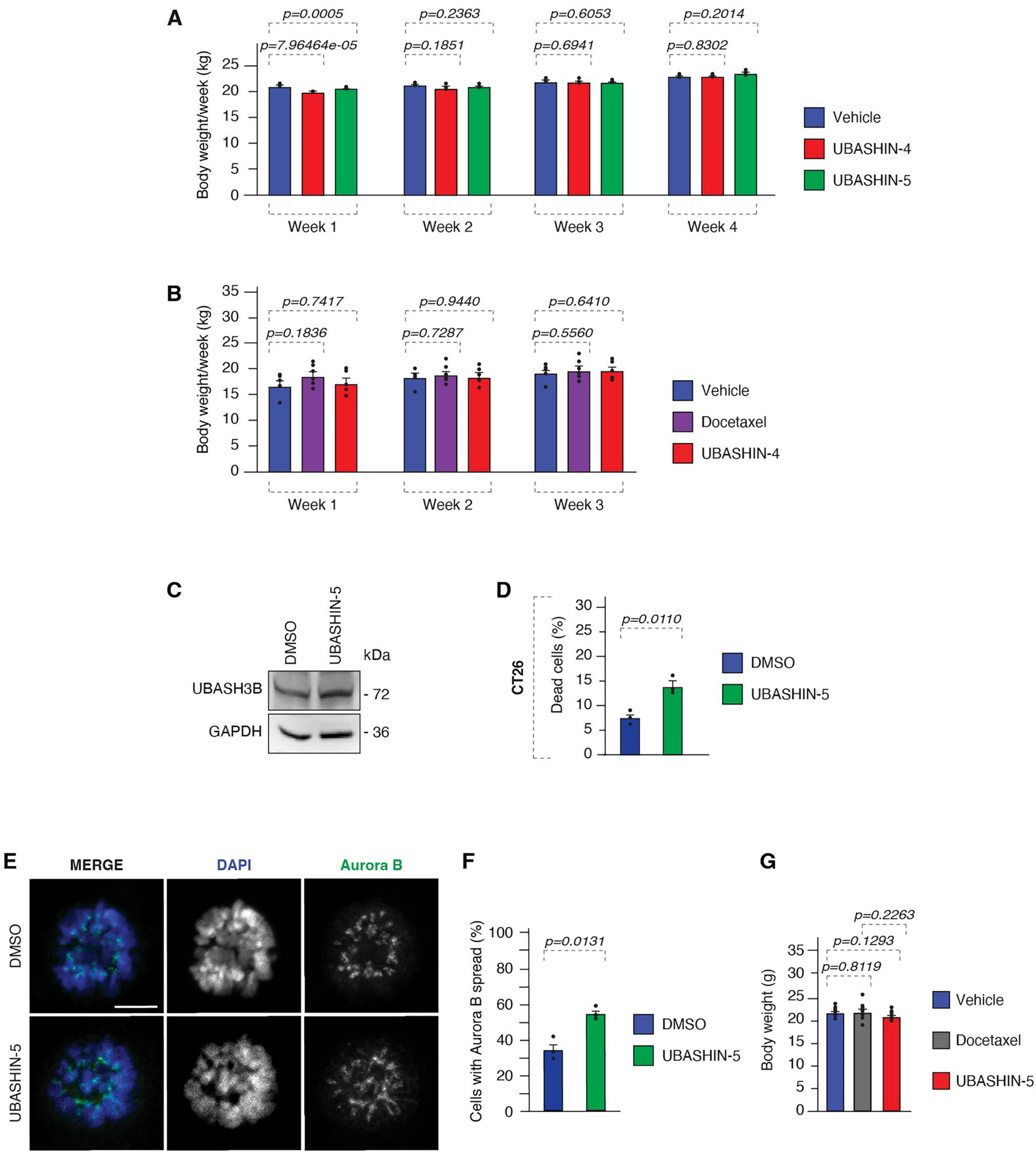
A
p=0.0005
p=7.96464e-05
p=0.2363
p=0.1851
p=0.6053
p=0.6941
p=0.2014
p=0.8302
Body weight/week (kg)
25
20
15
10
5
0
Week 1
Week 2
Week 3
Week 4
Vehicle
UBASHIN-4
UBASHIN-5
B
p=0.7417
p=0.1836
p=0.9440
p=0.7287
p=0.6410
p=0.5560
Body weight/week (kg)
35
30
25
20
15
10
5
0
Week 1
Week 2
Week 3
Vehicle
Docetaxel
UBASHIN-4
C
DMSO
UBASHIN-5
kDa
UBASH3B
- 72
GAPDH
- 36
D
CT26
Dead cells (%)
30
25
20
15
10
5
0
p=0.0110
DMSO
UBASHIN-5
E
MERGE
DAPI
Aurora B
DMSO
UBASHIN-5
F
Cells with Aurora B spread (%)
100
80
60
40
20
0
p=0.0131
DMSO
UBASHIN-5
G
p=0.2263
p=0.1293
p=0.8119
Body weight (g)
35
30
25
20
15
10
5
0
Vehicle
Docetaxel
UBASHIN-5

Figure EV5. UBASHIN tolerability in vivo and CT26 cellular response to UBASH3B inhibition.

(**A**) Body weight measurements of WT female Balb/c mice treated with vehicle, UBASHIN-4 or UBASHIN-5 during the pilot in vivo tolerability study. Data are shown as mean ± SEM from $n = 3$ mice per treatment group. *P* values were calculated using two-tailed Welch's *t* tests. (**B**) Body weight measurements of mice from the breast cancer xenograft experiment treated with vehicle, docetaxel or UBASHIN-4. Data are shown as mean ± SEM from $n = 5$ vehicle-treated, $n = 6$ docetaxel-treated and $n = 5$ UBASHIN-4-treated animals included in the final analysis; *P* values were calculated using two-tailed Welch's *t* tests. (**C**) WB analysis of UBASH3B protein levels in CT26 cells treated with DMSO or UBASHIN-5. GAPDH was used as a loading control. (**D**) Quantification of cell death in CT26 cells treated with DMSO or UBASHIN-5, assessed by TBE. Data are shown as mean ± SEM from three independent experiments; individual data points are shown. *P* values were calculated using a two-tailed Welch's *t* test. (**E**) Representative IF images of CT26 cells treated with DMSO or UBASHIN-5 and stained for Aurora B (green) and DNA (blue, DAPI). Scale bar, 5 µm. (**F**) Quantification of cells displaying Aurora B spreading along chromosome arms under the conditions shown in (**E**). Data are shown as mean ± SEM from three independent experiments; individual data points are shown. *P* values were calculated using a two-tailed Welch's *t* test. (**G**) Body weight measurements of mice from the syngeneic colorectal cancer model treated with vehicle, docetaxel or UBASHIN-5. Data are shown as mean ± SEM from $n = 8$ mice per group; individual data points are shown. *P* values were calculated using two-tailed Welch's *t* tests.

 © The Author(s)
