# Supplementary material for: Pharmacological inhibition of UBASH3B induces mitotic arrest and selectively reduces tumor growth
Source: EMBO Mol Med. 2026 Sep 22;18(11):4260–99. doi: 10.1038/s44321-026-00519-0 (PMC13748553; doi:10.1038/s44321-026-00519-0)
Supplement: Supplementary file 2 [file 44321_2026_519_MOESM1_ESM.pdf]

Appendix

Title: Pharmacological inhibition of UBASH3B induces mitotic arrest and selectively reduces tumor growth

Authors: Evanthia Pangou[1, 2, 3, 4, # *], Sushil Awal[1, 2, 3, 4, 5 #], Mathieu Meode[1, 2, 3, 4], Charlotte Kleiss[1, 2, 3, 4], Mehdi Cloarec[1, 2, 3, 4], Erwan Grandgirard[1, 2, 3, 4], Karl Vivot[1, 2, 3, 4], Lucile Guerber[1, 2, 3, 4], Ksenia Krupina[1, 2, 3, 4, 6], Katerina Jerabkova-Roda[1, 2, 3, 4, 7], Paulo J. da Costa[8], Fabien Alpy[1, 2, 3, 4], Arthur Martinet[1, 2, 3, 4], Massimo Lodi[1, 2, 3, 4], Alexandra Cousido-Siah[1, 2, 3, 4], Olivia Wendling[9], Romain Hany[10], Stéphanie Riché[11], François Daubeuf[10], Patrick Gizzi[10], Pascal Villa[10], Yves Nominé[1, 2, 3, 4], Catherine Tomasetto[1, 2, 3, 4], Dominique Bonnet[11] and Izabela Sumara[1, 2, 3, 4 *]

Table of Contents

Appendix Figure S1 Page 2-3

Appendix Figure S2 Page 4-5

A

Name	Compound	Structure	Chemical Formula	Molecular weight (g/mol)
**Negative Control**	SR-12-40	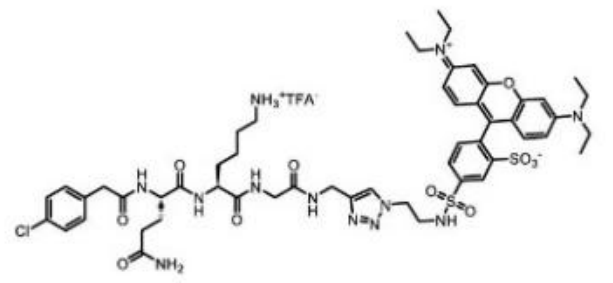	C55H68ClF3N12O13S2	**1260.41**
**UBASHIN-1**	FD-E09	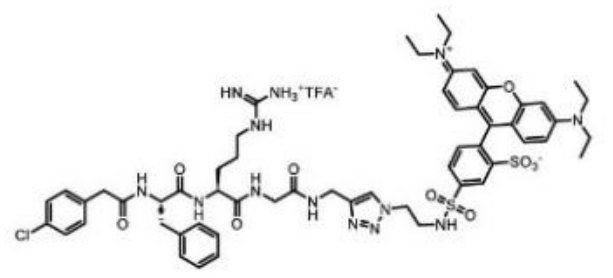	C59H69ClF3N13O12S2	**1307.42**
**UBASHIN-2**	LIT-12-36	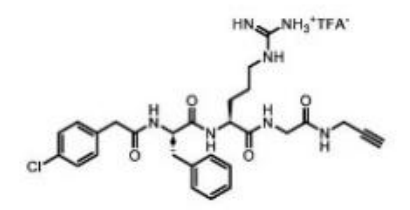	C28H55ClF3N11O7	**750.25**
**UBASHIN-3**	LIT-12-38	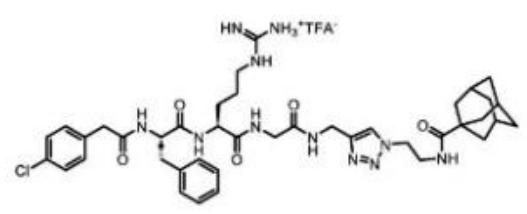	C43H55ClF3N11O7	**929.39**
**UBASHIN-4**	LIT-13-108	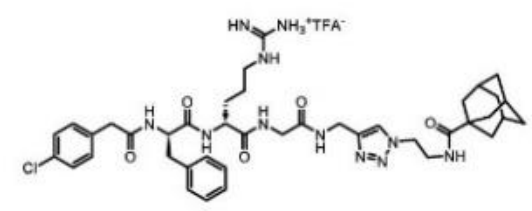	C43H55ClF3N11O7	**929.39**
**UBASHIN-5**	LIT-14-132	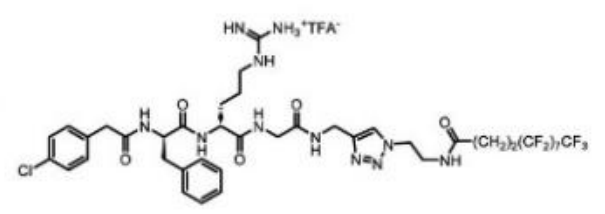	C47H47ClF17N11O7	**1187.31**

B

Name	Compound	Ex vivo half-life in plasma (min)	In vivo half-life in plasma (min)
**UBASHIN-3**	LIT-12-38	12	non-defined
**UBASHIN-4**	LIT-13-108	1440 (81%)	15
**UBASHIN-5**	LIT-14-132	non-defined	118

Appendix Figure S1. Chemical structures and plasma stability of UBASHIN derivatives. **(A)** Summary of the compounds used in this study, including the negative control SR-12-40 and the UBASHIN series (UBASHIN-1, UBASHIN-2, UBASHIN-3, UBASHIN-4 and UBASHIN-5). The compound names, chemical structures, molecular formulas and molecular weights are shown. **(B)** *Ex vivo* plasma stability and *in vivo* plasma half-life of selected UBASHIN derivatives. For *ex vivo* plasma stability, UBASHIN-3 is reported as the calculated half-life, whereas UBASHIN-4 is reported as the percentage of compound remaining after 24 h because its half-life was not reached during the assay. *In vivo* half-lives were determined separately. n.d., not determined.

A

Response	Cell Line	Cancer Type	Approximate Chromosome Number	ATCC No
Responsive	A549	Human Lung Carcinoma	66	CCL-185
Responsive	HCT116	Human Colon Adenocarcinoma	45-46	CCL-247
Responsive	MDA-MB-231	Human Breast Adenocarcinoma	64	HTB-26
Responsive	PANC-1	Human Pancreatic Adenocarcinoma	63	CRL-1469
Responsive	CT26	Mouse Colon Adenocarcinoma	65-70	CRL-2638
Non-Responsive	Calu-1	Human Lung Carcinoma	66-69	HTB-54
Non-Responsive	DLD-1	Human Colon Adenocarcinoma	46	CCL-221
Non-Responsive	MCF7	Human Breast Adenocarcinoma	82	HTB-22
Non-Responsive	MIA PaCa-2	Human Pancreatic Adenocarcinoma	61-63	CRL-1420
Non-Responsive	RKO	Human Colon Carcinoma	45	CRL-2577

B

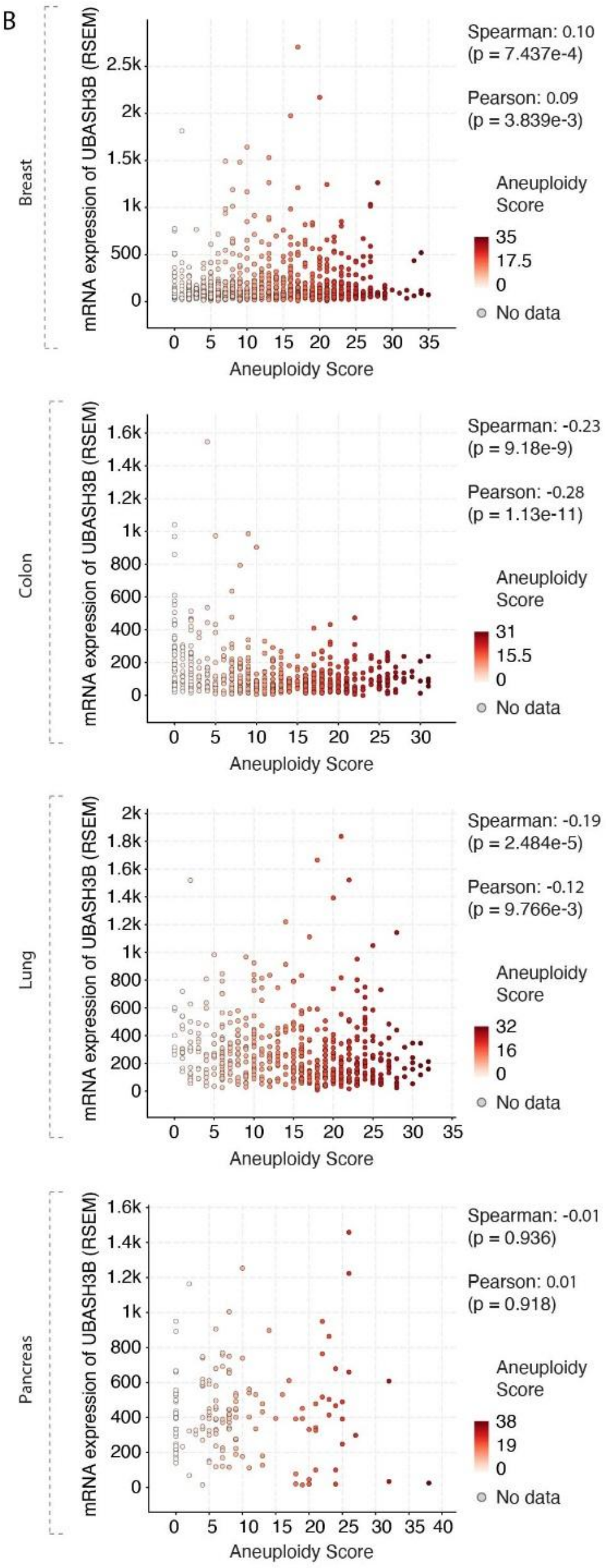

Appendix Figure S2. Cancer cell lines tested for UBASHIN sensitivity and TCGA analysis of UBASH3B expression and aneuploidy.

(A) Overview of cancer cell lines evaluated for their response to UBASH3B inhibition. Cell lines are grouped as UBASHIN-responsive or non-responsive, as defined in the cellular assays. The table indicates cancer type, approximate chromosome number and ATCC reference number. ATCC, American Type Culture Collection. **(B)** Analysis of UBASH3B mRNA expression and aneuploidy score in breast, colon, lung and pancreatic cancer cohorts from The Cancer Genome Atlas (TCGA). Spearman and Pearson correlation coefficients and corresponding P values are indicated for each cancer type. RSEM, RNA-Seq by Expectation-Maximization.
