# Supplementary material for: Pharmacological inhibition of UBASH3B induces mitotic arrest and selectively reduces tumor growth
Source: EMBO Mol Med. 2026 Sep 22;18(11):4260–99. doi: 10.1038/s44321-026-00519-0 (PMC13748553; doi:10.1038/s44321-026-00519-0)
Supplement: Supplementary file 3 [file 44321_2026_519_MOESM2_ESM.pdf]

EMBO Molecular Medicine

Pharmacological inhibition of UBASH3B potentiates the mitotic checkpoint and reduces tumor growth.

Evanthia Pangou, Sushil Awal, Mathieu Meode, Charlotte Kleiss, Mehdi Cloarec, Erwan Grandgirard, Karl Vivot, Lucile Guerber, Ksenia Krupina, Katerina jerabkova-Roda, Paulo Costa, Fabien Alpy, Arthur Martinet, Massimo Lodi, Alexandra Cousido-Siah, Olivia Wendling, Romain Hany, Stéphanie Riché, François Daubeuf, Patrick Gizzi, Pascal Villa, Yves Nominé, Catherine Tomasetto, Dominique Bonnet, and Izabela Sumara

Corresponding authors: Izabela Sumara (sumara@igbmc.fr) , Evanthia Pangou (evapangou@gmail.com)

Review Timeline:

Submission Date:	8th Oct 25
Editorial Decision:	5th Nov 25
Revision Received:	30th Jun 26
Editorial Decision:	27th Jul 26
Revision Received:	19th Aug 26
Accepted:	26th Aug 26

Editor: Lise Roth

Transaction Report:

(Note: With the exception of the correction of typographical or spelling errors that could be a source of ambiguity, letters and reports are not edited. Depending on transfer agreements, referee reports obtained elsewhere may or may not be included in this compilation. Referee reports are anonymous unless the Referee chooses to sign their reports.)

5th Nov 2025

Dear Dr. Sumara,

Thank you for submitting your manuscript to EMBO Molecular Medicine. We have now received feedback from the three reviewers who agreed to evaluate it. As you will see from the reports below, while the reviewers acknowledge the interest and robustness of the findings, they also suggest ways in which the manuscript could be improved.
If you feel you can address the reviewers' comments satisfactorily, you may wish to submit a revised version of your manuscript. Please note that additional experiments in GEMM or carcinogenesis models will NOT be required for the manuscript to be considered further, but this limitation should be discussed in the text.

Addressing the reviewers' concerns in full will be necessary for further considering the manuscript in our journal, and acceptance of the manuscript will entail a second round of review. EMBO Molecular Medicine encourages a single round of revision only and therefore, acceptance or rejection of the manuscript will depend on the completeness of your responses included in the next, final version of the manuscript. For this reason, and to save you from any frustrations in the end, I would strongly advise against returning an incomplete revision.

If you would like to discuss further the points raised by the referees, I am available to do so via email or video. Let me know if you are interested in this option.

We are expecting your revised manuscript within three months, if you anticipate any delay, please contact us.

When submitting your revised manuscript, please carefully review the instructions that follow below. We perform an initial quality control of all revised manuscripts before re-review; failure to include requested items will delay the evaluation of your revision.

We require:

1) A .docx formatted version of the manuscript text (including legends for main figures, EV figures and tables). Please make sure that the changes are highlighted to be clearly visible.

2) Individual production quality figure files as .eps, .tif, .jpg (one file per figure). For guidance, download the 'Figure Guide PDF' (https://www.embopress.org/page/journal/17574684/authorguide#figureformat).

3) At EMBO Press we ask authors to provide source data for the main manuscript figures. You will receive a separate email with instructions for providing source data with your revised manuscript, including how to upload and organize the files.

Additional information on source data and instruction on how to label the files are available

4) A .docx formatted letter INCLUDING the reviewers' reports and your detailed point-by-point responses to their comments. As part of the EMBO Press transparent editorial process, the point-by-point response is part of the Review Process File (RPF), which will be published alongside your paper.

5) A complete author checklist, which you can download from our author guidelines (https://www.embopress.org/page/journal/17574684/authorguide#submissionofrevisions). Please insert information in the checklist that is also reflected in the manuscript. The completed author checklist will also be part of the RPF.

6) All Materials and Methods need to be described in the main text using our 'Structured Methods' format. According to this format, the Methods section includes a Reagents and Tools Table (listing key reagents, experimental models, software and relevant equipment and including their sources and relevant identifiers) followed by a Methods and Protocols section describing the methods, ideally using a step-by-step protocol format. The aim is to facilitate adoption of the methodologies across labs. Please download and fill our Reagents and Tools Table template (.docx), which you can find in our author guidelines: https://www.embopress.org/page/journal/14693178/authorguide#structuredmethods.
When submitting your revised manuscript, please do not include the Reagents and Tools Table in the Methods section of the manuscript but upload it as a separate file choosing the file type "Reagent Table".

7) Please note that all corresponding authors are required to supply an ORCID ID for their name upon submission of a revised manuscript.

8) It is mandatory to include a 'Data Availability' section after the Materials and Methods. Before submitting your revision, primary datasets produced in this study need to be deposited in an appropriate public database, and the accession numbers and database listed under 'Data Availability'. Please remember to provide a reviewer password if the datasets are not yet public (see

https://www.embopress.org/page/journal/17574684/authorguide#dataavailability).

In case you have no data that requires deposition in a public database, please state so in this section. Note that the Data Availability Section is restricted to new primary data that are part of this study.

9) For data quantification: please specify the name of the statistical test used to generate error bars and P values, the number (n) of independent experiments (specify technical or biological replicates) underlying each data point and the test used to calculate p-values in each figure legend. The figure legends should contain a basic description of n, P and the test applied. Graphs must include a description of the bars and the error bars (s.d., s.e.m.). Please provide exact p values.

10) Our journal encourages inclusion of *data citations in the reference list* to directly cite datasets that were re-used and obtained from public databases. Data citations in the article text are distinct from normal bibliographical citations and should directly link to the database records from which the data can be accessed. In the main text, data citations are formatted as follows: "Data ref: Smith et al, 2001" or "Data ref: NCBI Sequence Read Archive PRJNA342805, 2017". In the Reference list, data citations must be labeled with "[DATASET]". A data reference must provide the database name, accession number/identifiers and a resolvable link to the landing page from which the data can be accessed at the end of the reference. Further instructions are available at .

11) We replaced Supplementary Information with Expanded View (EV) Figures and Tables that are collapsible/expandable online. EV Figures should be cited as 'Figure EV1, Figure EV2" etc... in the text and their respective legends should be included in the main text after the legends of regular figures.
- For the figures that you do NOT wish to display as Expanded View figures, they should be bundled together with their legends in a single PDF file called *Appendix*, which should start with a short Table of Content. Appendix figures should be referred to in the main text as: "Appendix Figure S1, Appendix Figure S2" etc.
- Additional Tables/Datasets should be labeled and referred to as Table EV1, Dataset EV1, etc. Legends have to be provided in a separate tab in case of .xls files. Alternatively, the legend can be supplied as a separate text file (README) and zipped together with the Table/Dataset file.
See detailed instructions here:

12) The paper explained: EMBO Molecular Medicine articles are accompanied by a summary of the articles to emphasize the major findings in the paper and their medical implications for the non-specialist reader. Please provide a draft summary of your article highlighting
- the medical issue you are addressing,
- the results obtained and
- their clinical impact.
This may be edited to ensure that readers understand the significance and context of the research. Please refer to any of our published articles for an example.

13) Author contributions: CRediT has replaced the traditional author contributions section because it offers a systematic machine readable author contributions format that allows for more effective research assessment. Please remove the Authors Contributions from the manuscript and use the free text boxes beneath each contributing author's name in our system to add specific details on the author's contribution. More information is available in our guide to authors.

14) Disclosure statement and competing interests: We updated our journal's competing interests policy in January 2022 and request authors to consider both actual and perceived competing interests. Please review the policy https://www.embopress.org/competing-interests and update your competing interests if necessary.

15) Every published paper now includes a 'Synopsis' to further enhance discoverability. Synopses are displayed on the journal webpage and are freely accessible to all readers. They include a short stand first (maximum of 300 characters, including space) as well as 2-5 one-sentences bullet points that summarizes the paper. Please write the bullet points to summarize the key NEW findings. They should be designed to be complementary to the abstract - i.e. not repeat the same text. We encourage inclusion of key acronyms and quantitative information (maximum of 30 words / bullet point). Please use the passive voice. Please attach these in a separate file or send them by email, we will incorporate them accordingly.

Please also suggest a visual abstract to illustrate your article as a PNG file 550 px wide x 300-600 px high. A cropped portion of this image will serve as thumbnail for the table of content on our webpage.

16) As part of the EMBO Publications transparent editorial process initiative (see our Editorial at http://embomolmed.embopress.org/content/2/9/329), EMBO Molecular Medicine will publish online a Review Process File (RPF) to accompany accepted manuscripts.
In the event of acceptance, this file will be published in conjunction with your paper and will include the anonymous referee reports, your point-by-point response and all pertinent correspondence relating to the manuscript. Let us know whether you agree with the publication of the RPF and as here, if you want to remove or not any figures from it prior to publication.

Please note that the Authors checklist will be published at the end of the RPF.

EMBO Molecular Medicine has a "scooping protection" policy, whereby similar findings that are published by others during review or revision are not a criterion for rejection. Should you decide to submit a revised version, I do ask that you get in touch after three months if you have not completed it, to update us on the status.

I look forward to receiving your revised manuscript.

Yours sincerely,

Lise Roth

Lise Roth, PhD
Senior Editor
EMBO Molecular Medicine

***** Reviewer's comments *****

Referee #1 (Comments on Novelty/Model System for Author):

A GEMM or carcinogenesis mouse model would be much better but may be beyond the scope of this manuscript.

Referee #1 (Remarks for Author):

Cancer cell face unprecedented stress in mitosis due to defects in chromosome segregation and therefore have evolved to survive these challenges. One of the potential approaches is to push cancer cells over the brink by increasing mitotic stress, which then could lead to cell death but would have little effect on normal cells. The authors have taken advantage of this approach and have developed a chemical inhibitor of UBASH3B, which controls Aurora B localization and therefore the spindle assembly checkpoint. This inhibitor, UBASHIN, phenocopies UBASH3B deletion, leads to cancer cell death while not affecting normal (non-cancerous) cells. This inhibitor also affects cancer growth in vivo (mice).
This is a high-quality study presenting interesting results potentially paving the way for a less toxic form to replace the standard cancer therapy Paclitaxel. This manuscript is well-written and the experiments are done with high standards including all necessary controls.
There are minor issues that need to be addressed:

1. In Figures 4,5,7,8, the authors do not label the first row with "merge", which is most common, but list all the antibodies. This is confusing.
2. Line 142: "GFP-UBASH3B WT was enriched at the spindle, as" this description does not completely match what the reviewer sees in Figure 1h and this should be improved.
3. Line 161, 196: "FB detection, FPA experiments" these abbreviations have not been introduced.
4. Line 175: "derivatives UBASHINLIT-1238 and UBASHINLIT-1236, along" the names of the derivatives is confusing and could be improved.
5. Line 239: "prevents SAC satisfaction" please rephrase.
6. Line 289,290: "displayed minimal sensitivity to the inhibitor, with" As the authors know, the sensitivity of cells to drugs is dose dependent. Have the authors used a wide range of concentrations of UBASHIN? It would be good to know up to which concentration these cells are not sensitive to UBASHIN.
7. Figure 7 and 8 could be fused since in both cases they are xenograft models. A GEMM or carcinogenesis mouse model would be much better but may be beyond the scope of this manuscript.

Referee #2 (Comments on Novelty/Model System for Author):

The work presents here is novel, has high medical impact and good technical quality

Referee #2 (Remarks for Author):

The manuscript by Pangou et al. addresses an important topic in cancer research, aiming to develop new therapeutic strategies to target aggressive cancers characterized by aneuploidy and chromosome instability.

The authors focus on UBASH3B protein, a regulator of mitotic kinase Aurora B. Building on their previous work on the UBASH3B protein, the authors identify a druggable region of the protein (2HP) and apply a comprehensive set of approaches - including molecular cloning, biochemistry, chemistry, cell biology, live cell imaging and in vivo studies - to identify a new compound termed UBASHIN, that specifically inhibits UBASH3B.
The manuscript reports a complete translational pipeline, progressing from compound discovery and mechanistic characterization to therapeutic evaluation in vitro and in pre-clinical models.
The manuscript is well-written and easy to read, the experiments are carefully performed and the results are novel, compelling and convincing.

However, several points can be raised, particularly concerning the relevance of these findings for cancer patients :

* Authors should investigate whether high vs low UBASH3B levels are associated with CIN and/or aneuploidy in cancer patients, using publicly available transcriptomic databases.

* Authors should perform chromosome spread analysis to measure ploidy status of cancer cells following depletion of UBASH3B and upon overexpression of full-length UBASH3B and its mutants.

*How do authors explain that HCT116 cells are responsive to UBASHIN, given that these cells are nearly diploid and genetically stable?

* The question can be raised whether UBASHIN treatment may benefit to a larger panel of cancer patients. More particularly, is UBASHIN active on cancer cells in which aneuploid/CIN has been induced independently of UBASH3B, by means of centrosome amplification (Plk4), MPS1 inhibition (reversin) or other ways.

*Is UBASHIN also active on cancer cells overexpressing Aurora B, considering that elevated expression of this mitotic kinase is associated with cancer aggressiveness ?

*Would patients benefit from combining UBASHIN and paclitaxel? Authors should analyze the effects of combining both compounds on cancer cells that respond, and on those that do not respond - to UBASHIN alone.

Minor points
* What is the mechanism that the authors do favor to explain UBASHIN-induced mislocalization of UBASH3B from the mitotic spindle?

*Fig. 4j is mislabeled and appears as panel g in the Figure

Referee #3 (Remarks for Author):

Pangour/Sumara et al Embo Molecular Medicine

The team previously discovered that UBASH3B binds mono-ubiquitinated Aurora B and that this promotes binding of Aurora B to the mitotic spindle. Here, they identify small molecules that bind UBASH3B via the 2HP domain. These drugs delocalise Aurora B from the spindle, disrupt mitotic chromosome alignment, are toxic in a subset of cancer cell lines and can suppress tumour growth in vivo. Overall the data is convincing but there are a few specific experiments that would help support their conclusions. My main criticism however is that the story is very difficult to follow - the reader has to work very hard to track the different mutants, drugs and read the figures. These issues can be addressed with changes to the presentation. If the authors were to tighten up the narrative and presentation the story would be much more accessible.

1. Throughout there is a lot of focus on the SAC. However, the thinking is often confused and/or confusing, so much so that it becomes a distraction. For example, the second sentence of the Introduction states that the SAC "oversees" various aspect of mitosis. This is not a correct description of the checkpoint - the SAC is constitutively active in mitosis until all KTs are stably attached to MTs. Yes, this is predicated on spindle assembly and chromosome alignment, but the SAC does not "oversee" these processes. Much of the next paragraph is also very vague - the SAC being "weaker" in many cancer cells (line 59) is a vague statement, citing on a 20-year-old review. The use of the word mitotic "catastrophe" is not helpful in this context. What are the regulators vs core components of the SAC? Later in the manuscript the authors set out to measure SAC "strength" (line 299). Again, this is a rather meaningless concept in the context of the experiment performed. The idea that UBASH3B is a negative regulator of the SAC is also not informative (line 74) in that any protein required for spindle assembly and chromosome alignment is in principle a negative regulator of the SAC because if you inhibit such proteins, you prevent spindle assembly and chromosome alignment and thus prevent SAC silencing. My advice would be to simply eliminate this focus on the SAC and simply build the narrative around their previous discoveries, i.e. in brief ... "UBASH3B is required to target Aurora B to the spindle, so to study this interaction and to determine whether disrupting it may open up therapeutic opportunities, we set out to better understand the interaction and develop small molecule disruptors".

2. Figure 1 presents a nice complementation assay revealing that the "2HP" mutant does not rescue the siUBASH3B phenotype. General point - the text annotating the figure is often very small making it hard to read without going back to the PDF and zooming in on the images. Why not eliminate extraneous text so that the key differentiators, e.g. WT vs 2HP, can be maximised? Including the schematic from S1a in the main figure would help the reader orient themselves with UBASH3B. The naming of the mutants is odd: FLAG UBASH3B WT vs FLAG UBASH3B - the FLAG tag is common to all so why not eliminate and simply refer to WT vs ΔPGM vs H391A vs 2HP, for example? In panels b-f, why not show the individual data points so the reader can get a better feel for the spread? The effect on mitotic timing observed by timelapse is modest, ~10 mins; would you expect this to manifest in a population-based biochemical assay (2g). There is no loading control in panel (g) and the Bub1 levels suggests that there is a lot more 2HP loaded. If so, is the difference in Cyclin B1 and pH3 simply because of the loading difference?
3. In Figure 2, the authors describe a molecule that binds UBASH3B and as a potential inhibitor they hereafter call it UBASHIN (line 165/6). This is very exciting. And yet, there are a number of UBASHINs, denoted by different superscripts. Various UBASHINs are used throughout and there are instances where the molecule used in the text is different from the one used in the figures. This must be resolved. Line 167 - to say that FD-E09 "selectively" induced cell death in HeLa cell, and that there is "selective" cytotoxicity is inappropriate at this stage in the narrative as there is no evidence that the phenotype is selective or what the selectivity is referring to. Does UBASH3B 2HP mutant disrupt Aurora B binding? Is this phenocopied by UBASHIN? What do the amino acids substitutions in the 2HP mutant do to the protein, or what are they expected to do? Did I miss these issues in the narrative?
4. Figure 3, S3 and Table S3 explore the binding of several derivatives to various mutants. This really needs clarifying into a format that allows side-by-side comparison of the entire dataset, rather than it spread across three display items. Note also that Table S3 is not a table, it is another figure and the numbers that one needs to be able to read in the top left corners need to be in a much bigger font size. Clarifying all this is critical; for example, to my eye, LIT-1238 binds UBASH3A similarly to UBASH3B, yet the authors state that LIT-1238 is selective for B over A like the other compounds (lines 215-217). Note that there is no discussion or schematic for the reader to see the relationship between A and B. Moreover, they then use LIT-1238 in cell-based assays (next section) even though the specificity for B over A is now in question. The key to the graphs is confusing; 2.5, 30 and 100 µM are repeated in different colours.
5. The story flip-flops around a bit. Top part of page 7 uses various UBASHINs but the synthesis of these additional derivatives is described after at the bottom of page 7 and 8.
6. The effect on Aurora B localisation is central to the story and would benefit from a more comprehensive high-res microscopy and quantitation. The images in 4i are far better than many of the Aurora B images (different microscopy platform?), and the quantitation of BubR1 pixel intensity is a good addition. There is a graph for Aurora B in 4h but this aspect is arguably more important. A key experiment therefore would be to expand this aspect with high quality images and pixel quantitation.
7. Page 9, in the text describing figure 5, the authors refer to LIT-1238 but the figure panels are labelled with LIT-13-108.
8. A key experiment here is a direct comparison with Taxol and Nocodazole. The effect of the UBASHINs can be explained by inhibition of chromosome alignment. It would be very important for the reader to know that this is not simply due to interfering with microtubule dynamics. Therefore, a side-by-side analysis of UBASHIN with some simple immunofluorescence analysis to look at localisation of Aurora B and UBASH3B, and spindle morphology, would be a very helpful.
9. Line 281, is it appropriate to refer to a "mitotic arrest", rather than a delay in chromosome alignment?
10. In Figure 6, the authors compare various cell lines, with the sensitive lines in the main figure and the resistant ones in supplementary; it would be preferable to see them side-by-side. This is not just about presentation, the colony formation assays in the main figure look odd, with large sections of the plates missing colonies, suggesting peeling rather than reduced proliferation. Panel (a) indicates cell death so a key experiment would be an independent assay, e.g. DNA content FACS with an apoptosis marker comparing sensitive with resistant. Page 11 and 6d - the attempts to explain the differential sensitivity of cell lines based on SAC status is not compelling and does not hold well for HCT116 vs RKO and DLD-1, all near-diploid, CIN-negative colon cancer cell lines with robust SAC responses. This is easily resolved by de-focusing on the SAC.
11. In relation to the "specificity" issue, are the resistant cell lines also resistant to siUBASH3B? In the Introduction, UBASH3 is described as non-essential in mice, can you generate a UBASH3B null cell line by CRISPR and show that UBASHIN has no additional effects in the absence of UBASH3B?
12. The xenograft data is a nice addition to show that the compounds have in vivo activity. The delocalisation of Aurora B seen in vitro is nicely confirmed.
13. Opening comments in Discussion re negative regulators of the SAC is not needed - see above.

We thank the reviewers for their constructive and insightful comments, which have greatly helped us improve the clarity, precision and impact of our manuscript. In summary, all three reviewers appreciated the high quality of the work and the significance of the study. Their suggestions regarding technical issues, additional experiments, and modifications to the narrative and presentation have been fully addressed in the revised version. Below, we outline the actions taken to address each point individually.

Referee #1 (Comments on Novelty/Model System for Author):

A GEMM or carcinogenesis mouse model would be much better but may be beyond the scope of this manuscript.

Referee #1 (Remarks for Author):

Cancer cell face unprecedented stress in mitosis due to defects in chromosome segregation and therefore have evolved to survive these challenges. One of the potential approaches is to push cancer cells over the brink by increasing mitotic stress, which then could lead to cell death but would have little effect on normal cells. The authors have taken advantage of this approach and have developed a chemical inhibitor of UBASH3B, which controls Aurora B localization and therefore the spindle assembly checkpoint. This inhibitor, UBASHIN, phenocopies UBASH3B deletion, leads to cancer cell death while not affecting normal (non-cancerous) cells. This inhibitor also affects cancer growth in vivo (mice).
This is a high-quality study presenting interesting results potentially paving the way for a less toxic form to replace the standard cancer therapy Paclitaxel. This manuscript is well-written and the experiments are done with high standards including all necessary controls.
We thank the reviewer for this very positive assessment and recognition of the study's quality, rigor, and potential impact for cancer therapy.

There are minor issues that need to be addressed:
1. In Figures 4,5,7,8, the authors do not label the first row with "merge", which is most common, but list all the antibodies. This is confusing.
We have revised throughout the manuscript the labelling of all composite microscopy panels. Specifically, we replaced the detailed antibody listings with "merge" to facilitate interpretation as suggested by the reviewer.

2. Line 142: "GFP-UBASH3B WT was enriched at the spindle, as" this description does not completely match what the reviewer sees in Figure 1h and this should be improved.
We thank the reviewer for this comment and have revised the figure with more representative images of GFP-UBASH3B localization. In the updated **Fig. 2H**, we now include examples of both metaphase and anaphase cells, showing that GFP-UBASH3B wild-type form localizes in part to the mitotic spindle and centrosomes during metaphase and to the midzone during anaphase. We also improved the representative images of endogenous UBASH3B localization, which show a similar spindle-associated pattern **(Fig. 6B)**, and are further validated by siRNA-

mediated depletion **(Fig. 6A)**. Together, these revisions better support the statement that UBASH3B is enriched at mitotic spindle structures.

3. Line 161, 196: "FB detection, FPA experiments" these abbreviations have not been introduced.
We have corrected the text so that FP is introduced as fluorescence polarization at first use, and we have also fixed the typographical error in "FPA experiments" by replacing it with "FP experiments".

4. Line 175: "derivatives UBASHINLIT-1238 and UBASHINLIT-1236, along" the names of the derivatives is confusing and could be improved.
We agree that the original nomenclature of the derivatives was confusing, a point also raised by reviewer 3 in his/her comment 3. To improve clarity and avoid any ambiguity, we have renamed the compounds simply as UBASHIN-1 to UBASHIN-5 throughout the revised manuscript. The correspondence between these names and the original derivatives is clearly indicated in **Appendix Fig. S1A**.

5. Line 239: "prevents SAC satisfaction" please rephrase.
We have rephrased this and related language throughout the manuscript. Following the extensive revisions made in response to Reviewer 3 (comment 1), we replaced broad SAC-centric terminology with more precise descriptions of the underlying biology. Specifically, terms such as "SAC satisfaction," "SAC strength," "SAC potentiation," and "negative SAC regulator" have been removed and replaced with more accurate phrasing including "checkpoint-associated mitotic signaling," "sustained checkpoint engagement," "delayed mitotic exit," and "mitotic arrest response."

6. Line 289,290: "displayed minimal sensitivity to the inhibitor, with" As the authors know, the sensitivity of cells to drugs is dose dependent. Have the authors used a wide range of concentrations of UBASHIN? It would be good to know up to which concentration these cells are not sensitive to UBASHIN.
The point on drug sensitivity is important. To address it, we performed a dose-response analysis in selected responsive and non-responsive cell lines and assessed cell viability by trypan blue exclusion. These analyses showed that the responsive cell lines reached maximal effect in the low micromolar range, whereas the non-responsive lines remained largely unaffected across the tested concentrations, with only a minor effect at the highest dose, which coincided with clear solubility limitations. Because poor solubility can alter the effective concentration in culture and increase assay variability, we consider this isolated effect unlikely to represent a specific biological response. These new results are included in **Fig. 7D** and are described in the Results section, lines 321-331.

7. Figure 7 and 8 could be fused since in both cases they are xenograft models. A GEMM or carcinogenesis mouse model would be much better but may be beyond the scope of this manuscript.

We appreciate the reviewer's suggestion. We considered merging Figures 7 and 8 **(revised Figures 9 and 10)**, since both *in vivo* studies include similar histological analyses (Ki67, TUNEL, pH3S10 and Aurora B). However, we decided to keep them separate because the two experiments differ in their biological model and primary readout: the breast cancer study **(Figure 9)** uses an MDA-MB-231-Luc2 xenograft model in Balb/c nude mice, monitored by longitudinal bioluminescence imaging and caliper measurements, whereas the colorectal cancer study **(Figure 10)** uses a CT26 syngeneic model in Balb/c mice, monitored by caliper measurements alone. In addition, we moved the detailed experimental timelines (**Figures 9A and 10A)** and the H&E necrosis staining (**Figures 9D and 10D)** from the Supplementary Information into the main figures, so that each xenograft model is now presented with its own complete set of *in vivo* readouts. We believe that this organization better preserves readability, while allowing each model to be interpreted in its specific biological and experimental context. We also agree that while these *in vivo* studies provide proof-of-principle for UBASHIN efficacy *in vivo*, future work in genetically engineered or carcinogenesis-driven mouse models will be important to assess its activity in more physiologically relevant tumor settings. (Discussion section, lines 666-672).

Referee #2 (Comments on Novelty/Model System for Author):

The work presents here is novel, has high medical impact and good technical quality

Referee #2 (Remarks for Author):

The manuscript by Pangou et al. addresses an important topic in cancer research, aiming to develop new therapeutic strategies to target aggressive cancers characterized by aneuploidy and chromosome instability.
The authors focus on UBASH3B protein, a regulator of mitotic kinase Aurora B. Building on their previous work on the UBASH3B protein, the authors identify a druggable region of the protein (2HP) and apply a comprehensive set of approaches - including molecular cloning, biochemistry, chemistry, cell biology, live cell imaging and in vivo studies - to identify a new compound termed UBASHIN, that specifically inhibits UBASH3B.
The manuscript reports a complete translational pipeline, progressing from compound discovery and mechanistic characterization to therapeutic evaluation in vitro and in pre-clinical models.
The manuscript is well-written and easy to read, the experiments are carefully performed and the results are novel, compelling and convincing.
We thank the reviewer for this thorough and highly positive evaluation of our study's scope, technical approach, and impact. We greatly appreciate these encouraging remarks.

However, several points can be raised, particularly concerning the relevance of these findings for cancer patients:

* Authors should investigate whether high vs low UBASH3B levels are associated with CIN and/or aneuploidy in cancer patients, using publicly available transcriptomic databases.

We appreciate the reviewer's valuable suggestion. We have analyzed public TCGA datasets corresponding to the cancer types represented in our cell line panel. UBASH3B expression showed only weak and inconsistent correlations with aneuploidy across these tumor types. Moreover, these trends were in line with our chromosome-spread data described in the comment below, which showed only modest effects of UBASH3B manipulation on ploidy **(Fig. 8G,H)**. Together, these results argue against a strong or universal association between UBASH3B and CIN, and most likely support a tumor-type-dependent relationship. These new results are included in **Appendix Fig. S2B** and are described in the Results section, lines 427-435.

* Authors should perform chromosome spread analysis to measure ploidy status of cancer cells following depletion of UBASH3B and upon overexpression of full-length UBASH3B and its mutants.

We appreciate the reviewer's valid suggestion. We performed chromosome spread analysis after UBASH3B depletion and after overexpression of UBASH3B wild-type or 2HP mutant in two UBASHIN-responsive cell lines with distinct karyotypic backgrounds: the highly aneuploid MDA-MB-231 cells and the nearly diploid HCT116 cells. These experiments showed only modest effects on ploidy, consistent with our TCGA analysis **(Appendix Fig. S2B)** described in the comment above. Together, these data suggest that UBASH3B does not broadly affect CIN and that CIN is unlikely to be the main determinant of UBASHIN sensitivity. These new results are included in **Fig. 8G,H** and are described in the Results section, lines 418-426.

*How do authors explain that HCT116 cells are responsive to UBASHIN, given that these cells are nearly diploid and genetically stable?

We appreciate the reviewer's insightful comment. As outlined in our responses above, our chromosome-spread analysis and TCGA-based analyses indicate that UBASHIN sensitivity is not simply determined by basal aneuploidy or CIN. HCT116 cells are nearly diploid and comparatively stable karyotypically, but they remain transformed colorectal cancer cells with cancer-associated alterations in mitotic regulation. Their responsiveness to UBASHIN therefore supports the idea that sensitivity reflects a cell-context-specific dependence on UBASH3B-mediated regulation of Aurora B and mitotic progression, rather than a requirement for pre-existing high CIN.

* The question can be raised whether UBASHIN treatment may benefit to a larger panel of cancer patients. More particularly, is UBASHIN active on cancer cells in which aneuploid/CIN has been induced independently of UBASH3B, by means of centrosome amplification (Plk4), MPS1 inhibition (reversin) or other ways.

We acknowledge the importance of testing UBASHIN in cells with CIN induced by alternative mechanisms. To address this, we tested the effect of UBASHIN in combination with two clinically relevant MPS1 inhibitors: Empesertib (Sauro et al., Mol Cancer Ther 2016) and

BAY1217389 (Maia et al., Br J Cancer 2018) in MDA-MB-231 cells, which are responsive to UBASHIN monotherapy **(Fig. 7)** and validated for MPS1 inhibitor studies (Maia et al., 2018). Cell viability was assessed by Trypan Blue exclusion after short-term treatment (16h) with the respective inhibitors. Under these conditions, UBASHIN alone induced the expected cell death, whereas MPS1 inhibition alone did not produce a significant Trypan Blue-positive death signal, consistent with reports that MPS1 inhibitors often show stronger cytotoxic activity in combination with taxanes under conditions of pronounced mitotic stress. Interestingly, concomitant MPS1 inhibition did not enhance UBASHIN-induced cytotoxicity but rather attenuated it. These new results are included in **Fig. 8E** and are described in the Results section, lines 395-405.

Since MPS1 inhibition weakens mitotic checkpoint signaling and can promote premature mitotic exit or slippage, our short-term Trypan Blue assay may not capture delayed post-mitotic loss of viability. Thus, we cannot exclude that different assay formats, longer treatments, clonogenic assays, or sequential drug addition could reveal additional effects. Nevertheless, within the limits of this experiment, the lack of synergy with MPS1 inhibition suggests that UBASHIN is not simply acting as a generic CIN enhancer as outlined above. Rather, the data are more consistent with UBASHIN inducing a specific UBASH3B-dependent mitotic perturbation, linked to altered Aurora B localization, whose cytotoxic outcome may require a checkpoint-competent mitotic state.

*Is UBASHIN also active on cancer cells overexpressing Aurora B, considering that elevated expression of this mitotic kinase is associated with cancer aggressiveness ?

We appreciate the reviewer's important comment regarding Aurora B overexpression. Since UBASHIN affects Aurora B localization rather than total Aurora B abundance, we asked whether increasing Aurora B levels would be sufficient to overcome the effect of UBASH3B inhibition. Our new data indicate that UBASHIN remained active in cells overexpressing Aurora B. This suggests that the relevant vulnerability is not simply the amount of Aurora B present in the cell, but its UBASH3B-dependent spatial regulation during mitosis. Thus, elevated Aurora B expression, although associated with cancer aggressiveness, is unlikely on its own to bypass UBASHIN response. These new results are included in **EV4D,E** and are described in the Results section, lines 349-358.

*Would patients benefit from combining UBASHIN and paclitaxel? Authors should analyze the effects of combining both compounds on cancer cells that respond, and on those that do not respond - to UBASHIN alone.

We appreciate the reviewer's highly relevant suggestion. We tested the combination of UBASHIN and Paclitaxel in cancer cell lines that either respond or do not respond to UBASHIN monotherapy. These experiments showed that the benefit of the combination is context-dependent. In cells already sensitive to UBASHIN, adding Paclitaxel did not further increase cell death, suggesting that UBASH3B inhibition may already impose a strong mitotic burden under these conditions. In contrast, in cells that were poorly responsive to UBASHIN alone, Paclitaxel sensitized cells to UBASHIN treatment. This suggests that Paclitaxel-induced mitotic stress can expose or enhance dependence on UBASH3B-linked mitotic regulation in

specific cellular contexts. These new results are included in **Fig. 8F** and are described in the Results section, lines 406-417.

Minor points
* What is the mechanism that the authors do favor to explain UBASHIN-induced mislocalization of UBASH3B from the mitotic spindle?

We thank the reviewer for this important question. Based on our data, we favor a model in which UBASHIN disrupts a 2HP-dependent spindle-targeting or spindle-retention function of UBASH3B, rather than acting as a general microtubule poison. This interpretation is supported by the observation that mutation of the 2HP domain causes UBASH3B to become diffuse and reduces its enrichment at spindle-associated structures **(Fig. 2H,I**) and that UBASHIN treatment similarly mislocalizes endogenous UBASH3B from the mitotic spindle **(Fig. 6B,C)**. Importantly, UBASHIN does not detectably disrupt the interaction of UBASH3B with Aurora B or Mklp2, suggesting that the compound does not simply dissociate the known UBASH3B–Aurora B/Mklp2 complex **(EV3D,E)**. We therefore consider it likely that UBASHIN interferes either with a 2HP-dependent interaction between UBASH3B and mitotic microtubules/spindle-associated factors, or with a 2HP-dependent conformation required for UBASH3B spindle retention.

In the revised manuscript, we also discuss the possibility that the 2HP domain contributes to microtubule or spindle organization through an enzymatic or pseudoenzymatic mechanism. However, because the relevant substrate or spindle adaptor has not yet been identified, we present this as a working hypothesis rather than a definitive mechanism. Consistently, our comparison with nocodazole and Paclitaxel indicates that Aurora B mislocalization after UBASHIN treatment is not simply a secondary consequence of general microtubule disruption **(EV3F,G)**. We have revised the Discussion to clarify these possible mechanisms and the limitations of the current model (lines 582-594).

*Fig. 4j is mislabeled and appears as panel g in the Figure

We thank the reviewer for pointing this out. The panel previously mislabeled as Fig. 4G is now correctly labeled as **Fig. 5I** in the revised manuscript.

Referee #3 (Remarks for Author):

Pangour/Sumara et al Embo Molecular Medicine

The team previously discovered that UBASH3B binds mono-ubiquitinated Aurora B and that this promotes binding of Aurora B to the mitotic spindle. Here, they identify small molecules that bind UBASH3B via the 2HP domain. These drugs delocalise Aurora B from the spindle, disrupt mitotic chromosome alignment, are toxic in a subset of cancer cell lines and can suppress tumour growth in vivo. Overall the data is convincing but there are a few specific experiments that would help support their conclusions. My main criticism however is that the story is very difficult to follow - the reader has to work very hard to track the different mutants, drugs and read the figures. These issues can be addressed with changes to the presentation. If

the authors were to tighten up the narrative and presentation the story would be much more accessible.

We thank the reviewer for their appreciation of the quality of our work, as well as for their helpful overall assessment and practical suggestions. Although Reviewers 1 and 2 found the narrative and data presentation clear, we have made additional efforts to further improve the structure and figures in order to make the manuscript more accessible and easier to follow.

1. Throughout there is a lot of focus on the SAC. However, the thinking is often confused and/or confusing, so much so that it becomes a distraction. For example, the second sentence of the Introduction states that the SAC "oversees" various aspect of mitosis. This is not a correct description of the checkpoint - the SAC is constitutively active in mitosis until all KTs are stably attached to MTs. Yes, this is predicated on spindle assembly and chromosome alignment, but the SAC does not "oversee" these processes. Much of the next paragraph is also very vague - the SAC being "weaker" in many cancer cells (line 59) is a vague statement, citing on a 20-year-old review. The use of the word mitotic "catastrophe" is not helpful in this context. What are the regulators vs core components of the SAC? Later in the manuscript the authors set out to measure SAC "strength" (line 299). Again, this is a rather meaningless concept in the context of the experiment performed. The idea that UBASH3B is a negative regulator of the SAC is also not informative (line 74) in that any protein required for spindle assembly and chromosome alignment is in principle a negative regulator of the SAC because if you inhibit such proteins, you prevent spindle assembly and chromosome alignment and thus prevent SAC silencing. My advice would be to simply eliminate this focus on the SAC and simply build the narrative around their previous discoveries, i.e. in brief ... "UBASH3B is required to target Aurora B to the spindle, so to study this interaction and to determine whether disrupting it may open up therapeutic opportunities, we set out to better understand the interaction and develop small molecule disruptors".

We thank the reviewer for this important clarification. We agree that the previous version placed too much emphasis on the SAC and could be confusing. We have therefore substantially revised the manuscript to refocus the narrative on UBASH3B-dependent Aurora B regulation and mitotic progression. In line with this, we changed the title to remove SAC-centered wording, corrected the SAC definition in the Introduction, removed vague statements such as a “weaker SAC,” replaced the older citation with a more recent review, and removed terms such as “negative SAC regulator,” “SAC satisfaction,” “SAC strength,” “SAC potentiation,” and “mitotic catastrophe.” We now use more precise and cautious wording, including “checkpoint-associated mitotic signaling,” “sustained checkpoint engagement,” “mitotic arrest response,” “delayed mitotic exit,” and “mitotic cell death.” The experiment previously referred to as measuring “SAC strength” has also been renamed as a “Paclitaxel-induced prometaphase arrest assay,” which more accurately describes the readout. Finally, we now interpret BubR1, Cyclin B1/pH3S10, MPS1 inhibitor and Paclitaxel data as checkpoint-associated consequences of altered mitotic progression, rather than evidence that UBASH3B is a core SAC component. We believe these revisions address the reviewer’s concern and make the narrative clearer: UBASH3B is required for proper Aurora B spatial regulation during mitosis, and

pharmacological disruption of this pathway by UBASHIN exposes a mitotic vulnerability in selected cancer cells.

2. Figure 1 presents a nice complementation assay revealing that the "2HP" mutant does not rescue the siUBASH3B phenotype. General point - the text annotating the figure is often very small making it hard to read without going back to the PDF and zooming in on the images. Why not eliminate extraneous text so that the key differentiators, e.g. WT vs 2HP, can be maximised? Including the schematic from S1a in the main figure would help the reader orient themselves with UBASH3B. The naming of the mutants is odd: FLAG UBASH3B WT vs FLAG UBASH3B - the FLAG tag is common to all so why not eliminate and simply refer to WT vs ΔPGM vs H391A vs 2HP, for example? In panels b-f, why not show the individual data points so the reader can get a better feel for the spread? The effect on mitotic timing observed by timelapse is modest, ~10 mins; would you expect this to manifest in a population-based biochemical assay (2g). There is no loading control in panel (g) and the Bub1 levels suggests that there is a lot more 2HP loaded. If so, is the difference in Cyclin B1 and pH3 simply because of the loading difference?

We thank the reviewer for these constructive and detailed comments. We have enlarged the text in the figures, removed unnecessary annotations where possible and simplified the mutant nomenclature. An exception was made for the FLAG tag, as the rescue experiment includes an important FLAG-tag-only control essential for its completeness. The entire previous Supplementary Figure S1 including the UBASH3B domain schematic has been moved into the Main Figure 1 to make the data presentation easier to follow. We also followed the reviewer's suggestion to improve data visualization by showing the individual data points in the relevant quantifications throughout the Figures. Regarding the loading control, GAPDH was already included in Figure 2G and shows comparable loading between the WT and 2HP conditions. Finally, concerning the mitotic timing measurement as quantified by live-cell imaging, we respectfully note that a ~10-minute delay in mitosis is biologically meaningful in mammalian cells. Mitotic duration is normally tightly constrained and even relatively small extensions can be integrated by mitotic surveillance pathways to influence p53/p21 signaling and daughter-cell fate. We have clarified this point in the text (Results section lines 132-135 and Discussion section lines 536-545) and referenced relevant literature to better contextualize the significance of this observation (Meitinger *et al*, 2024; Sparr & Meitinger, 2025; Fulcher *et al*, 2025).

3. In Figure 2, the authors describe a molecule that binds UBASH3B and as a potential inhibitor they hereafter call it UBASHIN (line 165/6). This is very exciting. And yet, there are a number of UBASHINs, denoted by different superscripts. Various UBASHINs are used throughout and there are instances where the molecule used in the text is different from the one used in the figures. This must be resolved. Line 167 - to say that FD-E09 "selectively" induced cell death in HeLa cell, and that there is "selective" cytotoxicity is inappropriate at this stage in the narrative as there is no evidence that the phenotype is selective or what the selectivity is referring to. Does UBASH3B 2HP mutant disrupt Aurora B binding? Is this phenocopied by UBASHIN? What do the amino acids substitutions in the 2HP mutant do to the protein, or what are they expected to do? Did I miss these issues in the narrative?

We agree that the original nomenclature of the derivatives was confusing, a point also raised by reviewer 1 in his/her comment 4. To improve clarity and avoid any ambiguity, we have renamed the compounds simply as UBASHIN-1 to UBASHIN-5 throughout the revised manuscript. The correspondence between these names and the original derivatives is clearly indicated in Appendix Fig. S1. We also carefully checked the text and figures to ensure that the compound name used in the text matches the corresponding figure panel. For readability, we generally refer to the active compound series as UBASHIN in the text and use the numbering only when individual compounds are compared directly to each other.

We also agree that the use of the word "selective" was premature at that stage of the original results. We have therefore replaced it with more cautious phrasing, such as "differential response" and "cancer-associated vulnerabilities" (Results section lines 171-172). Importantly, our new results now better support selectivity, based on the dose-response analyses added in response to Reviewer 1, Comment 6, and the specificity experiments showing that UBASHIN has no additional effect when UBASH3B is depleted, as described below in our response to Reviewer 3, Comment 11. These new data are included in **Fig. 7D,E** respectively.

Regarding the 2HP mutant, we had already described in original manuscript (Results, Discussion and Methods sections), that this construct carries alanine substitutions in the two conserved catalytic motifs of the 2HP domain: H127A/T129A and H212A/T214A. However, the reviewer is right in that we did not adequately explain the rationale for designing the 2HP mutant when the mutants were introduced. These residues were selected because the conserved histidines are predicted catalytic residues of the 2H phosphoesterase active site, while the adjacent threonines belong to the same conserved motifs and are expected to contribute to active-site organization and/or substrate positioning (Results section lines 108-111). Thus, these substitutions are expected to impair the phosphodiesterase-related function of the 2HP domain and may also affect local structural features important for UBASH3B mitotic function.

Regarding the Aurora B binding, our original co-IP experiments showed that UBASHIN does not detectably disrupt the interaction of UBASH3B with Aurora B or Mklp2 **(EV3D)**. To further address this point, we have now added an *in vitro* binding experiment using purified GST-tagged 2HP-MUT UBASH3B, which shows that the 2HP-mutant protein still binds Aurora B and Mklp2, and further confirms that UBASHIN does not disrupt these interactions **(EV3E).** These data are described in Results section lines 263-271**.**

4. Figure 3, S3 and Table S3 explore the binding of several derivatives to various mutants. This really needs clarifying into a format that allows side-by-side comparison of the entire dataset, rather than it spread across three display items. Note also that Table S3 is not a table, it is another figure and the numbers that one needs to be able to read in the top left corners need to be in a much bigger font size. Clarifying all this is critical; for example, to my eye, LIT-1238 binds UBASH3A similarly to UBASH3B, yet the authors state that LIT-1238 is selective for B over A like the other compounds (lines 215-217). Note that there is no discussion or schematic for the reader to see the relationship between A and B. Moreover, they then use LIT-1238 in cell-based assays (next section) even though the specificity for B over A is now in question. The key to the graphs is confusing; 2.5, 30 and 100 µM are repeated in different colours.

We thank the reviewer for this important comment. To address the concern, we have substantially restructured the presentation of the SPR data to allow direct side-by-side comparison of the full dataset **(Fig. 4A)**. Each column corresponds to a purified protein and each row corresponds to a compound. We also enlarged axis and legend fonts, and we corrected the mistake regarding the colour code and the actual concentrations used. Due to Figure size restrictions, the steady-state binding curves are presented in **EV2**, however we have included the number of the KD values on the corresponding sensorgrams **(Fig. 4A)**, to allow for direct comparisons.

Regarding the binding specificity of the UBASHIN derivatives to the various proteins tested, we have clarified that the derived KD values should be interpreted as apparent affinity estimates, since the SPR responses did not always reach a clear saturation plateau **(**Results section, lines 217-220 and EV2 Legend, lines 1520-1522). The original presentation could indeed give a misleading impression, because detectable sensorgram responses were observed even when the equilibrium binding curves did not fully saturate, making the KD values approximate rather than exact equilibrium constants. To address this, we generated a new summary panel **(Fig. 4B)** that integrates the entire SPR dataset side-by-side and explicitly separates robust binding preference from weaker or compound-dependent interactions. This revised presentation makes clear that the UBASHIN series preferentially engages the UBASH3B 2HP domain, whereas UBASH3A shows only weak and compound-dependent interaction, and it also helps distinguish the biochemical SPR data from the cellular phenotypes measured in the functional assays. Importantly, this finding does not change the main biological interpretation of our study, because the two paralogs are functionally distinct: UBASH3A is best known for immune signaling roles in T cells (Wang *et al*, 2025) whereas UBASH3B is the paralog linked to Aurora B recruitment to mitotic microtubules and control of chromosome segregation (Krupina 2016, 2018). Thus, although UBASHINS show partial cross-reactivity to UBASH3A *in vitro*, the cell-based mitotic phenotypes in our models remain most consistent with modulation of the UBASH3B-dependent pathway. To help the reader, we also added a brief explanation of the relationship between UBASH3A and UBASH3B in the Discussion section**,** lines 562-574, so that the paralog comparison is explicit rather than implied.

5. The story flip-flops around a bit. Top part of page 7 uses various UBASHINs but the synthesis of these additional derivatives is described after at the bottom of page 7 and 8.

We thank the reviewer for this helpful observation. We have revised the text so that the synthesis of additional UBASHIN derivatives is now described before their use in experimental set ups (Results section, lines 164-167, 173-179 and 201-203).

6. The effect on Aurora B localisation is central to the story and would benefit from a more comprehensive high-res microscopy and quantitation. The images in 4i are far better than many of the Aurora B images (different microscopy platform?), and the quantitation of BubR1 pixel intensity is a good addition. There is a graph for Aurora B in 4h but this aspect is arguably more important. A key experiment therefore would be to expand this aspect with high quality images and pixel quantitation.

We thank the reviewer for this suggestion. We have replaced several representative Aurora B images with higher-resolution examples and added zoomed regions of interest to better illustrate the chromosome arm spreading phenotype. These improved images are now shown in **Fig. 5F, EV3A, and EV3F.**

Regarding quantification, the rationale differs between Aurora B and BubR1. BubR1 is expected to localize at kinetochores, and therefore measuring BubR1 intensity relative to CREST provides a useful readout of kinetochore-associated checkpoint-marker accumulation. In contrast, the Aurora B phenotype in our study is primarily spatial: Aurora B is either concentrated at centromeres or redistributed along chromosome arms. For this reason, we quantified the percentage of cells displaying Aurora B chromosome arm spreading rather than pixel intensity relative to a kinetochore marker, as this better captures the phenotype relevant to UBASH3B inhibition.

7. Page 9, in the text describing figure 5, the authors refer to LIT-1238 but the figure panels are labelled with LIT-13-108.

We thank the reviewer for noticing this mistake. We have carefully reviewed and corrected all figure labels and corresponding text to ensure that compound identities are consistent throughout the manuscript. Please also refer to our responses to reviewer 1/comment 4 and reviewer 3/comment 3 regarding the nomenclature of the UBASHIN derivatives.

8. A key experiment here is a direct comparison with Taxol and Nocodazole. The effect of the UBASHINs can be explained by inhibition of chromosome alignment. It would be very important for the reader to know that this is not simply due to interfering with microtubule dynamics. Therefore, a side-by-side analysis of UBASHIN with some simple immunofluorescence analysis to look at localisation of Aurora B and UBASH3B, and spindle morphology, would be a very helpful.

We thank the reviewer for this suggestion. We have now tested the localization of Aurora B after Nocodazole and Paclitaxel treatment. As expected from the literature (Kelly *et al*, 2007; Wang *et al*, 2012), Nocodazole and Paclitaxel altered spindle organization but did not reproduce the Aurora B chromosome-arm spreading observed with UBASHIN. Thus, the effect of UBASHIN on Aurora B localization is not explained simply by inhibition of chromosome alignment or general disruption of microtubule dynamics. These new data are included in **EV3F,G** and described in the Results section, lines 294-302.

9. Line 281, is it appropriate to refer to a "mitotic arrest", rather than a delay in chromosome alignment?

We have revised the text according to the reviewer's suggestion (line 305).

10. In Figure 6, the authors compare various cell lines, with the sensitive lines in the main figure and the resistant ones in supplementary; it would be preferable to see them side-by-side. This is not just about presentation, the colony formation assays in the main figure look odd, with large sections of the plates missing colonies, suggesting peeling rather than reduced proliferation. Panel (a) indicates cell death so a key experiment would be an independent assay,

e.g. DNA content FACS with an apoptosis marker comparing sensitive with resistant. Page 11 and 6d - the attempts to explain the differential sensitivity of cell lines based on SAC status is not compelling and does not hold well for HCT116 vs RKO and DLD-1, all near-diploid, CIN-negative colon cancer cell lines with robust SAC responses. This is easily resolved by de-focusing on the SAC.

We thank the reviewer for this helpful comment. Due to figure size limitations, we kept separate the responsive and non-responsive cell lines in **Fig. 7B,C and EV4B,C** respectively. However, we have strengthened the analysis of differential sensitivity with new experiments presented in **Fig. 7D,E.** Please refer to our responses to reviewer 1/comment 6 and reviewer 3/comment 11. We agree that distinguishing between reduced proliferation and apoptosis is important for understanding the compound's effects and we fully acknowledge that both apoptosis and reduced proliferation may contribute to the loss of colonies following inhibitor exposure. Therefore, we have clarified in the revised text that we cannot exclude the possibility that UBASHIN has both short-term and long-term effects on cell death and proliferation in these assays (lines 316-318). Moreover, our conclusions are supported by the quantitative reduction in colony number, which was consistent across biological replicates, rather than by the visual continuity of individual plate regions. The apparent empty areas likely reflect plate handling/imaging constraints in the representative images and do not affect the biological interpretation.

Regarding the suggestion for an independent cell-based apoptosis assay, we would like to point out that our study provides multiple lines of evidence for apoptosis induction upon treatment with our inhibitor. Specifically, in our two *in vivo* models, we performed TUNEL assays that directly demonstrate apoptosis within tumors following UBASHIN treatment **(Fig. 9E,G and 10E,G)**. These results validate apoptosis induction by the inhibitor in multiple settings relevant to disease context. Moreover, in **Fig. 8D**, we present immunoblot evidence of cleaved PARP1 upon inhibitor treatment in the responsive MDA-MB-231 cells but not in the non-responsive MCF7 cells, which is a widely accepted marker of apoptosis in mammalian cells. This, together with our *in vivo* TUNEL results, provides strong support for apoptosis as a biologically relevant outcome both *in vitro* and *in vivo*. Therefore, and in agreement with the editor, repeating additional cellular apoptosis assays in the same *in vitro* models would be redundant and would not substantially enhance the mechanistic conclusions of the study

Finally, we have revised the text to de-emphasize SAC status as an explanation for the observed differences in cell line sensitivity. Please refer to our response to reviewer 3/comment 1.

11. In relation to the "specificity" issue, are the resistant cell lines also resistant to siUBASH3B? In the Introduction, UBASH3 is described as non-essential in mice, can you generate a UBASH3B null cell line by CRISPR and show that UBASHIN has no additional effects in the absence of UBASH3B?

We thank the reviewer for raising this important specificity question. To address this point, we silenced UBASH3B by siRNA in selected non-responsive cell lines and assessed cell viability by trypan blue exclusion. In addition, we performed combined UBASH3B knockdown and UBASHIN treatment in selected responsive cell lines to test whether UBASHIN retained any additional effect in the absence of UBASH3B. These experiments showed that non-responsive

cell lines remained largely unaffected by UBASH3B depletion, indicating that UBASH3B is dispensable for their proliferation. Moreover, concomitant genetic and pharmacologic perturbation of UBASH3B in responsive cell lines did not produce additive phenotypes, strongly supporting that UBASHIN exerts its cytotoxic activity through specific inhibition of UBASH3B rather than through UBASH3B-independent off-target effects. We also note that, in agreement with the editor, we did not pursue UBASH3B knockout cell lines, and siRNA-mediated depletion was considered sufficient to address the specificity question in this revised study. These new results are included in **Fig. 7E** and are described in the Results section, lines 332-341.

12. The xenograft data is a nice addition to show that the compounds have in vivo activity. The delocalisation of Aurora B seen in vitro is nicely confirmed.
We thank the reviewer for this positive feedback.

13. Opening comments in Discussion re negative regulators of the SAC is not needed - see above.
Please refer to our response to reviewer 3/comment 1.

27th Jul 2026

Dear Dr. Sumara,

Thank you for submitting your revised manuscript. The revision was evaluated by referees #1 and #2. As referee #3 was not available to assess the revised manuscript, referee #1 also reviewed your responses to referee #3's comments. As you will see from the reports below, the reviewers are satisfied with the revisions, and I will therefore be able to accept your manuscript pending the following minor revisions:

1/ Manuscript text:
- Please remove the red font text, and only keep in track changes any new modification.
- Please correct the headings and the order of the sections in the manuscript text to: Abstract / The Paper Explained / Introduction / Results / Discussion / Methods / Data Availability / Acknowledgements / Disclosure and Competing Interests Statement / References / Figure Legends / Expanded View Figure Legends.
- Please remove the "Ethics, Consent to Participate, and Consent to Publish declarations" section.
- Please change the heading from "Materials and Methods" to "Methods":
o Cells: indicate whether the cells were authenticated and tested for mycoplasma contamination.
o Statistics: please provide a statement on sample size, inclusion/exclusion criteria, blinding and randomization.
- Data Availability Section: Only newly generated datasets and codes/scripts must be listed here. Please ensure that custom scripts are deposited or included in the manuscript. The remaining text should be removed.
- Funding: merge with Acknowledgments; please add the grant numbers to the funders list in our system and ensure that the list is complete and accurate.
- Competing Interest should be renamed 'Disclosure and competing interests statement'.
- References: Our journal encourages inclusion of *data citations in the reference list* to directly cite datasets that were re-used and obtained from public databases. Data citations in the article text are distinct from normal bibliographical citations and should directly link to the database records from which the data can be accessed. In the main text, data citations are formatted as follows: "Data ref: Smith et al, 2001" or "Data ref: NCBI Sequence Read Archive PRJNA342805, 2017". In the Reference list, data citations must be labeled with "[DATASET]". A data reference must provide the database name, accession number/identifiers and a resolvable link to the landing page from which the data can be accessed at the end of the reference.

2/ Figures:
- Remove the figures from the manuscript text file.
- The two appendix figures can be made EV figures; otherwise, please compile them in one PDF, label the file "Appendix", add a table of contents with page numbers, and add the figure legends underneath the corresponding figures.
- In the expanded view figure legends, please correct the nomenclature to "Figure EV1" etc.
- The movie files need legends zipped to the corresp. movie files.
- During our standard figure check, similarities were found between figures 9E and 10E. Please carefully check the composition of your figures and clarify. Please note that figure reuse is allowed, but should be mentioned in the figure legend.
- Please address the queries from our data editors in the figures or their legends:
1. Please note that information related to n is missing in the legends of figures 3B; EV-5A.
2. For n=2, please remove statistics and error bars and only provide individual data points (i.e. Figure 3B).
3. Please note that the scale bar is missing for figures 10D; EV-3A,F.
4. Please note that scale bar and its definition are missing for figure 10B.
5. Please note that the black arrowheads are not defined in the legend of figure 10D; This needs to be rectified.

3/ Author checklist:
- Please check the section Cell materials/primary cultures, as I don't think it applies to your study.
- Please check that the 'experimental study design and statistics' section accurately reflects the information provided in the manuscript
- Please correct the 'Data availability' section (primary datasets and data citations) if needed.

4/ Visual abstract:
Please adjust the dimensions to 550 pixels wide x 300 pixels high, and make sure the text remains legible.

5/ As part of the EMBO Publications transparent editorial process initiative (see our Editorial at http://embomolmed.embopress.org/content/2/9/329), EMBO Molecular Medicine will publish online a Review Process File (RPF) to accompany accepted manuscripts.
This file will be published in conjunction with your paper and will include the anonymous referee reports, your point-by-point response and all pertinent correspondence relating to the manuscript. Let us know whether you agree with the publication of the RPF.
Please note that the Authors checklist will be published at the end of the RPF.

I look forward to receiving your revised manuscript.

Yours sincerely,

Lise Roth
Lise Roth, PhD
Senior Editor
EMBO Molecular Medicine

***** Reviewer's comments *****

Referee #1 (Remarks for Author):

The authors have done extensive revisions and have addressed all issues raised by the reviewers. The manuscript is improved and can be published now.

Referee #2 (Remarks for Author):

is suitable for publication

The authors addressed the remaining editorial issues.

26th Aug 2026

Dear Dr. Sumara,

Thank you for submitting your revised files. I am pleased to inform you that your manuscript is accepted for publication and is now being sent to our publisher to be included in the next available issue of EMBO Molecular Medicine.

Your manuscript will be processed for publication by EMBO Press. It will be copy edited and you will receive page proofs prior to publication. Please note that you will be contacted by Springer Nature Author Services to complete licensing and payment information.

You may qualify for financial assistance for your publication charges - either via a Springer Nature fully open access agreement or an EMBO initiative. Check your eligibility: https://link.springer.com/journal/44321/how-to-publish-with-us

Should you be planning a Press Release on your article, please notify the production team via this form in order to coordinate publication and release dates: https://forms.cloud.microsoft/e/eH4A8JVP6g

If you have any questions, please do not hesitate to contact the Editorial Office.
Thank you for your nice contribution to EMBO Molecular Medicine!

With kind regards,

Lise

Lise Roth, Ph.D
Senior Editor
EMBO Molecular Medicine

>>> Please note that it is EMBO Molecular Medicine policy for the transcript of the editorial process (containing referee reports and your response letter) to be published as an online supplement to each paper. If you do NOT want this, you will need to inform the Editorial Office via email immediately. More information is available here: https://link.springer.com/partners/embo-press/editorial-policies#Peer%20review

EMBO Press Author Checklist

Corresponding Author Name: Izabela Sumara and Evanthia Pangou	
Journal Submitted to: EMBO Molecular Medicine	
Manuscript Number: EMM-2025-22751	

USEFUL LINKS FOR COMPLETING THIS FORM
The EMBO Journal - Author Guidelines
EMBO Reports - Author Guidelines
Molecular Systems Biology - Author Guidelines
EMBO Molecular Medicine - Author Guidelines

Reporting Checklist for Life Science Articles (updated January
This checklist is adapted from Materials Design Analysis Reporting (MDAR) Checklist for Authors. MDAR establishes a minimum set of requirements in transparent reporting in the life sciences (see Statement of Task: 10.31222/osf.io/9sm4x). Please follow the journal's guidelines in preparing your
Please note that a copy of this checklist will be published alongside your article.

Abridged guidelines for figures
1. Data
The data shown in figures should satisfy the following conditions:
- the data were obtained and processed according to the field's best practice and are presented to reflect the results of the experiments in an accurate and unbiased manner.
- ideally, figure panels should include only measurements that are directly comparable to each other and obtained with the same assay.
- plots include clearly labeled error bars for independent experiments and sample sizes. Unless justified, error bars should not be shown for technical
- if n<5, the individual data points from each experiment should be plotted. Any statistical test employed should be justified.
- Source Data should be included to report the data underlying figures according to the guidelines set out in the authorship guidelines on Data

2. Captions
Each figure caption should contain the following information, for each panel where they are relevant:
- a specification of the experimental system investigated (eg cell line, species name).
- the assay(s) and method(s) used to carry out the reported observations and measurements.
- an explicit mention of the biological and chemical entity(ies) that are being measured.
- an explicit mention of the biological and chemical entity(ies) that are altered/varied/perturbed in a controlled manner.
- the exact sample size (n) for each experimental group/condition, given as a number, not a range;
- a description of the sample collection allowing the reader to understand whether the samples represent technical or biological replicates (including how many animals, litters, cultures, etc.).
- a statement of how many times the experiment shown was independently replicated in the laboratory.
- definitions of statistical methods and measures:
  - common tests, such as t-test (please specify whether paired vs. unpaired), simple χ2 tests, Wilcoxon and Mann-Whitney tests, can be unambiguously identified by name only, but more complex techniques should be described in the methods section;
  - are tests one-sided or two-sided?
  - are there adjustments for multiple comparisons?
  - exact statistical test results, e.g., P values = x but not P values < x;
  - definition of 'center values' as median or average;
  - definition of error bars as s.d. or s.e.m.

Please complete ALL of the questions below.
Select "Not Applicable" only when the requested information is not relevant for your study.

Materials

Newly Created Materials	Information included in the manuscript?	In which section is the information available? (Reagents and Tools Table, Materials and Methods, Figures, Data Availability Section)
New materials and reagents need to be available; do any restrictions apply?	Yes	Reagents and Tools Table, Methods

Antibodies	Information included in the manuscript?	In which section is the information available? (Reagents and Tools Table, Materials and Methods, Figures, Data Availability Section)
For **antibodies** provide the following information:<br>- Commercial antibodies: RRID (if possible) or supplier name, catalogue number and or/clone number<br>- Non-commercial: RRID or citation	Yes	Reagents and Tools Table

DNA and RNA sequences	Information included in the manuscript?	In which section is the information available? (Reagents and Tools Table, Materials and Methods, Figures, Data Availability Section)
**Short novel DNA or RNA including primers, probes**: provide the sequences.	Yes	Reagents and Tools Table

Cell materials	Information included in the manuscript?	In which section is the information available? (Reagents and Tools Table, Materials and Methods, Figures, Data Availability Section)
**Cell lines:** Provide species information, strain. Provide accession number in repository **OR** supplier name, catalog number, clone number, and/**OR** RRID.	Yes	Reagents and Tools Table, Methods
**Primary cultures:** Provide species, strain, sex of origin, genetic modification status.	Not Applicable	
Report if the cell lines were recently **authenticated** (e.g., by STR profiling) and tested for mycoplasma contamination.	Yes	Methods

Experimental animals	Information included in the manuscript?	In which section is the information available? (Reagents and Tools Table, Materials and Methods, Figures, Data Availability Section)
**Laboratory animals or Model organisms:** Provide species, strain, sex, age, genetic modification status. Provide accession number in repository **OR** supplier name, catalog number, clone number, **OR** RRID.	Yes	Reagents and Tools Table, Methods
**Animal observed in or captured from the field:** Provide species, sex, and age where possible.	Not Applicable	
Please detail **housing and husbandry conditions**.	Yes	Materials and Methods

Plants and microbes	Information included in the manuscript?	In which section is the information available? (Reagents and Tools Table, Materials and Methods, Figures, Data Availability Section)
**Plants:** provide species and strain, ecotype and cultivar where relevant, unique accession number if available, and source (including location for collected wild specimens).	Not Applicable	
**Microbes:** provide species and strain, unique accession number if available, and source.	Not Applicable	

Human research participants	Information included in the manuscript?	In which section is the information available? (Reagents and Tools Table, Materials and Methods, Figures, Data Availability Section)
If collected and within the bounds of privacy constraints report on age, sex and gender or ethnicity for all study participants.	Not Applicable	

Core facilities	Information included in the manuscript?	In which section is the information available? (Reagents and Tools Table, Materials and Methods, Figures, Data Availability Section)
If your work benefited from core facilities, was their service mentioned in the acknowledgments section?	Yes	Acknowledgments

Design

Study protocol	Information included in the manuscript?	In which section is the information available? (Reagents and Tools Table, Materials and Methods, Figures, Data Availability Section)
If study protocol has been **pre-registered, provide DOI in the manuscript**. For clinical trials, provide the trial registration number **OR** cite DOI.	Not Applicable	
Report the **clinical trial registration number** (at ClinicalTrials.gov or equivalent), where applicable.	Not Applicable	

Laboratory protocol	Information included in the manuscript?	In which section is the information available? (Reagents and Tools Table, Materials and Methods, Figures, Data Availability Section)
Provide DOI OR other citation details if **external detailed step-by-step protocols** are available.	Not Applicable	

Experimental study design and statistics	Information included in the manuscript?	In which section is the information available? (Reagents and Tools Table, Materials and Methods, Figures, Data Availability Section)
Include a statement about **sample size** estimate even if no statistical methods were used.	Yes	Methods
Were any steps taken to minimize the effects of subjective bias when allocating animals/samples to treatment (e.g. **randomization procedure**)? If yes, have they been described?	Yes	Methods
Include a statement about **blinding** even if no blinding was done.	Yes	Methods
Describe **inclusion/exclusion criteria** if samples or animals were excluded from the analysis. Were the criteria pre-established? If sample or data points were omitted from analysis, report if this was due to attrition or intentional exclusion and provide justification.	Yes	Methods, Figure Legends
For every figure, are **statistical tests** justified as appropriate? Do the data meet the assumptions of the tests (e.g., normal distribution)? Describe any methods used to assess it. Is there an estimate of variation within each group of data? Is the variance similar between the groups that are being statistically compared?	Yes	Methods, Figure Legends

Sample definition and in-laboratory replication	Information included in the manuscript?	In which section is the information available? (Reagents and Tools Table, Materials and Methods, Figures, Data Availability Section)
In the figure legends: state number of times the experiment was **replicated** in laboratory.	Yes	Figure Legends
In the figure legends: define whether data describe **technical or biological replicates**.	Yes	Figure Legends

Ethics

Ethics	Information included in the manuscript?	In which section is the information available? (Reagents and Tools Table, Materials and Methods, Figures, Data Availability Section)
Studies involving **human participants**: State details of **authority granting ethics approval** (IRB or equivalent committee(s), provide reference number for approval.	Not Applicable	
Studies involving **human participants**: Include a statement confirming that **informed consent** was obtained from all subjects and that the experiments conformed to the principles set out in the WMA Declaration of Helsinki and the Department of Health and Human Services Belmont Report.	Not Applicable	
Studies involving **human participants:** For publication of **patient photos**, include a statement confirming that consent to publish was obtained.	Not Applicable	
Studies involving experimental **animals**: State details of **authority granting ethics approval** (IRB or equivalent committee(s), provide reference number for approval. Include a statement of compliance with ethical regulations.	Yes	Methods
Studies involving **specimen and field samples:** State if relevant **permits** obtained, provide details of authority approving study; if none were required, explain why.	Not Applicable	

Dual Use Research of Concern (DURC)	Information included in the manuscript?	In which section is the information available? (Reagents and Tools Table, Materials and Methods, Figures, Data Availability Section)
Could your study fall under dual use research restrictions? Please check biosecurity documents and list of **select agents and toxins** (CDC): https://www.selectagents.gov/sat/list.htm	Not Applicable	
If you used a select agent, is the security level of the lab appropriate and reported in the manuscript?	Not Applicable	
If a study is subject to dual use research of concern regulations, is the name of the **authority granting approval and reference number** for the regulatory approval provided in the manuscript?	Not Applicable	

Reporting

The MDAR framework recommends adoption of discipline-specific guidelines, established and endorsed through community initiatives. Journals have their own policy about requiring specific guidelines and recommendations to complement MDAR.

Adherence to community standards	Information included in the manuscript?	In which section is the information available? (Reagents and Tools Table, Materials and Methods, Figures, Data Availability Section)
State if relevant guidelines or checklists (e.g., **ICMJE, MIBBI, ARRIVE, PRISMA**) have been followed or provided.	Not Applicable	
For **tumor marker prognostic studies,** we recommend that you follow the **REMARK** reporting guidelines (see link list at top right). See author guidelines, under 'Reporting Guidelines'. Please confirm you have followed these guidelines.	Not Applicable	
For **phase II and III randomized controlled trials**, please refer to the **CONSORT** flow diagram (see link list at top right) and submit the CONSORT checklist (see link list at top right) with your submission. See author guidelines, under 'Reporting Guidelines'. Please confirm you have submitted this list.	Not Applicable	

Data Availability

Data availability	Information included in the manuscript?	In which section is the information available? (Reagents and Tools Table, Materials and Methods, Figures, Data Availability Section)
Have **primary datasets** been deposited according to the journal's guidelines (see 'Data Deposition' section) and the respective accession numbers provided in the Data Availability Section?	Not Applicable	
Were **human clinical and genomic datasets** deposited in a public access-controlled repository in accordance to ethical obligations to the patients and to the applicable consent agreement?	Not Applicable	
Are **computational models** that are central and integral to a study available without restrictions in a machine-readable form? Were the relevant accession numbers or links provided?	Yes	Data Availability; https://github.com/YvesNomine/SPR_Kinetics_Analysis/
If publicly available data were reused, provide the respective **data citations in the reference list**.	Yes	References
