# Supplementary material for: Pharmacological inhibition of UBASH3B induces mitotic arrest and selectively reduces tumor growth
Source: EMBO Mol Med. 2026 Sep 22;18(11):4260–99. doi: 10.1038/s44321-026-00519-0 (PMC13748553; doi:10.1038/s44321-026-00519-0)

241213_D_p132_3AFL-MBP_CoeffMBP0.35_CoeffNorm_A_L10.4125

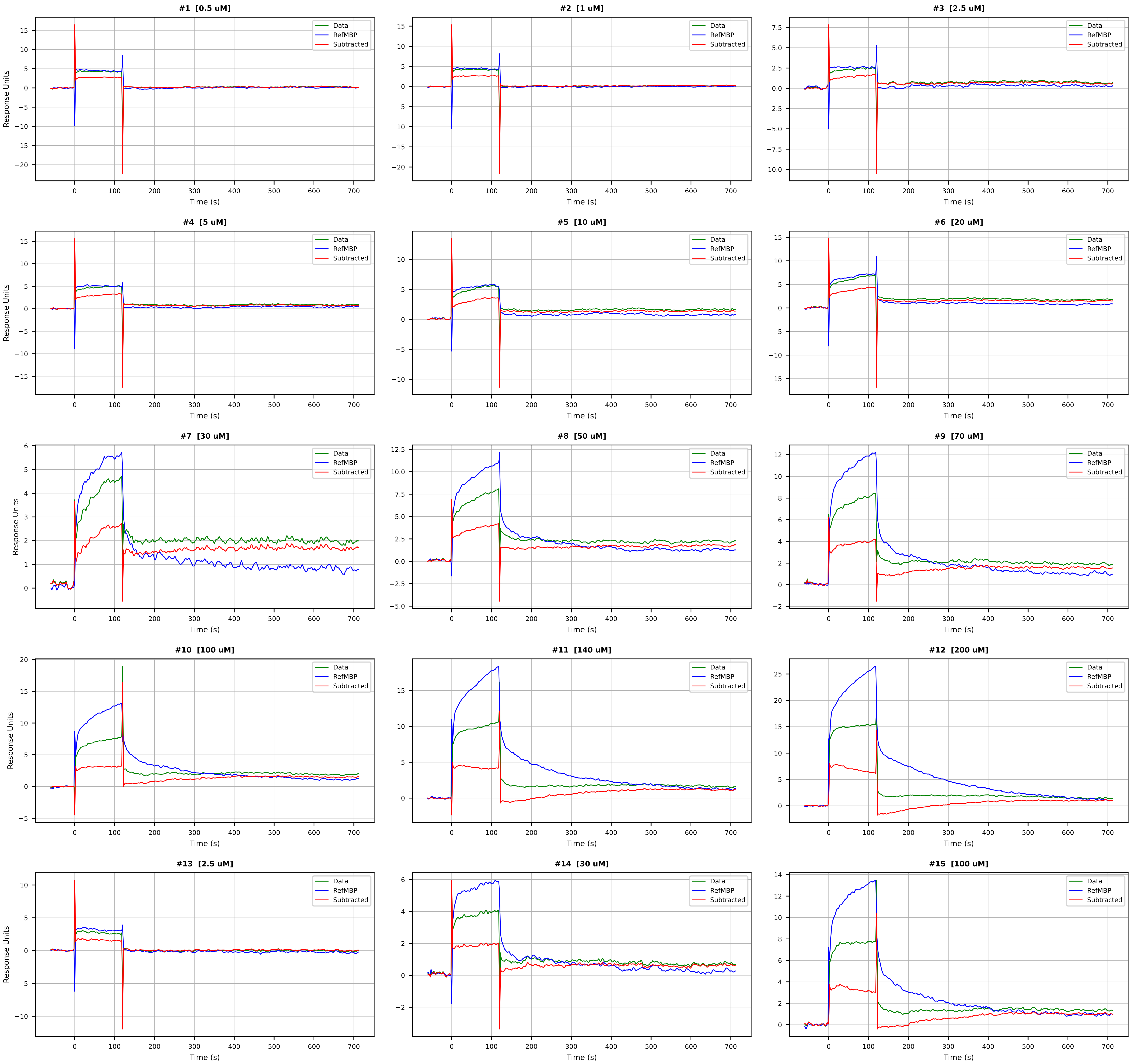

Supplement: Supplementary file 4 [file 44321_2026_519_MOESM10_ESM.zip › EMM-2025-22751_ SourceData_Figure 4/4A/UBASHIN5_UBASH3A FL.pdf]
