# Supplementary material for: Pharmacological inhibition of UBASH3B induces mitotic arrest and selectively reduces tumor growth
Source: EMBO Mol Med. 2026 Sep 22;18(11):4260–99. doi: 10.1038/s44321-026-00519-0 (PMC13748553; doi:10.1038/s44321-026-00519-0)

**241216_D_p132_3BFL-MBP_CoeffMBP1.0_CoeffNorm_A_L29.0**

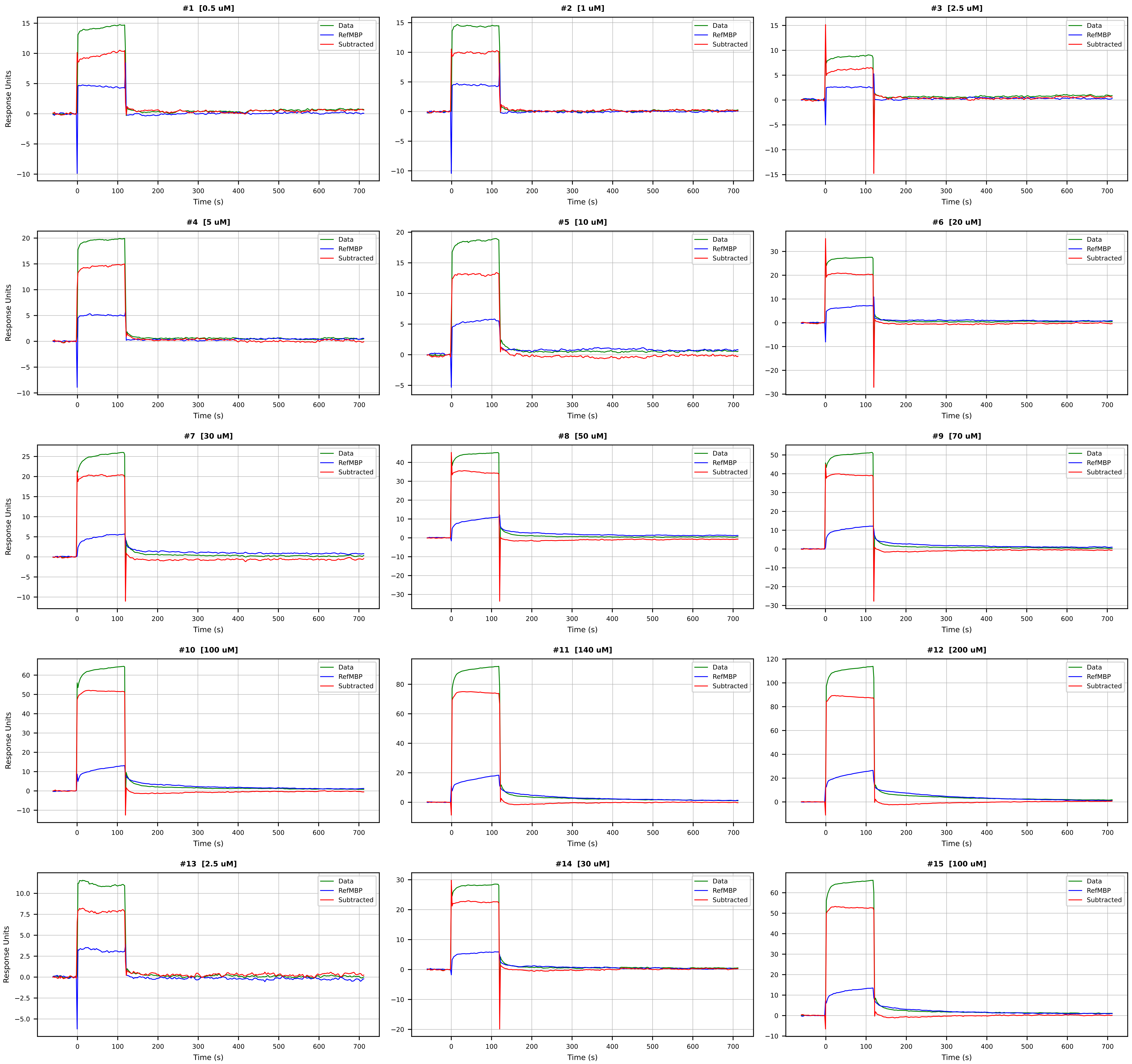

Supplement: Supplementary file 4 [file 44321_2026_519_MOESM10_ESM.zip › EMM-2025-22751_ SourceData_Figure 4/4A/UBASHIN5_UBASH3B FL.pdf]
