# Supplementary material for: Pharmacological inhibition of UBASH3B induces mitotic arrest and selectively reduces tumor growth
Source: EMBO Mol Med. 2026 Sep 22;18(11):4260–99. doi: 10.1038/s44321-026-00519-0 (PMC13748553; doi:10.1038/s44321-026-00519-0)
Supplement: Supplementary file 14 [file 44321_2026_519_MOESM6_ESM.zip › EV4 movie/Figure Legend for Movie EV4.docx]

**Figure Legend for Movie EV4**

HeLa cells stably expressing H2B-mCherry treated with UBASHIN-3 were synchronized in mitosis with DTBR and analyzed by spinning disk time lapse microscopy. Chromosomes are shown in green. Images were acquired for 8 h every 5-10 min as Z-stacks spanning 25 µm with 2 µm steps.
